# Global Perspective of *Legionella* Infection in Community-Acquired Pneumonia: A Systematic Review and Meta-Analysis of Observational Studies

**DOI:** 10.3390/ijerph19031907

**Published:** 2022-02-08

**Authors:** Frances F. Graham, Norah Finn, Paul White, Simon Hales, Michael G. Baker

**Affiliations:** 1 Department of Health, University of Otago, Wellington 6242, New Zealand; simon.hales@otago.ac.nz (S.H.); Michael.Baker@otago.ac.nz (M.G.B.); 2 Victorian Cancer Registry, Victorian Cancer Council, Melbourne, VIC 3004, Australia; norah.finn@cancervic.org.au; 3 World Health Organization, Bangkok 11000, Thailand; paulwhiteships@hotmail.com

**Keywords:** *Legionella*, community-acquired pneumonia, Legionnaires’ disease, systematic review, meta-analysis

## Abstract

Legionnaires’ disease (LD) (*Legionella*) is a common cause of community-acquired pneumonia (CAP) in those requiring hospitalization. Geographical variation in the importance of *Legionella* species as an aetiologic agent of CAP is poorly understood. We performed a systematic review and meta-analysis of population-based observational studies that reported the proportion of *Legionella* infection in patients with CAP (1 January 1990 to 31 May 2020). Using five electronic databases, articles were identified, appraised and reported in accordance with the Preferred Reporting Items for Systematic Reviews and Meta-Analysis (PRISMA) guidelines. The quality of the included studies was assessed using the Newcastle–Ottawa Scale. Univariate and multivariate meta-regression analyses were conducted using study design, WHO region, study quality and healthcare setting as the explanatory variables. We reviewed 2778 studies, of which 219 were included in the meta-analysis. The mean incidence of CAP was 46.7/100,000 population (95% CI: 46.6–46.8). The mean proportion of *Legionella* as the causative agent for CAP was 4.6% (95% CI: 4.4 to 4.7). Consequently, the mean *Legionella* incidence rate was 2.8/100,000 population (95% CI: 2.7–2.9). There was significant heterogeneity across all studies *I*^2^ = 99.27% (*p* < 0.0001). After outliers were removed, there was a decrease in the heterogeneity (*I*^2^ = 43.53%). *Legionella* contribution to CAP has a global distribution. Although the rates appear highest in high income countries in temperate regions, there are insufficient studies from low- and middle-income countries to draw conclusions about the rates in these regions. Nevertheless, this study provides an estimate of the mean incidence of *Legionella* infection in CAP, which could be used to estimate the regional and global burden of LD to support efforts to reduce the impact of this infection as well as to fill important knowledge gaps.

## 1. Introduction

Community-acquired pneumonia (CAP) is an acute infection of the pulmonary parenchyma that develops in persons outside of a hospital or health care facilities, such as nursing homes, hemodialysis centers and outpatient clinics. CAP is a leading cause of morbidity and mortality both in high- and low-income countries [1] making it of great epidemiological significance globally with noted seasonal [2] and regional [3] variations. Over 100 microorganisms have been identified as causative agents in CAP [4]. Historically, influenza and rhinovirus were the most commonly detected viral pathogens [5]. More recently, coupled with the increasing availability of molecular diagnostics, such as the polymerase chain reaction (PCR), the identification of bacterial pathogens in the etiology of CAP has increased [6]. Globally, *Streptococcus pneumoniae* is the most commonly recognized bacterial pathogen in CAP [7,8].

Recent studies identified *Legionella* species (spp.) as being among the four most frequent microbial causes of hospitalizations due to CAP [9,10], accounting for 2% to 15% of patients as the cause of severe CAP requiring hospitalization [11,12]. Few studies have reported the incidence of *Legionella* as a cause of CAP outside the hospital setting. One study found *Legionella* to be equally common in outpatients and inpatients [13]. Furthermore, in an era of changing climate conditions globally, seasonal variations of microbial etiology are important for the future CAP, since *S. pneumoniae* and *Legionella* are clearly subject to seasonal variations where correlations with humidity and rainfall have been reported [14,15]. Despite several narrative reviews of the epidemiology of legionellosis (or Legionnaires’ disease (LD)), a severe infection caused by *Legionella* spp.) [16,17,18,19,20,21,22,23], the global epidemiology of legionellosis is not well characterized. Published systematic reviews that have synthesized results of CAP etiology studies focusing on bacterial pathogens, including *Legionella* spp., are sparse and only performed among adults and limited to a specific WHO region [24]. Other systematic reviews focused on the prevalence of bacterial pathogens, including *Legionella* of patients with CAP, particularly in the outpatient primary care setting but excluded studies set in low- or middle-income countries (based on the Organisation for Economic Cooperation and Development (OECD) criteria) [25]. In the present analysis, we take the novel approach of looking at the global epidemiology of CAP as a way of then understanding and quantifying the contribution of legionellosis to that burden. In doing so, this review also seeks to augment a previous global systematic review and meta-analysis of seroprevalence studies of *Legionella* infection [26].The aim of this systematic review and meta-analysis is firstly to assess the contribution of *Legionella* infection in CAP, that is, those studies that measure the proportion of cases that are attributed to microbiologically confirmed *Legionella* infection. As *Legionella* cannot be distinguished clinically or radiographically from other types of pneumonia, any diagnosis of *Legionella* relies on the use of special laboratory tests [4,7], resulting in *Legionella* spp. being underdiagnosed in the etiology of CAP. Consequently, a second aim is to describe the many factors that might influence the sensitivity of *Legionella* diagnoses among CAP studies [27]. It is important to understand the causes of CAP to support the development of suitable prevention programs as well as selecting optimal antibiotic treatments [27].

## 2. Materials and Methods

### 2.1. Search Strategy and Eligibility Criteria

Our systematic review and meta-analysis followed the quality standards for reporting meta-analyses of observational studies in epidemiology [28] and PRISMA (Preferred Reporting Items for Systematic Reviews and Meta-Analyses) guidelines [29] (Figure S1). The study protocol is also registered with PROSPERO (CRD 42021259323). We examined articles published before 31 May 2020 in Medline (Ovid), Embase, Scopus, LILACS and the Cochrane Library using a modified version of the PRISMA search strategy. The following sources of gray literature were also searched until 31 May 2020: Te Puna, Kiwi Research Information Service, Proquest Dissertations and Theses, Index to Theses, OCLC FirstSearch: WorldCat, EThOS (Electronic Theses Online Service), OAIster, DART-Europe E-Theses Portal, Theses Canada, Trove, as well as GreyLit.org and OpenGrey.eu. PubMed (publisher: U.S.A. National Library of Medicine) was searched in addition to the MEDLINE database because the former also covers publications that are electronically published ahead of print. Figure S2 shows the search strategy. 

**Figure 1:**
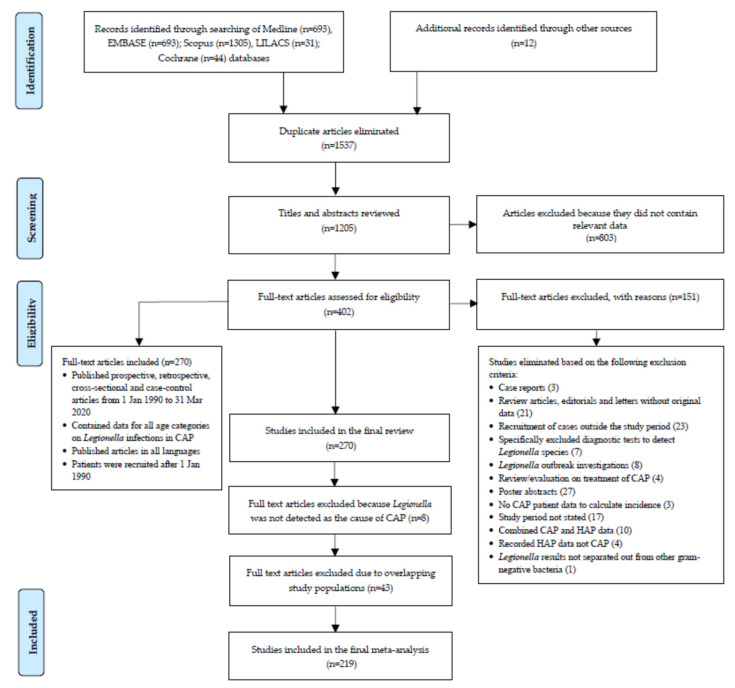
Flowchart of systematic literature review and study selection for meta-analysis.

We estimated the annual incidence for *Legionella* spp. infections in CAP in both outpatient and hospitalized patients using the search strategy in Figure 1. We included observational studies and reported data for the calculation of the incidence of *Legionella* infection in CAP in at-risk individuals for all age categories. The search consisted of four components: 1. ‘pneumonia’ or ‘respiratory tract infection’ in the title; 2. geographical terms anywhere in the citation using names of all countries; 3. patient recruitment from 1 January 1990 and 4. observational studies that included patients with a diagnosis of CAP based on confirmation culture, polymerase chain reaction (PCR), serology or urine antigen testing (UAT) and reported the proportion of *Legionella* infection.

All languages were eligible for inclusion and no publication restrictions were applied. All non-English articles were screened using Google Translate [30] or by a native speaker. The title and abstract of all the retrieved citations were reviewed, including studies with data on subgroups, such as the elderly or children, and the full text was retrieved if the abstract suggested the article contained data on *Legionella* in CAP etiology. The studies of CAP carried out on specific immunocompromised populations, such as patients with chronic obstructive pulmonary disease (COPD), HIV infection or diabetes mellitus, were reviewed. The reference lists of all the retrieved papers were manually searched for additional relevant studies. Where appropriate, some authors were contacted for further information, such as the study period. In circumstances where the same data were reported in more than one paper, the earliest published paper was selected. Legionellosis data were extracted and analyzed based on the number of cases, not the number of samples. 

The exclusion criteria were as follows. (a) Studies addressing other aspects of CAP, such as hospital-acquired pneumonia (HAP), that is, patients with pneumonia occurring 48 h or more after hospital admission. Hospitalized patients with a diagnosis of ventilator-associated pneumonia were also excluded. However, in circumstances where studies reported results containing both CAP and HAP data [31,32,33,34,35] only Legionellosis data specific to CAP was included in the meta-analysis. (b) Poster abstracts, brief communications, case reports, cases series, reviews/evaluations, editorials, correspondence without original data and laboratory-based studies of CAP (c) studies, which recruited CAP patients pre 1 January 1990. (d) Study period not stated. (e) The etiological role of *Legionella* was not included in the study design [36]. The complete search strategy is detailed in the Supplementary Appendix. Available gray literature, which predominantly comprised of national and jurisdictional reports describing the routine treatment and management of CAP, was not considered as useful for our review.

### 2.2. CAP Definition and Study Population

Studies on the microbial etiology of CAP show that the portion of causative pathogens varies geographically and by the study populations used, concurrent epidemics, microbiological techniques and definitions of the etiological diagnosis of CAP [37]. For the purposes of this review, we did not specify a CAP definition (including severe CAP) largely because it included such a heterogeneous patient group [38]. Rather, we accepted the authors’ definition on the assumption that they gathered CAP data and used diagnostic tests to accurately classify CAP patients as having *Legionella*. Most studies, as a minimum, defined CAP as a chest radiograph showing new infiltrates or consolidation that could not be attributed to some other etiology, in the presence of acute onset fever, cough or sputum production.

Population estimates used to calculate annual incidence were derived from either published, national census data or mid-year estimates. The population figures were adjusted to reflect the age demographic inclusion criteria of each study.

### 2.3. Data Extraction and Quality Assessment of Studies

Data were extracted from each included study by one reviewer (FG) onto a standardized form that included data related to study characteristics (first author, year of publication, study design, WHO region, location, characteristics of study participants (number, patient recruitment period, mean or median age and male gender)) and the outcome of measures of interest, including the effect size of the data incidence of *Legionella* on CAP and case fatality risk (CFR). The healthcare setting was divided into five distinct settings: (1) inpatients not admitted to ICU; (2) ICU admitted patients; (3) outpatients with no-comorbidities; (4) outpatients with cardiopulmonary disease or other modifying factors; and (5) both inpatients (including ICU) and outpatients (Supplementary Table S1). We also reviewed studies to ascertain whether there was a correlation between meteorological conditions and sporadic cases of LD.

In order to assess the methodological quality of the observational studies, we used, as a validation tool, the Newcastle–Ottawa Scale (NOQAS) (Supplementary Tables S1 and S2) to categorize quality in four domains: participant selection, comparability of populations and outcome assessment. The methodological quality of the selected studies was assessed with an overall score ranging from 0 to 9 (highest level of quality). Study quality was classified according the study score into poor (0–3), moderate (4–6) and high (7–9) [39,40]. Poor methodological quality was not an exclusion criterion.

### 2.4. Statistical and Sensitivity Analysis

For all the meta-analytical procedures and graphical presentations, we used the meta, *metafor*, *metagen* and *dmetar* packages in R statistical software (version 4.1.1 with R-studio version 1.4.1717) in accordance with the guide by Harrer et al., 2019 [41]. When a significant Q-test [42] indicated heterogeneity across studies (*p* < 0.10) or the *I*^2^ was above 50%, the random-effects model was used for the meta-analysis; otherwise, the fixed-effect model was used [43]. The potential for publication bias and small-study effects were assessed visually by using funnel plots (1/standard error) by study effect size (mean difference). Egger’s linear regression analyses were also used to further assess the presence of publication bias. A Baujat plot was used to detect outlier studies in our meta-analyses [44]. All the results were considered statistically significant when *p* < 0.05, which may indicate publication bias [45]. To account for this, we performed the trim and fill method as described by Duval and Tweedie trim [46]. Further analysis was conducted to determine the influence of outliers on the pooled estimates using the *dmetar* R command [47].

For the sensitivity analyses, we performed the leave-one-out test [48]. After outlier studies were identified and excluded, a repeat meta-analysis was performed to assess the heterogeneity. To explain the residual heterogeneity and to assess the possible associations between population co-variables and study outcome, a random-effects univariate meta-regression was performed to examine the association between the change in *Legionella* incidence and each of the following study-level variables (moderators): WHO region, study design and quality (NOQAS score) and healthcare setting. The case-control design, WHO region (Africa) and healthcare setting (outpatients with no comorbidities) were the reference categories. Furthermore, multivariate analyses were performed in a meta-regression model [49]. The *p*-values for differences in the effects between the covariates were generated using the *metagen* function of R version 4.1.1.

## 3. Results

### 3.1. Search Results and Study Selection

**Figure 2:**
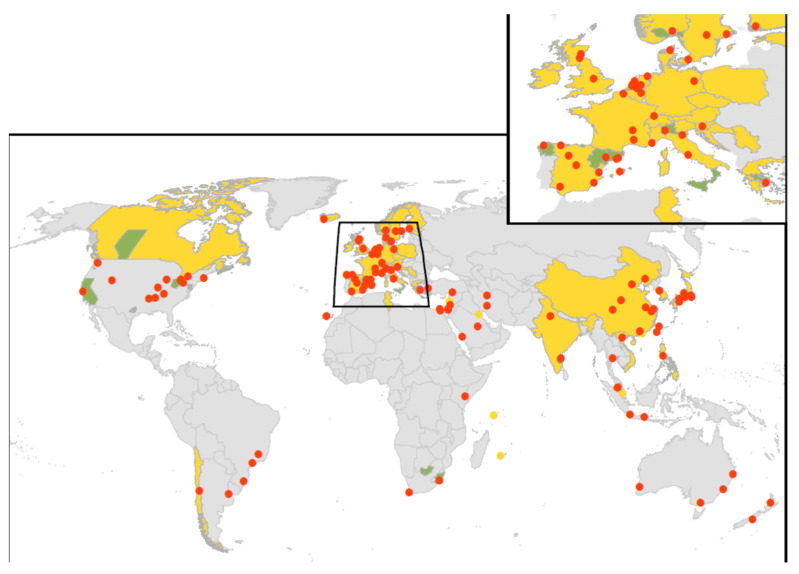
Spatial distribution of CAP studies. Studies based on representative population sampling are considered as representative (yellow). Studies based on the records from multiple hospitals in different cities and studies based on records from laboratories that perform diagnostic testing for *Legionella* for patients throughout a region or country are considered as non-representative (green). Studies based in hospital settings whose patient populations are localized to a single city are considered localized (red points).

Supplementary Figure S1 summarizes the results of the search strategy. A further explanation of the results, including study characteristics (Table S1), as outlined in Figure 1, is provided in the Supplementary Material. The results for 219 individual studies included in the meta-analysis are shown in Figure 2 and in Supplementary Table S2. Figure 2 shows that many countries did not have eligible studies, including countries that make up the whole of Eastern Europe, Africa, Central America and the Pacific Islands. These gaps highlighted the minimal data available to assess the burden of *Legionella* infection as a cause of CAP in low-income countries, because patients are sub-optimally treated for higher priority diseases, such as TB or HIV, at the time of admission [50]. Despite several published studies, there was also minimal data from the United Kingdom, Canada and the United States. The largest number of studies undertaken were in western Europe (59.2%) with a bias towards Spain, which recorded 53 studies (24.2%).

The median duration for all *Legionella* CAP studies considered was 23 months (interquartile range: 12–36 months). The published studies were prospective studies (n = 173), retrospective studies (n = 33), cross sectional (n = 7) and case–control studies (n = 6). There were 139 studies that were based on hospital settings where patient populations were localized to a single city; 34 studies were based on records from multiple hospitals in different cities throughout a region; and 41 studies were multicenter studies representing the whole of a country (Canada, Spain, Slovenia, France, Switzerland, The Netherlands, Italy, Germany, Japan, Kuwait, Tunisia, Taiwan, South Korea, China, Chile, India, Vietnam, The Philippines and Singapore). Five large studies were multinational, and one was international, but the data from each country were not treated separately because the reported annual incidence of CAP caused by *Legionella* spp. was extremely low, ranging from 0.001–0.6 per 100,000 population in these studies [51,52,53,54,55,56]. The study size ranged from 15 to 7803 patients, with a median of 232 (interquartile range: 133–474) enrolled patients.

Most studies specified some exclusion criteria, generally related to (a) pneumonia not being the primary cause for hospital admission; (b) lung cancer or terminal illness; (c) distal to bronchial obstruction; (d) patients who had been in hospital within the previous 14 days, who were immunocompromised and (e) nursing home residents. We extracted data on the study country, sample size, diagnostic test used and age range for CAP studies, and the number of people with LD. This systematic review considered studies of CAP patients of all ages, as well as patients with tuberculosis or human immunodeficiency virus (HIV) infection.

Three studies observed a significant correlation between the lowest seasonal average temperature and polymicrobial pneumonia and pneumococcal pneumonia; conversely, *L. pneumophila* was more common when the temperatures were higher [2,3,9].

### 3.2. Legionella Epidemiology

**Figure 3:**
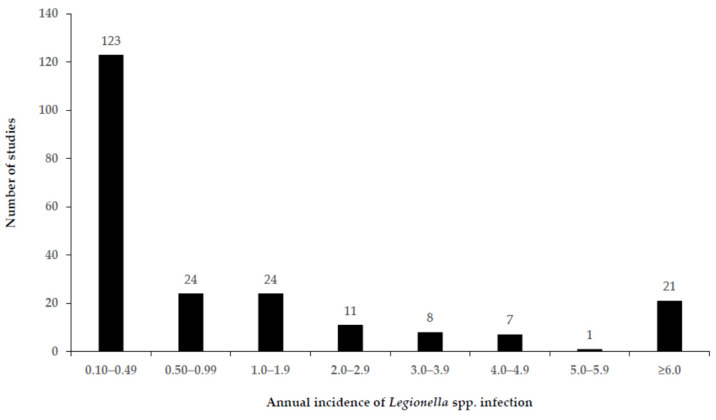
Annual incidence of *Legionella* spp. infection (cases/100,000 population) among CAP patients (all ages) from 219 included studies for meta-analysis.

Based on an R command for the overall effect size, the proportion of *Legionella* spp. as the causative agent of CAP during the study period was 4.6% (95% CI: 4.4–4.7) (Table 1), with the highest proportion recorded in Eastern Mediterranean (9.7% 95% CI 8.5–10.9). Of the healthcare settings, ICU patients only recorded the highest proportion of *Legionella* spp. as the causative agent of CAP (9% 95% CI 3–14). Supplementary Table S2 and Figure S3 show that the annual incidence of *Legionella* infection, because of its global distribution, was highly variable in different studies, ranging from 0.001 and 147.4 per 100,000 population of CAP (the mean incidence rate was 2.8 per 100,000 for all regions (Table 1); 56.2% of the studies reported an annual incidence of <0.49′; and 37% of studies reported an annual incidence of ≤0.10, Figure 3) identified in studies, which had recruited patients from 1 January 1990 (Supplementary Table S2).

Of the 196 studies that recorded the *Legionella* spp. detected, *L. pneumophila* was identified as the causative pathogen in 95.4% of the studies of patients with CAP requiring hospitalization and was associated with high morbidity. Non-*pneumophila Legionella* spp. represented only 4.6% (*L. longbeachae* 3.1%) of the etiology detected in the 125,764 patients with CAP. As a result of the high frequency of respiratory failure, patients with *Legionella* CAP are significantly more likely to be admitted to an intensive care unit [57]. The mean CFR was 22.6% (SD ± 39.5), which was similar to the CFR in healthcare associated cases of 18% [58]–31.7% reported in the literature [59]. The median proportion of males in the 179 studies that reported sex was 128 (71.5%). 

### 3.3. Sensitivity Analyses

Table 1 presents a summary of the studies showing the proportions of *Legionella* spp. as the causative agent for CAP. There was significant heterogeneity across all the studies that met the criteria (*I*^2^ = 99.27% (*p* < 0.0001), (Table 2). Africa was the only region in which there was moderate heterogeneity observed for both random-effects and fixed-effects models (Table 2). Figures S4–S9 show each of the forest plots by the WHO region. Observational studies reported major differences in the frequencies of *Legionella* spp. causing CAP. These differences may be due to the variances in the locations studied, the specific patient populations included and the extent and nature of the diagnostic tests used. Using the Baujat plot and the ‘find.outliers’ function from the *dmetar* package to identify the outliers (Figure S10), we re-ran our initial analysis by excluding the identified outliers. The overall heterogeneity was substantially reduced (*I*^2^ = 43.53%).

### 3.4. Univariate and Multivariate Meta-Regression

Taken together, the univariate meta-regression showed no significant association between the changes in *Legionella* incidence and study design, WHO region, study quality (overall) and healthcare setting (Table 3). There was an exception for the Eastern Mediterranean region, a significant moderator of the WHO region. Slope coefficients did not differ significantly from zero (*p*  >  0.05).

Similar to the univariate model, the multivariate meta-regression did not reveal any significant change between *Legionella* incidence and study design, WHO region, study quality (overall) and healthcare setting, except for the Eastern Mediterranean region (WHO region) (Table 3).

### 3.5. Publication Bias

The *p*-value for the Egger’s test confirmed that there was significant bias and therefore publication bias (*p* ≤ 0.0001). The funnel plot (Figure S11—WHO region (European) study Tilley 2009 was identified as the key outlier in the funnel plot) appears as asymmetrical and suggestive of publication bias, which cannot be completely excluded as a factor of influence on the present meta-analyses. After small population studies were excluded, the trim and fill method result did not change the parameter estimates for *Legionella*’s contribution to CAP (Figure S12).

## 4. Discussion

This meta-analysis found that on average, 4.6% of CAP was caused by *Legionella* species. Consequently, the mean incidence rate of *Legionella* infection was 2.8/100,000 population. The mean CFR was 22.6%, which may be an over-estimate as *Legionella* infection is an underdiagnosed disease [59].

The large heterogeneity observed in the incidence estimates for *Legionella* infections is not unexpected considering the multitude of potential sources of the measurement error, including the variable definitions of CAP, patients’ characteristics, diagnostic methods and criteria used for diagnosis. For example, there was an impact of small studies (19.2% had a size of less than 100 patients) with a higher proportion of *Legionella* in areas where it was anticipated it would be more severe, namely those patients admitted to ICU (9%: 95% CI 0.03–0.14). The CAP etiologies were determined via a range of diagnostic tests, including culture, urine antigen, serology and molecular nucleic acid testing (Table S2). Over half of the studies (57.2%) that examined the etiology of *Legionella* in CAP used urinary antigen assays to identify the organism. Fifty-two percent of the studies that used sputum culture, also reported in their microbiological evaluation some quality criteria to improve reliability, although the criteria used varied.

The knowledge of pathogens causing CAP is important for the selection of antimicrobial treatment [9]. Despite the differences in geographic location, patient population and laboratory methods applied, this systematic review with meta-analysis represents a synthesis of published CAP etiology studies revealing several important findings: (i) *Legionella* spp. were found to be a common bacterial etiology of CAP (4.6%: 95% CI 4.4–4.7). (ii) The annual incidence of *Legionella* infection, in spite of its global diffusion, was highly variable in different studies (Figure 3). This present study provides an estimate of the mean incidence of *Legionella* infection in CAP, which could be used to estimate the global public health burden of LD to support improved prevention and management interventions. (iii) *L. pneumophila* was identified as an important agent for severe CAP after *S. pneumoniae*. This result is in line with global findings [13,60,61,62], although one study found no *L. pneumophila* positive cases among 373 patients but detected *L. longbeachae* infection in 3.8% of patients using serology and PCR [63].

The spatial differences in the importance of non-*pneumophila Legionella* spp. as pathogens are under-recognized, in part due to the available diagnostic tests, such as the urinary antigen test that is biased towards the detection only of *L. pneumophila* serogroup 1 infections [10,11]. The widespread introduction of *Legionella* urine antigen testing into hospital laboratories, globally has resulted in a decline in the use of culture or other serological tests [64]. Some authors suggested that the total reliance on this diagnostic test may miss up to 40% of LD cases [16], while others recommended that it should be used routinely in those with severe CAP and/or associated epidemiological factors, such as during an outbreak or post travel [65]. In addition, the non-*pneumophila* species, such as *L. longbeachae*, does not grow on blood agar media and is usually not detected by sputum Gram-stain or blood culture [66]. Yet, in countries, such as New Zealand, Australia and Scotland, species, including *L. longbeachae* and other non-*pneumophila Legionella* spp., are the more prevalent causes of infection for which serology or PCR may be used as the primary diagnostic test [23]. Since none of the current diagnostic tests to detect *Legionella* infection have sufficient sensitivity to guide definitive therapy for CAP, clinicians must treat possible *Legionella* infections empirically [67]. (iv) Seasonal variation was observed for *L. pneumophila* infection in CAP with the risk being lower in winter and spring than in summer [2,9]. As a result, there is a perception that *Legionella* is more important in Mediterranean countries and is uncommon in northern or southern temperate zones, other than in travelers from these countries or in the context of a local source of infection via an outbreak [11]. 

The main limitation of this study is the absence of data from large parts of the world, and the small number of patients enrolled from countries representing Africa and Asia, countries with the highest pneumonia burden. As a result, this limited the generalizability of our findings. We attempted to reduce bias by including both English and non-English studies and by attempting to identify cases of multiple publication. Overall, the heterogeneity of studies was high (*I*^2^ = 99.27%), which led us to explore whether there were potential contributors for such heterogeneity. We thus performed an exclude-one sensitivity analysis and examined the impact of outliers to locate the source of heterogeneity and rectify this, which resulted in a decrease in heterogeneity (*I*^2^ = 43.53%).

As part of the burden of respiratory infection, CAP is well recognized to be a leading cause. No detrimental effects of increased *Legionella* incidence could be observed in the meta-regression possibly owing to the low number of studies, many countries having no eligible studies and the inherent biases of the method. Therefore, any findings should be interpreted with caution. We included studies in which the outcome measures and definitions were based on defined criteria [68], including the acknowledgement that there is no consensus on what precise criteria are essential for the diagnosis of pneumonia [69]. Therefore, we did not exclude the studies in which a radiographic confirmation of pneumonia was an inclusion criterion to confirm and validate each case of CAP, particularly since several studies demonstrated a lack of agreement in the interpretation of chest radiographs bringing their role as the ultimate arbiter of CAP diagnosis into question [70]. Nevertheless, we anticipate that there may have been patients in whom the diagnosis may have been missed, particularly among those with milder symptoms, who were treated for CAP in a community setting. Few studies have focused on patients with pneumonia in primary care, in which *Legionella* among outpatients was found to be uncommon [71,72,73,74], although there are exceptions (von Baum et al., 2008) [13]. During a three-year period, only one known case of *L. pneumophila* infection occurred in the catchment area of a Swedish primary care center, but this patient was referred to and treated in the nearby hospital [75]. A limitation of these studies was that the etiology of pneumonia was determined by testing acute and convalescent serum samples for antibodies to *Legionella*. While once popular, globally one of the criticisms now leveled at serology, its use has significantly declined because it is not regarded as a useful rapid diagnostic method [76], compared to molecular methods, such as the polymerase chain reaction (PCR), which is increasingly being used in routine practice in many clinical settings due to more robust assays for the detection of other diseases in addition to *Legionella* [77]. The principal limitation of this study is the heterogeneity of the microbiological data (samples from different origins and diagnostic tests with different targets). In some studies, several methods were used to reach an etiologic diagnosis (for example, UAT, PCR, serology, and culture for *Legionella*), which made it difficult to determine what the number of patients had tested positive for each method. 

The Community-Acquired Pneumonia Organization initiative may offer a platform to investigators from Africa and Asia, to address some of the research questions in the area of *Legionella* in the pneumonia of HIV-infected patients [48] although one study found that *Legionella* spp. were a leading cause of CAP among HIV-negative patients [78]. Epidemiological factors, such as the time of year, may have also impacted the frequency of *Legionella* cases detected in CAP. The disease’s seasonality is known to increase the risk of infection, with most cases being reported during warm and humid weather, which tends to support pathogen survival, growth and the potential for aerosol exposures, increasing disease risk [78]. The median study duration for the studies included in our analysis was 23 months, which may not have been long enough to capture the long-term seasonal effects.

## 5. Conclusions

We performed a systematic review and meta-analysis using appropriate methodology but relied on data that are difficult to interpret mainly due to their methodological differences. Nevertheless, this review provided the first estimate of the mean incidence of *Legionella* infection in CAP, which can be used to estimate the regional and global burden of LD.

## Figures and Tables

**Table 1:** Summary of the contribution of *Legionella* spp. to CAP, CAP rates, and estimated *Legionella* rates globally and by the WHO region together with the confidence intervals.

WHO Region	#Cases/#Participants	*Legionella* Proportion (%)	95% CI	CAP Rate (per 100,000)	95% CI	*Legionella* Rate (per 100,000)	95% CI
All Regions	5723/125,764	4.6	(4.4–4.7)	46.7	(46.6–46.8)	2.8	(2.7–2.9)
Africa	48/2965	1.6	(1.1–2.1)	31.5	(30.1–32.4)	0.9	(0.1–1.8)
Eastern Mediterranean	248/2556	9.7	(8.5–10.9)	65.1	(64.7–65.5)	13.9	(13.5–14.3)
Europe	2529/56,537	4.5	(4.3–4.6)	62.9	(62.8–63.0)	2.4	(2.3–2.5)
South-East Asian	228/5267	4.3	(3.7–4.9)	132.7	(132.3–133.1)	3.4	(2.9–3.8)
The Americas	437/29,628	1.5	(1.3–1.6)	74.0	(73.7–74.3)	1.6	(1.3–1.9)
Western Pacific	1120/25,109	4.5	(4.2–4.7)	17.3	(17.1–17.5)	0.7	(0.5–0.9)

**Table 2:** Summary of sensitivity analyses.

Description (WHO Region)	#Cases/#Participants	Pool Effect Estimate	95% CI ^1^	*I*^2^ (%)	*p*-Value for Heterogeneity	Model
All Regions	5723/125,764	0.05	(0.04–0.06)	99.27%	<0.0001	Random-effects
Africa	48/2965	0.02	(0.01–0.03)		0.1092	Random-effects
			(0.01–0.03)		0.1092	Fixed-effects
Eastern Mediterranean	248/2556	0.09	(0.04–0.06)		<0.0001	Random-effects
Europe	2529/56,537	0.06	(0.04–0.06)		<0.0001	Random-effects
South-East Asian	228/5267	0.07	(0.04–0.06)		<0.0001	Random-effects
The Americas	437/29,628	0.03	(0.04–0.06)		<0.0001	Random-effects
Western Pacific	1120/25,109	0.02	(0.04–0.06)		<0.0001	Random-effects

^1^ CI, confidence interval.

**Table 3:** Univariate and multivariate meta-regression for change in *Legionella* incidence involving several study characteristics.

Univariate Analysis	Covariate	Number of Studies	β-Coefficient	95% CI	*p*-Value	Heterogeneity (*I*^2^) (%)	Multivariate Analysis	β-Coefficient	95% CI	*p*-Value	Heterogeneity (*I*^2^) (%) (For Full Multivariate Model
	**Study Design**										
	Cross sectional	7	0.0111	−0.0535 to 0.0756	0.7368	99.15%		0.0042	−0.0579 to 0.0663	0.8949	98.40%
	Prospective	173	− 0.0287	−0.0765 to 0.0190	0.2380			−0.0298	−0.0771 to 0.0175	0.2167	
	Retrospective	33	− 0.0063	−0.0571 to 0.0445	0.8082			0.0005	−0.0492 to 0.0501	0.9855	
	**WHO Region**										
	Eastern Mediterranean	15	0.0706	0.0213 to 0.1199	0.0050	98.83%		0.0633	0.0099 to 0.1167	0.0201	
	European	117	0.0376	−0.0049 to 0.0801	0.0830			0.0296	−0.0168 to 0.0760	0.2109	
	South-East Asian	12	0.0465	−0.0043 to 0.0973	0.0730			0.0448	−0.0119 to 0.1016	0.1217	
	The Americas	31	0.0238	−0.0214 to 0.0690	0.3023			0.0157	−0.0331 to 0.0645	0.5287	
	Western Pacific	38	0.0152	−0.0292 to 0.0595	0.5024			0.0124	−0.0358 to 0.0606	0.6139	
	**Study Quality Assessment**										
	Overall	219	−0.0001	−0.0048 to 0.0046	0.9655	99.23%		−0.0005	−0.0053 to 0.0043	0.8457	
	**Healthcare Setting**										
	Outpatients (with comorbidities)	5	−0.0020	−0.0571 to 0.0532	0.9446	99.01%		−0.0024	−0.0554 to 0.0505	0.9285	
	Inpatients (no ICU)	157	0.0167	−0.0186 to 0.0520	0.3531			0.0107	−0.0234 to 0.0449	0.5376	
	ICU patients only	18	0.0397	−0.0021 to 0.0532	0.0624			0.0352	−0.0054 to 0.0759	0.0893	

## Data Availability

Further information can be obtained from the corresponding author.

## Supplementary Materials

The following are available online at https://www.mdpi.com/article/10.3390/ijerph19031907/s1: Figure S1: The PRISMA checklist; Figure S2: Search strategy for community acquired pneumonia and *Legionella*; Table S1: Study characteristics summary included in the meta-analysis; Table S2: Studies of patient recruitment from 1 January 1990 defining the etiology of community-acquired pneumonia (CAP) and *Legionella* according to WHO region; Table S3: Patient and study characteristics of included studies where *Legionella* was not detected as the cause of infection; Table S4: Patient and study characteristics of excluded studies from the meta-analysis that reported data on overlapping participant populations; Figure S3: *Legionella* incidence of CAP studies, which recruited patients from 1 January 1990; Figure S4: Forest plot of European studies showing proportions of *Legionella* as the causative agent to CAP; Figure S5: Forest plot of Eastern Mediterranean studies showing the proportions of *Legionella* as the causative agent to CAP; Figure S6: Forest plot of African studies showing the proportions of *Legionella* as the causative agent to CAP; Figure S7: Forest plot of Western Pacific studies showing the proportions of *Legionella* as the causative agent to CAP; Figure S8: Forest plot of South-East Asian studies showing the proportions of *Legionella* as the causative agent to CAP; Figure S9: Forest plot of The Americas studies showing the proportions of *Legionella* as the causative agent to CAP; Figure S10: Baujat plot; Figure S11: Funnel plot to evaluate publication bias (all studies) and Figure S12: Funnel plot (trim and fill).
