# Supplementary material for: Global Perspective of Legionella Infection in Community-Acquired Pneumonia: A Systematic Review and Meta-Analysis of Observational Studies
Source: Int J Environ Res Public Health. 2022 Feb 8;19(3):1907. doi: 10.3390/ijerph19031907 (PMC8835084; doi:10.3390/ijerph19031907)
Supplement: Supplementary file 1 [file ijerph-19-01907-s001.zip › ijerph-1427564-supplementary (1)/ijerph-1427564-supplementary (1).pdf]

Supplementary materials

Supplement to: Graham FF, etc., et al. Global perspective of *Legionella* infection in Community Acquired Pneumonia: A Systematic Review and Meta-analysis of Observational Studies

Supplementary Materials

Graham FF et al.

GLOBAL PERSPECTIVE OF *LEGIONELLA* INFECTION IN COMMUNITY ACQUIRED PNEUMONIA: A SYSTEMATIC REVIEW AND META-ANALYSIS OF OBSERVATIONAL STUDIES

TABLE OF CONTENTS

		Page
Figure S1.	The PRISMA checklist	3
Figure S2.	Search strategy for community acquired pneumonia and *Legionella*	4
Table S1.	Study characteristics summary included in the meta-analysis	6
Table S2.	Studies of patient recruitment from 1 January1990 defining the aetiology of community-acquired pneumonia (CAP) and *Legionella* according to WHO region	7-23
Table S3.	Patient and study characteristics of included studies where *Legionella* was not detected as the cause of infection	24
Table S4.	Patient and study characteristics of excluded studies from the meta-analysis that reported data on overlapping participant populations	25-26
Figure S3.	*Legionella* incidence of CAP studies which recruited patients from 1 January 1990	27
Figure S4.	Forest plot of European studies showing proportions of *Legionella* as the causative agent to CAP	28
Figure S5.	Forest plot of Eastern Mediterranean studies showing proportions of *Legionella* as the causative agent to CAP	29
Figure S6.	Forest plot of African studies showing proportions of *Legionella* as the causative agent to CAP	29
Figure S7.	Forest plot of Western Pacific studies showing proportions of *Legionella* as the causative agent to CAP	30
Figure S8.	Forest plot of South-East Asian studies showing proportions of *Legionella* as the causative agent to CAP	30
Figure S9.	Forest plot of The Americas studies showing proportions of *Legionella* as the causative agent to CAP	31
Figure S10.	Baujat plot	31
Figure S11.	Funnel plot to evaluate publication bias (all studies)	32
Figure S12.	Funnel plot (Trim and Fill)	32
	Complete references for Table S1 studies	34-45

1. Title - The report is identified as a systematic review and meta-analysis

2. Structure summary - The structured abstract includes objectives, data sources, study selection, data extraction, data synthesis and conclusions.

3. Rationale - Described in the Introduction

4. Objectives - Stated in the Introduction

5. Protocol and Registration – The protocol is described in the Methods and in Figure S2 and confirms to the Preferred Reporting Items for Systematic Reviews and Meta-analyses (PRISMA-P2015) guidelines. Registration does not apply

6. Eligibility criteria – They are defined in the Methods

7. Information sources – Described in the Methods

8. Search – Described in the Methods and Figure S2

9. Study selection - Described in the Methods

10. Data collection process – Described in the Methods

11. Data items – Described in Methods and summarized in Table S1

12. Risk of bias in individual studies –We explored heterogeneity with a variety of methods (Methods and Table 1), including quality of study. Randomization does not apply given the prospective nature of the studies

13. Summary measures – 95% Confidence Intervals

14. Planned methods of analysis – Described in Statistical Analysis

15. Risk of bias across studies – Described in Statistical Analysis and reported in detail in Results

16. Additional analyses – Sensitivity analysis, sub-group analysis (Table 1)

17. Study selection – see Flow diagram in Figure 1

18. Study characteristics – shown in Supplementary Table S1

19. Risk of bias within studies – see 12

20. Results of individual studies – shown in Forest plots, map and Table S1

21. Syntheses of results – shown in Results and in Forest plots captions (S3-S8)

22. Risk of bias across studies – see 15

23. Additional analyses – results reported in Table 2

24. Summary of evidence – stated early in Discussion

25. Limitations – addressed in Discussion

26. Conclusion – addressed in the last paragraphs of manuscript

27. Funding – not applicable in this instance

Figure S1. The PRIMSA checklist†

† Liberati A, Altman DG, Tetzlaff J, et al. The PRISMA statement for reporting systematic reviews and meta-analyses of studies that evaluate healthcare interventions: explanation and elaboration. *Br Med J* 2009; 339: b2700

Database: Ovid MEDLINE(R) In-Process & Other Non-Indexed Citations and Ovid MEDLINE(R) <1946 to Present>, adapted for Embase, Scopus, Cochrane Library

Search Strategy:

----

1 exp legionella/

2 legionell*.ab,ti.

3 legionnaire*.ab,ti.

4 legionellosis/

5 legionnaire's disease/

6 Legionella pneumophila/

7 ("l. pneumophilia" or "l pneumophilia" or "l.pneumophilia").ab,ti.

8 1 or 2 or 3 or 4 or 5 or 6 or 7

9 exp Community-Acquired Infections/ and exp Pneumonia/

10 community acquired pneumonia.ab,ti.

11 9 or 10

12 8 and 11

Figure S2. Search strategy for Community-acquired pneumonia and *Legionella*

Search results

The search strategy retrieved 2778 unique citations for the incidence studies of *Legionella* spp. as aetiologies in CAP; 693 were identified from MEDLINE, 693 from EMBASE, 1305 from Scopus, 31 from LILACS and 44 from Cochrane. Of these 1537 were excluded based on duplicates and after the first screening using titles and abstracts, leaving 803 to be examined (Figure 1). After an initial review of titles and abstracts, 402 articles were read in detail after which 151 were excluded for reasons listed in Figure 1. Studies which provided data to calculate the incidence of *Legionella* infection CAP were conducted in each of the six WHO geographic regions, with 116 studies from Europe, 38 studies from Western Pacific, 32 studies from The Americas and 15 studies from Eastern Mediterranean. The reminder of the WHO regions namely South East Asia consisted of 12 studies and Africa 5 studies (Table S1). One international, multicentre study used the Global Initiative for Methicillin-resistant *Staphylococcus aureus* Pneumonia (GLIMP) database to derive data on patients hospitalized with CAP from 54 countries which represented each of the six WHO regions [220]. Eight prospective studies did not detect *Legionella* infection as the cause of CAP and were not included in the meta-analysis (Table S3). Due to overlapping study populations (e.g. 26 studies in Barcelona) or where studies described a secondary analysis of the same cohort of hospitalized patients with pneumonia (Table S4), 219 of the 270 studies were included in our meta-analysis.

Study Characteristics

The studies presenting aetiologies (*Legionella* spp.) included a total of 5723 paediatric and/or adult patients (≥18yrs) (Table S2) out of a total of 125764 patients identified with radiographic evidence of pneumonia. The patient population consisted of inpatients in 178 studies (18 were specific to ICU admitted patients only) and outpatients in 13 studies. Both outpatients and hospitalized (inpatients) with clinical and radiological diagnosis of CAP were recruited in 28 studies (Table S1). Most of the studies focused on mixed populations where the participants' aged was ≥ 13 years (68·0%) or considered all ages (25·9%), and few studies considered only the elderly ≥ 65 years of age (5·2%) and children ≤18 years (0·9%). Data collection spanned over three decades (1990-2018) with just under half the 219 studies meeting our inclusion criteria for meta-analysis published between 2000 and 2009 (45·6%) (Table 1). Of these studies 190 were published in English. The median quality score of all 219 studies was 6 (range 3–9).

Table S1. Study characteristics summary included in the meta-analysis (n=219)

Characteristics	Frequency No. (%)
**Size, No. of Patients (met the CAP criteria)**	
≤ 100	42 (19·2)
101–299	91 (41·5)
300–499	33 (15·1)
500–999	24 (11·0)
≥1000	29 (13·2)
**Year of publication**	
1990–1999	40 (18·3)
2000–2009	100 (45·6)
2010–2018	79 (36·1)
**WHO region**	
Europe	116 (59·2)
Eastern Mediterranean	15 (6·8)
Africa	5 (2·3)
Western Pacific	38 (17·4)
South–East Asian	12 (5·5)
The Americas	32 (14·6)
All	1 (0·5)
**Language**	
English	190 (86·8)
**Design**	
Prospective cohort	173 (79·0)
Retrospective cohort	33 (15·1)
Cross-sectional	7 (3·2)
Case-control	6 (2·7)
**Healthcare Setting**	
Inpatients not admitted to the ICU	160 (74·0)
ICU admitted patients	18 (8·4)
Outpatients with no comorbidities	8 (3·1)
Outpatients with cardiopulmonary disease or other modifying factors	5 (1·9)
Both inpatients (including ICU) and outpatients	28 (12·6)
**NOQAS (Newcastle-Ottawa Quality Assessment Scale)**	
Poor (score 0–3)	25 (11·4)
Moderate (score 4–6)	126 (57·5)
High (score 7–9)	68 (31·1)

Table S2. Patient and study characteristics of included studies for the meta-analysis

No.	Study Reference	Study Characteristics								CAP Results			*Legionella* Results		
	First Author, Year, Ref	Study Design	WHO Region	City or Region/Country	Study period (for patient recruitment)	Quality Assessment (NOS)	Age group (median or mean (±SD)	Male CAP patients (%) [no.]	*Legionella* Patients Identified by Diagnostic Method No.	Estimated (~) Study Population (Year)	No. of patients with CAP (that meet the criteria)	CAP incidence (annual)	Aetiology of CAP, (%) [no.] of patients<br>*Legionella* spp.<br>Lp = *L. pneumophila*	*Legionella* incidence (annual) of CAP	Case fatality risk *Legionella* spp. No. of patients who died/total no. of patients (%)
1	Aliberti 2011[1]	Prospective (observational) study	European	Multinational (Milan/Italy; Edinburgh/Scotland; Barcelona/Spain)	Apr 2008-Apr 2010	9	≥18yrs (median 73yrs; range (56–82yrs)	46·8 [1005]	NR*	Milan ~1,309,000 Edinburgh ~458,000 (2005) Barcelona ~1,593,000 (2005)	2145	31·9	1.7 [37] Lp	0·6	NDR
2	Almirall 1999[2]	Case-control study	European	Maresme (Barcelona)/Spain	Dec 1993-Nov 1995	8	≥14 yrs	54·6 [112]	5 serology; 1 UAT	74 610 (published)	205	137·4	2·4 [5] Lp	3·3	NDR
3	Almirall 1993[3]	Prospective hospital study	European	Maresme (Barcelona)/Spain	Apr 1990-Mar 1991	3	≥13 yrs (range 13-101 yrs)	65·7 [69]	3 serology	39,733 (published)	105	264·3	2·9 [3] Lp	7·6	0
4	Álvarez 2006[4]	Prospective study	European	Santa Cruz de Tenerife/Spain	Jan 1996-Feb 2001	5	All ages	¤NR	6 serology; 2 UAT	~215,132 (2000)	390	30·2	2·1 [8] NB specific *Legionella* spp. not recorded	0·6	NDR
5	Alvarez-Lerma 2004[5]	Prospective (observational) study	European	Multicentre/Spain	Oct 2000-Nov 2001	7	All ages	¤NR	NR*	~40,200,000 (1999)	220	0·5	10·5 [23] Lp	0·1	NDR
6	Alvarez-Sanchez 1998[6] (Spanish)	Prospective hospital study	European	Multicentre/Spain	Nov 1991- Oct 1992	7	All ages	72·5 [190]	NR*	38,883,000 (1990)	262	0·7	8·0 [21] Lp	0·1	7/21 (33·3)
7	Andreo 2006[7]	Prospective hospital study	European	Hospital Universitari Germans Trias i Pujol, Barcelona/Spain	Feb 2000-Jun 2001	6	≥ 16 yrs (58·6 ±19·4)	70·1 [75]	1 serology; 5 UAT	1,357,430 (≥ 14 yrs) (2000)	107	7·9	5·6 [6] Lp	0·4	NDR
8	Antela 1993[8] (Spanish)	Prospective hospital study	European	Madrid/Spain	Apr 1990-Mar 1991	3	≥14 yrs (mean 50·6; range 14-88 yrs)	66·3 [67]	NR* culture, serology	Total = 4,947,555 (1991); 0-14yrs = 937742 (1991)	101	2·0	3·0 [3] Lp	0·1	NDR
9	Beovic´ 2003[9]	Prospective hospital study	European	Multicentre/Slovenia	Nov 1999-Apr 2001	4·5	≥15 yrs	61·9 [70]	2 serology	1,989,000 (NB 0-14yrs = 316,251) (2000)	113	3·4	1·8 [2] Lp	0·1	0
10	Bjarnason 2018[10]	Prospective hospital study	European	Reykjavik/Iceland	Dec 2008-Nov 2009	9	≥ 18yrs	49·7 [154]	NR* UAT; culture; PCR	~243,340 (2005); ≤15yrs	310	127·4	1·0 [3] Lp	1·2	0
11	Blasi 1995[11]	Prospective hospital study	European	Milan (Ospedale Maggiore di Milano)/Italy	Nov 1991-Dec 1993	3	NAR¤ (mean 42·1yrs; range 17-94 yrs)	51·7 [107]	10 serology	1,369,231 (1991)	207	7·6	4·8 [10] Lp	0·4	NDR
12	Bochud 2001[12]	Prospective hospital study	European	Multicentre/Switzerland	4-yr period (actual yrs not stated)	7	≥15yrs (age range, 15–96yrs; median, 43yrs)	48·2 [82]	1 serology	7,144,000 (1999)	170	2·4	0·6 [1] Lp	0·01	0
13	Bodi 2005[13]	Prospective study	European	Multicentre/Spain	Dec 2000-Feb 2002	7	All ages (59·9 ±16·1)	71·8 [380]	NR* UAT; serology; culture	~40,200,000 (1999)	529	1·1	4·3 [23] NB specific *Legionella* spp. not recorded	0·04	NDR
14	Boersma 2005[14]	Cross-sectional study	European	Groningen/The Netherlands	1993-1994	6	All ages median 50; range 29–70)	60·3 [47]	NR* serology; culture	558,000 (1990)	78	14·0	12·8 [10] Lp	1·8	NDR
15	Bohte 1995[15]	Prospective hospital study	European	Leiden/The Netherlands	Jan 1991-Apr 1993	5	≥18yrs	58·4 [195]	8 serology	110,423 (1990); ~20,000 ≤18yrs) (1990)	334	184·7	2·4 [8] Lp	4·4	NDR
16	Botelho-Nevers 2016[16]	Prospective study	European	Saint-Etienne/France	Mar 2007-Apr 2010	7	≥18yrs (61·3 ±18)	61·4 [156]	14 PCR, 7 culture, 8	≥15yrs 128,532	254	65·9	9·4 [24] Lp	6·2	NDR

No.	Study Reference	Study Characteristics									CAP Results		*Legionella* Results		
	First Author, Year, Ref	Study Design	WHO Region	City or Region/Country	Study period (for patient recruitment)	Quality Assessment (NOS)	Age group (median or mean (±SD)	Male CAP patients (%) [no.]	*Legionella* Patients Identified by Diagnostic Method No.	Estimated (~) Study Population (Year)	No. of patients with CAP (that meet the criteria)	CAP incidence (annual)	Aetiology of CAP, (%) [no.] of patients<br>*Legionella* spp.<br>Lp = *L. pneumophila*	*Legionella* incidence (annual) of CAP	Case fatality risk *Legionella* spp. No. of patients who died/total no. of patients (%)
									4serology, 15 UAT	(2007)					
17	Braun 2004[17] (Dutch)	Prospective observational study	European	Schiedam;Vlaardingen/The Netherlands	Jan 1998-Dec 1999	5	All ages median 62; range 20–89)	56·1 [88]	NR* serology, culture, UAT	Schiedam 73,480 (1995) Vlaardingen 73, 719 (1995)	157	53·3	7·6 [12] Lp	4·1	NDR
18	Bruns 2009[18]	Prospective (cohort) study	European	Amersfoort and Groningen/The Netherlands	Jul 2000-Jun 2003	6	≥18yrs	65·1 [108]	NR* UAT; serology; culture	~126,065 Amersfoort; ~19,360 Groningen (2001 census)	166	38·0	4·2 [7] Lp	1·6	NDR
19	Cabre, 2010[19]	Prospective (cohort) study	European	Mataró, Spain	Jan 2001-Aug 2005	8	≥70yrs (84.51 ±6·80)	59·7 [80]	NR*	~116,698 (2005)	134	114·8	1·5 [2] Lp	1.7	NDR
20	Capelastegui 2012[20]	Prospective hospital study	European	Basque country/Spain	Apr 2006-Jun 2007	8	≥18yrs	¤NR (for all CAP patients)	17 UAT 0 serology 0 culture	254,523 (published)	700	275·0	2·8 [11] Lp (inpatients) 1·5 [6] Lp (outpatients)	6·7	NDR
21	Chalmers 2011[21]	Prospective observational hospital study	European	Edinburgh/Scotland	Jan 2005-May 2009	9	NAR¤ (median CAP 65yrs; HCAP 76yrs)	48·9 [659]	NR*culture, UAT	~457,830 (2005)	1348 [1071 CAP; 277 HCAP]	294·4	4·4 [59] Lp [50 CAP; 9 HCAP]	12·9	NDR
22	Cillóniz 2017[22]	Prospective observational study	European	Hospital Clinic of Barcelona/Spain	Jan 2003-Dec 2014	6	All ages (66 ±19·5)	60·7 [2689]	Culture, PCR, UAT	1,611,013 (2011 census)	4431	19·6	2·2 [97] Lp	0·4	NDR
23	Deschamps 2004[23] (French)	Retrospective study	European	Paris/France	May 2000-Oct 2001	5	≥16yrs	64·6 [95]	1 UAT; NR* serology; culture	~1,822,641 (1999 census; ≥15yrs)	147	2·7	1·4 [2] Lp	0·04	NDR
24	Diederen 2008[24]	Retrospective study	European	Tilburg/The Netherlands	Dec 2002-Nov 2005	6	All ages	¤NR	40 PCR	202,091 (2008)	151	24·9	26·5 [40] 36 Lp; 4 non- *pneumophila Legionella* spp.	6·7	NDR
25	Falcone 2015[25]	Prospective observational study	European	Rome/Italy	Jan 2011-Jan 2013	9	≥18yrs	¤NR for CAP cases only	*NR serology, culture, UAT	≥15yrs ~2,130,375 (2011)	536	12·6	1·9 [10] Lp	0·3	NDR
26	Falguera 2010[26]	Prospective randomised study	European	Catalonia/Spain	Apr 2006- Mar 2008	7	≥18yrs	66·1 [117]	3 UAT, 0 serology, 0 culture	7,364,000 (2008)	177	1·2	1·7 [3] Lp	0·02	NDR
27	Falguera 2005[27]	Prospective hospital study	European	Lleida/Spain	Jan 1998-Dec 2002	8	NAR¤ (mean 69 yrs with diabetes; 54 yrs without diabetes)	64·4 [425]	NR* culture, serology, UAT	113,040 (2001)	660	116·8	4·1 [27] Lp	4·8	NDR
28	Fernández 1999[28] (Spanish)	Prospective study	European	Hospital Universitari Germans Trias i Pujol, Barcelona/Spain	Jan 1995-Jun 1997	6	≥15yrs (median 63)	¤NR	6 UAT; 0 culture	≥15yrs 1,212,131 (1991)	69	2·3	8·7 [6] Lp	0·2	NDR
29	Fiumefreddo 2009[29]	Retrospective hospital study	European	Basel/Switzerland	1997-2007	9	NAR¤ (median 72 yrs) (68 (49-77) *Legionella* CAP))	62·0 [279]	72 UAT; 54 culture; 38 PCR	166,558 (2000)	450	24·6	18·2 [82] Lp (70) *Legionella* spp. 12	4·5	NDR
30	Fraisse 2012[30]	Retrospective study	European	Bagnols-sur-Cèze/France	Jan 2008-Jul 2009	5	≥16yrs	¤NR	1 UAT	15,263 (2007 census; ≥15yrs)	292	497·9	0·3 [1] Lp	6·6	NDR

No.	Study Reference	Study Characteristics								CAP Results			Legionella Results		
	**First Author, Year, Ref**	**Study Design**	**WHO Region**	**City or Region/Country**	**Study period (for patient recruitment)**	**Quality Assessment (NOS)**	**Age group (median or mean (±SD)**	**Male CAP patients (%) [no.]**	***Legionella* Patients Identified by Diagnostic Method No.**	**Estimated (~) Study Population (Year)**	**No. of patients with CAP (that meet the criteria)**	**CAP incidence (annual)**	**Aetiology of CAP, (%) [no.] of patients *Legionella* spp. Lp = *L. pneumophila***	***Legionella* incidence (annual) of CAP**	**Case fatality risk *Legionella* spp. No. of patients who died/total no. of patients (%)**
31	Garbino 2002[31]	Prospective (randomised) hospital study	European	Multicentre/Switzerland	Nov 1997-May 1999	5	≥18yrs (70·4±16·2)	59·1 [188]	7 serology; 6 UAT; 1 culture; 3 PCR	7,090,000 (1997)	318	2·8	5·3 [17] Lp	0·1	NDR
32	Garcia Vazquez 2005[32]	Prospective (cohort) hospital study	European	Hospital Mutua de Terrassa, Barcelona/Spain	Oct 1996-Dec 2001	8	≥14yrs	¤NR	NR* culture, serology, UAT	~1,329,257 (1996; ≥15yrs)	1391	17·7	3·9 [54] Lp	0·7	NDR
33	Garcia-Vidal 2007[33]	Retrospective (cohort) hospital study	European	Hospital Mutua de Terrassa,Barcelona/Spain	Oct 2001- Dec 2003	8	NAR¤ (adults only)	68·5 [211]	UAT	~300 000 (published)	308	46·7	2·6 [8] Lp	1·2	1/8 (12·5)
34	Gattarello 2015[34]	Case-control study	European	Multicentre/Spain	2000-2002<br>2008-2014	9	All ages	68·1 [49]<br>75·0 [54]	NR* culture, UAT	~ 46,770,000 (2014)	72<br>72	0·1<br>0·03	20·8 [15] Lp<br>20·8 [15] Lp	0·01<br>0·01	3/15 (20)<br>5/15 (33·3)
35	Georges 2013[35]	Retrospective study	European	Tourcoing/France	Jan 1995-Dec 2000;<br>Jan 2005-2010	6	≥18yrs	70·3 [223]	NR* culture; serology; UAT	~70,700 (1999 census; ≥15yrs)	317	40·8	2·5 [8] Lp	1·0	NDR
36	Gómez 1996[36]	Prospective hospital study	European	Murcia/Spain	Jan 1991-Apr 1994	7	NAR¤ (58 ±10; range 14-90)	67·0 [67]	NR* serology, culture	349,040 (1991)	100	8·4	5 [5] Lp	0·4	1/5 (20)
37	Gutiérrez 2005[37]	Prospective hospital study	European	Bajo Vinalopo/Spain	Oct 1999-Oct 2001	9	≥15yrs (CAP mean (range), 56·6 yrs (15–94 yrs); CAP dist by age grp 15–44 yrs 161 (32·7), 45–64 yrs 109 (22·1), 65–74yrs 87 (17·6), + 75 yrs 136 (27·6)	62·5 [308]	27 serology; 15 UAT	239, 335 (published)	493	102·9	4·3 [21] Lp	4·4	NDR
38	Gutiérrez 2001[38]	Prospective hospital study	European	Seville/Spain	1996-1999	6	All ages (43±18)	60·6 [134]	17 serology	701,927 (1991)	221	7·9	7·7 [17] Lp	0·6	NDR
39	Herrera-Lara 2013[39]	Prospective/longitudinal hospital study	European	Valencia/Spain	Jan 2006-Dec 2009	5	All ages (63·92±17·4; range, 14-90)	64·6 [157]	NR* serology, culture, UAT	739,412 (1991)	243	8·2	8·6 [21] Lp	0·7	NDR
40	Hohenthal 2004[40]	Retrospective hospital study	European	Turku/Finland	1996 – 2000	5	All ages (59 ± 13·5; range 29- 82)	67·6 [48]	2 serology; 1 PCR	200,000 (published)	71	7·1	4·2 [3] Lp	0·3	1/3 (0·3)
41	Hoogewerf 2006[41]	Prospective randomised trial	European	Multicentre/The Netherlands	Jul 2000-Jun 2003	8	NAR¤ (av 68·5yrs)	69·2 [180]	NR* serology, culture, UAT	15,925,513 (2000)	260	0·5	3·8 [10] Lp	0·03	NDR
42	Hopstaken 2004[42]	Prospective hospital study	European	Maastricht/The Netherlands	Jan 1998-Apr 1999	6	NAR¤ (mean 52yrs (range 18-89))	47·3 [115]	NR*	117,008 (1990)	243	148·3	0·4 [1] Lp	2·9	NDR
43	Holter 2015[43]	Prospective hospital study	European	Drammen/Norway	Jan 2008-Jan 2011	7	≥18yrs	52·4 [140]	7 UAT, 0 culture, 0 PCR	160,000 (published)	267	55·6	2·6 [7] Lp	1·5	NDR
44	Hraiech 2013[44]	Prospective (cohort) study	European	Marseille/France	Jan 2007-Dec 2011	7	≥18yrs (57 ± 17)	69·0 [69]	NR*	~697,692 (2007 census; ≥15yrs)	100	2·9	6·0 [6] Lp	0·2	NDR
45	Hug 2001[45]	Retrospective hospital study	European	Multicentre/Switzerland	Jan-Dec 1998	6	≥18yrs (median 68; range 18–97)	60·0 [201]	NR* serology	7,017,000 (1995) NB 0-14yrs = 1,238,000 (1995)	335	5·8	2·1 [7] Lp	0·1	NDR

No.	Study Reference			Study Characteristics							CAP Results			*Legionella* Results	
	**First Author, Year, Ref**	**Study Design**	**WHO Region**	**City or Region/Country**	**Study period (for patient recruitment)**	**Quality Assessment (NOS)**	**Age group (median or mean (±SD)**	**Male CAP patients (%) [no.]**	***Legionella* Patients Identified by Diagnostic Method No.**	**Estimated (~) Study Population (Year)**	**No. of patients with CAP (that meet the criteria)**	**CAP incidence (annual)**	**Aetiology of CAP, (%) [no.] of patients *Legionella* spp. Lp = *L. pneumophila***	***Legionella* incidence (annual) of CAP**	**Case fatality risk *Legionella* spp. No. of patients who died/total no. of patients (%)**
46	Huijskens 2013[46]	Prospective hospital study	European	Tilburg/The Netherlands	Apr 2008-Mar 2009	6	≥18yrs (mean 65yrs; median 68yrs, range 20-94yrs))	61·3 [250]	9 UAT; 3 serology; 8 PCR	202,091 (2008)	408	201·9	3·7 [15] Lp	7·4	NDR
47	Huijts 2013[47]	Prospective (cohort) hospital study	European	The Netherlands	Jan 2008-Apr 2009	6	≥18yrs (median 69yrs (57-79yrs)	62·7 [687]	25 UAT	≥20yrs 14,606,062 (2008)	1095	129·0	2·3 [25] Lp	2·9	NDR
48	Johansson 2010[48]	Prospective hospital study	European	Stockholm/Sweden	2004-2005	6	≥18yrs (mean 61·3; range 18–93)	51·1 [94]	2 UAT; 1 culture and/or PCR	Total = 1,872,184 (2004) NB ≤18yrs = 408,459 (2004)[1]	184	6·3	1·6 [3] Lp	0·1	NDR
49	Jover 2008[49]	Retrospective hospital study	European	Alicante/Spain	Jan 1995-Feb 2003	7	≥18yrs (mean 63 yrs; range 27–93 yrs)	64·6 [53]	1 UAT	~1,463,000 (2000)	82	5.6	1·2 [1] Lp	0.01	NDR
50	Klapdor 2012[50]	Prospective hospital study	European	Multicentre/Germany (CAPNETZ)	Jan 2002-Jul 2007	6	≥18yrs (60·9 ± 18·5; range 18-101 yrs)	44·4 [3464]	UAT, culture, serology, PCR	~82,689,000 (2005)	7803	9·4	4·1 [319] Lp 4·7 ≤64 yrs; 4·4 ≥ 65 yrs	0·4	NDR
51	Kofoed 2007[51]	Prospective (cohort) hospital study	European	Copenhagen/Denmark	Feb 2005-Feb 2006	7	≥18yrs (56yrs (20–94))	48·3 [73]	NR	~134,275 (2005)	151	112·5	2·6 [4] Lp	3·0	NDR
52	Konstantinou 1999[52] (Greek)	Prospective study	European	Thessaloniki/Greece	1992-1996	3	All ages	¤NR	12 serology	752,963 (1991 census)	1013	33·6	1·2 [12] Lp	0·4	NDR
53	Kurutepe 2012[53]	Prospective study	European	Manisa/Turkey	Nov 2008-Nov 2010	5	≥18yrs (mean 58; range 19-81)	73·4 [94]	0 culture; 2 serology; 1 PCR	~192,614 (2000 census; ≥15yrs)	128	33·2	1·6 [2] Lp	0·5	NDR
54	Laurichesse 2001[54]	Prospective hospital study	European	Multicentre/France	Nov 1998-Apr 1999	7	≥18yrs (66·7±20; range 16–100)	53·0 [114]	NR*culture, serology UAT	Total Pop 59,750,000 (1996); Pop ≤ 15yrs = 11,245,500 (1996)	215	0·4	2·3 [5] Lp	0·01	NDR
55	Le Moing 2006[55]	Prospective (cohort) hospital study	European	France	May 1997-Jun 1999	5·5	All ages (median 36yrs)	78·0 [999]	1 serology	58, 375, 000 (1996)	1281	1·1	0·1 [1] Lp	0·001	NDR
56	Lim 2001[56]	Prospective hospital study	European	Nottingham/England	Oct 1998-Oct 1999	7	≥16yrs; 18-44yrs (49); 45-64yrs (59); 75-84yrs (68); 85+ yrs(44)	50·1 [135]	8 serology; 7 UAT	700 000 (published)	267	38·1	3·4 [9] Lp	1·3	1/9 (11·1)
57	Logroscino 1999[57]	Prospective hospital study	European	Multicentre/Italy	Oct 1994-Feb 1996	5	≥14yrs (1 Lp <20yrs; 2 Lp 21-30yrs; 2 Lp 31-40 yrs; 3 Lp 41-50 yrs; 3 Lp 51-60 yrs; 5 Lp 61-70 yrs; 1 Lp 72-80 yrs)	67·9 [416]	NR* culture, serology	≥15yrs ~48,028,031 (1991)	613	0·9	2·8 [17] Lp	0·03	0
58	Lorente 2000[58]	Prospective hospital study	European	Catalonia/Spain	Sep 1996-Mar 1998	5	NAR¤ (adult patients)	60·5 [69]	2 serology	6,059,494 (1991)	114	1·2	1·8 [2] Lp	0·02	NDR

67[1] www.statistikdatabasen.scb.se/

No.	Study Reference	Study Characteristics								CAP Results			*Legionella* Results		
	First Author, Year, Ref	Study Design	WHO Region	City or Region/Country	Study period (for patient recruitment)	Quality Assessment (NOS)	Age group (median or mean (±SD)	Male CAP patients (%) [no.]	*Legionella* Patients Identified by Diagnostic Method No.	Estimated (~) Study Population (Year)	No. of patients with CAP (that meet the criteria)	CAP incidence (annual)	Aetiology of CAP, (%) [no.] of patients *Legionella* spp. Lp = *L. pneumophila*	*Legionella* incidence (annual) of CAP	Case fatality risk *Legionella* spp. No. of patients who died/total no. of patients (%)
59	Madeddu 2008[59]	Retrospective study	European	Sardinia/Italy	Jan 1999-Dec 2004	7	NAR¤ (adult patients)	¤NR	1 UAT	~1,632000 (2001)	84	5·1	1·2 [1] Lp	0·7	NDR
60	Maltezou 2004[60]	Prospective hospital study	European	Athens/Greece	Jan-Dec 2001	5	6 months-14yrs (mean 6yrs (range 10months-13yrs))	¤NR	1 serology	0-14yrs ~ 432,670 (2001)	65	15·0	1·5 [1] Lp	0·2	NDR
61	Marcos 2003[61]	Prospective hospital study	European	Hospital Clinic of Barcelona/Spain	Jan 2001-Apr 2001	6	≥18yrs (mean 50; range 24-95)	78·9 [314]	NR* culture, serology, UAT	≥19yrs 3,889,692 (2001)	398	10·2	4·3 [17] Lp	0·4	NDR
62	Martinez 2009[62]	Retrospective study	European	Multicentre/Spain	Jan-Mar 2003	5	≥14yrs (67·0 ± 24·6; range 15-94)	65·4 [223]	NR* culture; UAT	28,142,817 (2001 census; ≥15yrs)	341	1·2	2·6 [9] Lp	0·03	NDR
63	Martínez-Moragón 2004[63]	Prospective study	European	Port Sagunt, Valencia /Spain	Jan-Jul 2003	5	≥65yrs	44·0 [40]	2 UAT; culture; serology	130,000 (published)	91	70·0	2·2 [2] Lp	1·5	NDR
64	Maurin 2010[64]	Prospective study	European	Chambery/France	Nov 2004-Mar 2006	6	≥18yrs	¤NR	14 UAT; 8 serology; 9 PCR	~46,157 (1999; ≥15yrs)	255	368·3	12·2 [31] Lp	44·8	NDR
65	Mena-Bernal 2011[65] (Spanish)	Retrospective hospital study	European	Valladolid/Spain	2006	5	≥ 18yrs (69·9±17·8)	64·8 [295]	90 UAT	319,943 (2006)	379	118·5	23·7 [90] Lp	28·1	NDR
66	Menéndez 2012[66]	Prospective study	European	Hospital Universitari I Politècnic La Fe, Valencia; Hospital clinic Barcelona, Barcelona/Spain	Oct 2004-Sep 2005	5·5	All ages	¤NR	NR* UAT; culture; 78serology	1,503,884 (2001)	685	45·5	3·5 [24] Lp	1·6	NDR
67	Menéndez 1999[67]	Prospective hospital study	European	Valencia (University Hospital)/Spain	Jan 1996-Jan 1997	6	All ages (62 ± 19)	62·5 [115]	1 culture	2,117,927 (1991)	184	8·7	0·5 [1] Lp	0·05	NDR
68	Michetti 1995[68]	Prospective hospital study	European	Lombardy/Italy	Mar 1991-Feb 1992	4	All ages (mean inpatients 49·8; outpatients 37·4) yrs (4 Lp 30-39yrs; 4 Lp 40-49yrs; 1 Lp 50-59 yrs; 2 Lp 60-69 yrs)	52·0 [93]	NR* culture, serology	~10,000,000 (1991) (published)	179	1·8	6·1 [11] Lp	0·1	NDR
69	Mirete 2001[69] (Spanish)	Prospective study	European	Alicante/Spain	Oct 1999-Oct 2000	5	All ages	¤NR	2 UAT; 0 culture; 2 serology	250,000 (published)	240	96	5·6 [2] Lp	0·8	NDR
70	Molinos 2009[70]	Prospective observational hospital study	European	Asturias/Spain	Apr 2003-Apr 2004	8	All ages (mean 67·1)	68·0 [483]	NR* serology, culture, UAT	1,073,761 (2004)	710	66·1	6 [40] Lp	3·7	NDR
71	Molinos 1997[71]	Prospective hospital study	European	Oviedo/Spain	Aug 1991-Apr 1994	5	All ages (mean 58; range 16-101)	78·7 [59]	NR* culture, serology, DFA	199,549 (1991)	75	15·04	12 [9] Lp	1·8	NDR
72	Montagna 2006[72]	Prospective hospital study	European	Southern Italy	Jan 2001-Mar 2005**	6	All ages (mean 62·6; range 36-90)	64·5 [645]	Culture, serology, UAT	~14,000,000	1000	1·7	4·7 [47] NB specific *Legionella* spp. not recorded	0·1	NDR
73	Mothes 2016[73]	Retrospective study	European	Multicentre/France	Jan 2010-Dec 2013	7	All ages	¤NR	NR* UAT; culture	~65,030,000 (2010)	861	0·3	20·2 [174] Lp	0·1	NDR

No.	Study Reference	Study Characteristics								CAP Results			*Legionella* Results		
	First Author, Year, Ref	Study Design	WHO Region	City or Region/Country	Study period (for patient recruitment)	Quality Assessment (NOS)	Age group (median or mean (±SD)	Male CAP patients (%) [no.]	*Legionella* Patients Identified by Diagnostic Method No.	Estimated (~) Study Population (Year)	No. of patients with CAP (that meet the criteria)	CAP incidence (annual)	Aetiology of CAP, (%) [no.] of patients *Legionella* spp. Lp = *L. pneumophila*	*Legionella* incidence (annual) of CAP	Case fatality risk *Legionella* spp. No. of patients who died/total no. of patients (%)
74	Neocleous 2014[74]	Retrospective hospital study	European	Central Greece	2007-2009	3	≥ 17yrs (mean 61yrs (18-80yrs)	70·7 [152]	1 UAT, 3 serology, 1 PCR	600,000 (published)	215	11·9	1·4 [3] Lp	0·2	NDR
75	Norrby 1997[75]	Prospective study	European	Multinational (Denmark/Finland/Iceland/ Sweden/ Norway)	May 1990-May 1993	6	≥18yrs	68·3 [99]	11 serology	~4,263,846 Denmark; ~4,024,000 Finland; ~191,218 Iceland; ~7,023,809 Sweden; ~3,438,161 Norway (1990; ≥15yrs)	441	2·3	2·5 [11] 6 Lp; 2 *L. micdadei*; 1 *L. bozemanae*; 1 *L.micdadei* + *L. dumoffi*; 1 Lp + *L. dumoffi*	0·1	NDR
76	Olaechea 1996[76]	Prospective hospital study	European	Multicentre/Spain	Nov 1991-Oct 1992	6	NAR¤ (52·9 ± 9·6)	¤NR	NR* culture, serology	38,939,049 (1991)	262	0·7	8·0 [21] Lp	0·05	NDR
77	Pereira 2013[77]	Prospective (cohort) study	European	Porto/Portugal	Dec 2008-Jan 2013	5	≥18yrs	62·9 [68]	NR	~201,319 (2011 census) (≥19yrs)	108	13·4	4·6 [5] Lp	0·6	NDR
78	Pletz 2014[78]	Cross-sectional study	European	Multicentre/Germany	Jun 2002-Dec 2008	7	≥18yrs (62·5± 16·7; *Legionella* cases only)	57·3 [172]	NR* culture, UAT, PCR	82,075,000 (2000)	300	0·1	16·3 [49] Lp	0·02	NDR
79	Pradelli 2015[79]	Observational (cohort) study	European	Nice/France	Jul 2005-Jun 2014	7	¤NAR (mean ~ 65yrs	¤NR (reported sex ratio)	UAT, culture	600,000 (published)	1370	25·4	7·2 [98] Lp	1·8	1/98 (1·0)
80	Prat 2006[80]	Prospective hospital study	European	Hospital Universitari Germans Trias i Pujol, Barcelona/Spain	2001-2003**	5	¤NAR (56·6 ±19·1)	62·9 [73]	21 UAT	~1,583,000	116	7·3	18·1 [21] Lp	0·4	NDR
81	Putinati 2004[81] (Italian)	Prospective hospital study	European	Ferrara/Italy	Jan 2001-Dec 2003	6	≥65yrs (79 ± 7·9)	56·6 [176]	1 serology; 3 UAT	130,992 (total 1991 census); ~ 96,410 ≥65yrs	311	107·5	1·3 [4] Lp	1·4	NDR
82	Reissig 2013[82]	Prospective study	European	Multinational (Italy/Austria/Germany)	Nov 2007-Feb 2011	5	All ages (median 63·8; range 19-95)	NR for CAP which met the criteria only for those cases who were assessed	67 UAT; 0 culture	~58,941,500 Italy; ~8,267,946 Austria; ~82,376,450 Germany (2006)	229 (222 inpatients; 7 outpatient s	1·2	29·3 [67] Lp	0·4	NDR
83	Rello 2003[83]	Retrospective hospital study	European	Multicentre/Spain	Jan 1993-Jan 2000	6	All ages (median intubated 63, range 22-88; median non-intubated 58 range 19-86)	80·4 [164]	12 UAT, NR* serology, culture	39,189,400 (1993)	204	0·1	11·3 [23] Lp	0·01	5/23 (21·7)
84	Rello 1996[84]	Prospective hospital study	European	Multicentre/Spain	Nov 1991-Oct 1992	3	≥65yrs (72·08±5·04)	73·7 [185]	2 culture; 1 serology	≥65 yrs (1990) (21,574,108); total (38,872,268)	251	0·6	1·2 [3] Lp	0·01	2/3 (66·7)
85	Riquelme 1996[85]	Cohort and case-control study	European	Barcelona/Spain	Nov 1993-Jun 1994	7	≥65yrs (78·5±7·9)	66·3 [67]	3 serology	≥65yrs (287,619); (total 1,643,542) (1991)	101	35·1	3·0 [3] Lp	1·0	NDR

No.	Study Reference	Study Characteristics								CAP Results			*Legionella* Results		
	First Author, Year, Ref	Study Design	WHO Region	City or Region/Country	Study period (for patient recruitment)	Quality Assessment (NOS)	Age group (median or mean (±SD)	Male CAP patients (%) [no.]	*Legionella* Patients Identified by Diagnostic Method No.	Estimated (~) Study Population (Year)	No. of patients with CAP (that meet the criteria)	CAP incidence (annual)	Aetiology of CAP, (%) [no.] of patients *Legionella* spp. Lp = *L. pneumophila*	*Legionella* incidence (annual) of CAP	Case fatality risk *Legionella* spp. No. of patients who died/total no. of patients (%)
86	Rizzato 1995[86]	Prospective hospital study	European	Milan (University)/Italy	Oct 1992-Aug 1993	5	≤ 75yrs	72·5 [29]	5 serology	1,369,295[2] (NB 18·2% ≥65yrs) = 249,212) (1991)	40	3·6	12·5 [5] Lp	0·4	NDR
87	Roed 2015[87]	Cohort study	European	Aalborg/Denmark	Jan 2003-Dec 2013	4	All ages (mean 58·8)	60·0 [60]	25 UAT	580,000 (published)	100	1·6	25 [25] Lp	0·4	NDR
88	Rosón 2000[88] Rosón 2001 (fatality data)[89]	Prospective hospital study	European	Hospital Clinic of Barcelona/Spain	Feb 1995-May 1997	7	All ages (mean 64; range 16–96)	69·6 [371]	NR* culture, serology, UAT	1 100 000 (published)	533	21·1	6·6 [35] Lp	1·4	5/35 (14·3)
89	Rovira 1999[90]	Prospective study	European	Valencia (Hospital de Segunt)/Spain	Jan 1996-Jun 1997	5	≥15yrs (38±15yrs)	58·9 [53]	NR* serology; culture	~713,609 (1991 census; ≥15yrs)	90	8·4	7·8 [7] Lp	0·7	NDR
90	Røysted 2016[91]	Prospective hospital study	European	Telemark/Østfold/Norway	May 2007-Dec 2008	5	≥18yrs (65±18)	44·4 [166]	9 serology; 8 UAT; 0 culture	400,000 (published)	374	62·3	4·5 [17] Lp	1·2	3/17 (0·2)
91	Ruiz 1999[92]	Prospective hospital study	European	Barcelona/Spain	Oct 1996-Sep 1998	7	All ages (65±14; range 16-86)	73·6 [67]	NR* culture, serology, UAT	1,505,581 (1991)	91	3·0	2·2 [2] Lp	0·1	NDR
92	Sahuquillo-Arce 2016[93]	Prospective hospital study	European	Multicentre/Spain	Nov 2005-Oct 2007**	6	All ages	65·2 [2807]	NR* culture, serology, UAT	~ 45,200,000 (2007)	4304	4·8	1·2 [50] Lp	0·1	NDR
93	Sangil 2012[94]	Prospective hospital study	European	Hospital Universitari Mutua Terrassa Barcelona/Spain	Nov 2009-Oct 2010	5	≥ 18yrs	68·7 [90]	1 UAT, PCR, serology, culture	350,000 (published)	131	37·4	0·8 [1] Lp	0·3	NDR
94	Santos 2003[95]	Prospective study	European	Multicentre/Spain	Oct 1998-Jun 1999	3	All ages	¤NR	28 serology	~39,670,000 (1996)	241	0·6	11·6 [28] Lp	0·1	NDR
95	Santos de Unamuno 1998[96] (Spanish)	Prospective hospital study	European	Palma de Mallorca/Spain	Nov 1992-Dec 1994	4	≥14yrs	49·5 [45]	NR* culture, serology	60,450 (published)	91	75·3	3·3 [3] Lp	2·5	NDR
96	Sayan 2003[97] (Turkish)	Case-control study	European	Izmir/Turkey	2000-2001	4	All ages	67·9 [36]	18 serology	2,232,265 (2000)	53	1·2<br>0·5	28·3 [15] Lp Patient group<br>15 [3] Lp Control group	0·4<br>0·05	NDR
97	Schneeberger 2004[98]	Prospective hospital study	European	Noord Brabant, Utrecht/The Netherlands	Sep 1997-May 1999	6	All ages (median 55; range 1-88; 12% < 13 yrs)	57·0 [91]	3 culture; 1 PCR	Tilburg (156,421); Bilthoven (32,174); Den Bosch (91,113) (1990)	159	35·5	2·5 [4] Lp	0·9	NDR
98	Sever 2013[99] (Turkish)	Prospective hospital study	European	Izmir/Turkey	Dec 2001-Dec 2002	6	≥18yrs	¤NR	1 UAT	1,674,199 ≥19yrs (2000)	72	4·3	1·4 [1] Lp	0·1	NDR
99	Sicras-Mainar 2012[100]	Retrospective study	European	Hospital Universitari Germans Trias i Pujol, Barcelona/Spain	Jan 2008-Dec 2009	8	≥18yrs (57±19·1)	55·6 [323]	NR	~3,889,692 (2001; ≥19yrs)	581	7·5	2·2 [13] Lp	0·2	NDR
100	Socan 1999[101]	Prospective hospital study	European	Ljubljana/Slovenia	Apr 1996- Mar 1997	5	≥14 yrs (mean 57·2)	50·2 [106]	5 serology; 5 UAT	Total = 276,133 (NB data for 0-14yrs in 1991 was not collected)	211	76·4	2·8 [6] Lp	2·2	NDR

[2] http://wilcoproject.eu/wp-content/uploads/2013/04/WILCO_WP3_Milan_23.pdf

No.	Study Reference	Study Characteristics								CAP Results				*Legionella* Results	
	First Author, Year, Ref	Study Design	WHO Region	City or Region/Country	Study period (for patient recruitment)	Quality Assessment (NOS)	Age group (median or mean (±SD)	Male CAP patients (%) [no.]	*Legionella* Patients Identified by Diagnostic Method No.	Estimated (~) Study Population (Year)	No. of patients with CAP (that meet the criteria)	CAP incidence (annual)	Aetiology of CAP, (%) [no.] of patients *Legionella* spp. Lp = *L. pneumophila*	*Legionella* incidence (annual) of CAP	Case fatality risk *Legionella* spp. No. of patients who died/total no. of patients (%)
101	Sopena 1998[102]	Prospective hospital study	European	Multicentre/Spain	May 1994-Feb 1996	7	≥14yrs - ≤30yrs = 2 (4·1)	74·0 [128]	25 serology; 23 UAT	0-14yrs = 7,532,668 (1991)[3]	173	2·3	27·7 [48] Lp	0·4	NDR
102	Sousa 2013[103]	Prospective hospital study	European	Aragon, Catalonia, Galicia/Spain	May 2005-Jan 2007	5	≥65yrs (75·2 ± 6·5 (immunocompromised; 77·5 ± 7·8 non-immunocompromised	60·3 [193]	15 UAT	≥65yrs (2000) Aragon (255,678), Catalonia (1,020,047), Galicia (522,814)	320	11·9	4·7 [15] Lp	0·5	NDR
103	Spoorenberg 2014[104]	Retrospective (cohort) hospital study	European	Nieuwegein and Ede/The Netherlands	Oct 2004-Sep 2010	9	All ages (63·4±18·0)	58·4 [295]	1 culture; 3 PCR; 15 UAT; 1 serology	~62,600 (Nieuwegein;105,495 (Ede) (2004)	505	50·1	4·0 [20] Lp	2·0	2/20 (10·0)
104	Steinhoff 1996[105]	Prospective hospital study	European	Berlin/Germany	Oct 1991-Oct 1992	5	All ages (range 17-94yrs; mean age, 57yrs)	62·3 [147]	NR* culture, serology	3,260,000 (1987)	236	7·2	1·7 [4] NB specific *Legionella* spp. not recorded	0·1	NDR
105	Stralin 2010[107]	Prospective hospital study	European	Örebro/Sweden	Nov 1999-Apr 2002	6	NAR¤ median 71 (18–96)	52·3 [123]	2 UAT; 1 culture	124,207 (2000)	235	126·1	1·3 [3] Lp	1·6	NDR
106	Tazón-Varela 2016[107]	Prospective hospital study	European	Laredo/Spain	Feb 2012-Feb 2013**	5	≥14yrs (66±22)	57·8 [166]	NR* culture; UAT	100,000 (published)	287	287	0·7 [2] Lp	2	
107	Templeton 2005[108]	Prospective hospital study	European	Leiden/The Netherlands	Sep 2000-Jun 2001	6	≥ 18yrs	71·4 [75]	6 PCR; 2 culture; serology, UAT	117,010 (2001 census)	105	89·7	7·6 [8] Lp (1) NB specific *Legionella* spp. not recorded	6·8	1/8 (12·5)
108	Tilley 2009[109]	Retrospective study	European	Dundee/Scotland	Mar 2001-Feb 2007	6	All ages (mean 51·4)	48·1 [26]	35 UAT; NR* culture; serology	154,674 (2001 census)	54	5·0	64·8 [35] Lp	3·2	NDR
109	Torres 1996[110]	Prospective hospital study	European	Multicentre/Spain	Oct 1992-Dec 1994	6	All ages (67±11·0)	92·7 [115]	NR for 2 cases but 5 serology	38,872,268 (1991)	124	0·1	5·6 [7] Lp	0·001	NDR
110	van de Garde 2008[111]	Prospective hospital study	European	Nieuwegein/The Netherlands	Oct 2004-Aug 2006	7	NAR¤ mean 63±17yrs; CAP ≤60yrs (74) 60–69yrs (39) 70–79yrs (50) >80yrs (38)	61·7 [124]	1 culture; 5 PCR; 6 UAT; 7 serology	62,600 (2004)	201	178·4	9·5 [19] NB specific *Legionella* spp. not recorded	16·9	NDR
111	van der Eerden 2005[112]	Prospective hospital study	European	Alkmaar/The Netherlands	Dec 1998-Nov 2000	7	≥18yrs (mean 64)	53·8 [141]	NR* for culture, serology; 5 UAT	3,658,376 (total) (2000)	262	3·6	5·3 [14] Lp	0·2	NDR
112	van Gageldonk-Lafeber 2013[113]	Prospective (observational) study	European	Hertogenbosch/The Netherlands	Nov 2007-Jan 2010	8	All ages (median 66; range 18-96)	62·5 [212]	NR* culture; serology; PCR; UAT	~134,717 (2006)	339	104·8	2·1 [7] Lp	2·2	NDR
113	Vila-Corcoles 2009[114]	Prospective (population-based cohort) study	European	Tarragona/Spain	Jan 2002-Apr 2005	9	≥65yrs	59·2 [280]	1 serology; 3 UAT	134,232 (19 833 ≥65 yrs) (2003)	473	1703·5	0·8 [4] Lp	14·4	NDR

[3] http://www.ine.es/jaxi/tabla.do

No.	Study Reference	Study Characteristics						CAP Results					*Legionella* Results		
	First Author, Year, Ref	Study Design	WHO Region	City or Region/Country	Study period (for patient recruitment)	Quality Assessment (NOS)	Age group (median or mean (±SD)	Male CAP patients (%) [no.]	*Legionella* Patients Identified by Diagnostic Method No.	Estimated (~) Study Population (Year)	No. of patients with CAP (that meet the criteria)	CAP incidence (annual)	Aetiology of CAP, (%) [no.] of patients<br>*Legionella* spp.<br>Lp = *L. pneumophila*	*Legionella* incidence (annual) of CAP	Case fatality risk *Legionella* spp. No. of patients who died/total no. of patients (%)
114	von Baum 2008[115]	Prospective hospital study	European	Multicentre/Germany	Jun 2002-Apr 2005	7·5	≥18yrs	56·4 [1413]	3 culture; 52 PCR; 48 UAT	~82,689,000 (2005)	2503	1·1	3·8 [94] (88 Lp; 2 *L. anisa*; 2 *L. bozemanii*; 1 *L. longbeachae*; 1 *L. erythra*)	0·04	12/94 (12·8)
115	Walden 2014[116]	Prospective (cohort) study	European	Multinational/17 countries	Sep 2005-Oct 2009	9	≥18yrs (median 64; range 18-101)	62·0 [723]	NR	~8,650,994 Belgium; ~8,693,631 Czech Republic; ~70,576,191 Germany; ~1,146,050 Estonia ~36,776,332 Spain ~51,278,765 France; ~9,484,543 Greece; ~3,775,700 Croatia ~3,258,111 Ireland; ~4,989,600 Israel; ~50,283,255 Italy; ~13,296,952 Netherlands; ~31,796,598 Poland; ~5,709,115 Serbia; ~49,137,395 United Kingdom (2005; ≥15yrs)	1166	0·1	5·6 [65] Lp	0·004	NDR
116	Zalacain 2003[117]	Prospective hospital study	European	Multicentre/Spain	Jan-Dec 1997	7	≥65yrs (76·3±7·3)	63·4 [319]	8 serology; 1 culture; 1 UAT; 9 serology/UAT	≥65 yrs = 6,064,000 (1995)	503	8·3	3·8 [19] Lp	0·3	NDR
117	Zer 2010[118]	Prospective hospital study	European	Gaziantep/Turkey	Nov 2007-Mar 2008	3	NAR¤ (54·67±16·9; range 20-83)	70·1 [54]	5 UAT; 0 culture; 0 serology	1,004,000 (2005)	77	7·7	6·5 [5] Lp	0·5	NDR
118	Al-Ali 2006[119]	Prospective hospital study	Eastern Mediterranean	Irbid/Jordan	Apr-Oct 2002	3	All ages (children mean 3yrs (range 1 mon-13yrs); adult mean 47yrs (range 15-75yrs)	46·0 [47]	NR* serology	751,634 (1994)	101	13·4	2 [2] Lp	0·3	NDR

No.	Study Reference	Study Characteristics								CAP Results				*Legionella* Results	
	First Author, Year, Ref	Study Design	WHO Region	City or Region/Country	Study period (for patient recruitment)	Quality Assessment (NOS)	Age group (median or mean (±SD)	Male CAP patients (%) [no.]	*Legionella* Patients Identified by Diagnostic Method No.	Estimated (~) Study Population (Year)	No. of patients with CAP (that meet the criteria)	CAP incidence (annual)	Aetiology of CAP, (%) [no.] of patients<br>*Legionella* spp.<br>Lp = *L. pneumophila*	*Legionella* incidence (annual) of CAP	Case fatality risk *Legionella* spp. No. of patients who died/total no. of patients (%)
119	Alzeer 1998[120]	Cross-sectional hospital study	Eastern Mediterranean	Makkah/Saudi Arabia	1994 (3-28 May during Hajj)	4	NAR¤ (63±11·9; range 21-91)	73·4 [47]	NR¤	2,000,000 (published)	64	3·2	6·3 [4] Lp	0·2	NDR
120	Asghar 2011[121]	Cross-sectional hospital study	Eastern Mediterranean	Makkah/Saudi Arabia	Jan 2005	4	NAR¤ (range 11->60yrs)	56·6 [40]	NR* serology, culture	1,400,000 (2005)	141	10·1	9·2 [13] Lp	0·2	NDR
121	Behbehani 2005[122]	Prospective hospital study	Eastern Mediterranean	Kuwait	Sep 2000-Sep 2001	5	≥15yrs (4 Lp ≤45yrs; 6 Lp ≥ 46yrs)	49·2 [61]	NR* culture, serology, PCR	1,941,000 (2000)	124	6·4	8·1 [10] Lp	0·5	NDR
122	Ben-Dror 2002[123] (Hebrew)	Prospective hospital study	Eastern Mediterranean	Northern District/Israel	Jun 1999-May 2000	4	≥60yrs	¤NR	8 UAT	5,428 (2000)	202	3721·4	4·0 [8] Lp	147·4	NDR
123	Dahmash 1994[124]	Prospective study	Eastern Mediterranean	Riyadh/Saudi Arabia	Sep 1991-Dec 1992	4	All ages	¤NR	NR* culture; serology	~1,417,000 (1987)	63	3·4	4·8 [3] Lp	0·2	1/3 (33·3)
124	Doaa 2016[125]	Cross-sectional study	Eastern Mediterranean	Zagazig, Sharkya/Egypt	Jan 2013-Jan 2015	5	≥ 5yrs (49·7±10·3)	40·0 [20]	8 PCR	7,000,000 (published)	50	0·4	16 [8] Lp	0·1	NDR
125	El Sayed Zaki 2009[126]	Prospective hospital study	Eastern Mediterranean	Mansoura/Egypt	Oct 2005-Oct 2006	3	≥15yrs (range 18-70)	65·0 [65]	5 culture; 6 serology	439,348 (2006)	100	22·8	6·0 [6] Lp	1·4	NDR
126	Khedri 2015[127]	Prospective hospital study	Eastern Mediterranean	Tehran, Shahrekord/Iran	Nov 2012-Mar 2013	4	≥ 15yrs	60·0 [90]	NR* culture, PCR	7,451,000 (≥ 15yrs) (2011 census)	150	2·0	12 [18] Lp	0·2	NDR
127	Lieberman 2002[128]	Prospective hospital study	Eastern Mediterranean	Beer-Sheva/Israel	Jan-Mar 1999	5	NAR¤ (Age (years; mean _ SD) URTI 35·0 (13·3) LRTI 41·5 (15·4)	46·8 [117]	28 serology	753,500 (1995)	250 [100] inpatients; [150] outpatients	33·2	11·2 [28] NB specific *Legionella* spp. not recorded	3·7	NDR
128	Lieberman 1998[129]	Prospective hospital study	Eastern Mediterranean	Beer-Sheva/Israel	Jan- Mar 1997	3	NAR¤ (44·8 ±14·2; range 21–78yrs)	61·5 [75]	15 serology	753,500 (1995)	122 (NB outpatients only)	16·2	12·3 [15] NB specific *Legionella* spp. not recorded	2·0	NDR
129	Lieberman 1996[130]	Prospective hospital study	Eastern Mediterranean	Negev/Israel	Nov 1991-Oct 1992	3	NAR¤ mean (SD) age = 49·3 (19·5) yrs (range 17-94)<br>CAP 17-44yrs (29); 45-54yrs (8); 55-64yrs (8); 65-74yrs (7); 75+ yrs (4)	54·0 [187]	NR* serology, culture	300,000 (published)	346	115·3	16·2 [56] NB specific *Legionella* spp. not recorded	18·7	NDR
130	Porath 1997[131]	Prospective study	Eastern Mediterranean	Beer-Sheva/Israel	Nov 1991-Oct 1992	4	All ages (mean 49; range 17-94)	52·8 [183]	NR* culture; serology	~573,700 (1990)	346	60·3	16·2 [56] NB specific *Legionella* spp. not recorded	9·8	NDR
131	Shibli 2010[132]	Prospective study	Eastern Mediterranean	Afula/Israel	Nov 2006-Aug 2007	6	≥18yrs (58·3±20·3)	57·9 [73]	9 PCR; 0 serology; 0 culture	~39,686 (2005)	126	317·5	7·1 [9] Lp	22·7	NDR
132	Tremolieres 2005[133] (French)	Prospective hospital study	Eastern Mediterranean	Tunisia	Nov 1999-Feb 2003	6	18-80yrs (47·8±18·3)	67·2 [127]<br>74·4 [136]	NR* serology, PCR	Total = 9,563,500; 0-14yrs = ~1,912,700 (1990)	189 (Group PRI)<br>182 Group AMX)	0·7<br>0·7	11·3 [7] Lp<br>7·5 [5] Lp	0·03<br>0·03	NDR

Supplementary Materials for Graham et al. Global perspective of *Legionella* infection in Community Acquired Pneumonia: A Systematic Review and Meta-analysis of Observational Studies

No.	Study Reference	Study Characteristics								CAP Results			*Legionella* Results		
	First Author, Year, Ref	Study Design	WHO Region	City or Region/Country	Study period (for patient recruitment)	Quality Assessment (NOS)	Age group (median or mean (±SD)	Male CAP patients (%) [no.]	*Legionella* Patients Identified by Diagnostic Method No.	Estimated (~) Study Population (Year)	No. of patients with CAP (that meet the criteria)	CAP incidence (annual)	Aetiology of CAP, (%) [no.] of patients<br>*Legionella* spp.<br>Lp = *L. pneumophila*	*Legionella* incidence (annual) of CAP	Case fatality risk *Legionella* spp. No. of patients who died/total no. of patients (%)
133	Alvarez 2007[134]	Prospective hospital study	Africa	(Tenerife Island) Canary Islands/Africa	Jun 1995-Jun 2006	6	NAR¤ (56±20)	67·5 [510]	NR* serology, culture, UAT	350 000 (published)	755	19·6	2·9 [22] Lp	0·6	NDR
134	Kim 2012[135]	Prospective study	Africa	Dadaab area refugee camp	Oct 2006-Apr 2008	5	NAR¤ (median 1·7yrs (range: 2 mths - 60yrs)	58·4 [66]	PCR	Dadaab (106,000) (United Nations High Commission for Refugees census 1998)	118	31·8	0·8 [1] NB specific *Legionella* spp. not recorded	0·3	NDR
135	Nyamande 2007[136]	Prospective hospital study	Africa	Durban/South Africa	Jun 2000-Oct 2001	3	≥ 60yrs	¤NR	NR*	1,656,120 (65yrs +) (2000)	182	11·0	1·1 [2] Lp	0·1	NDR
136	Paganin 2004[137]	Prospective hospital study	Africa	Réunion Island/Africa	Sep 1995-Dec 2000	7	All ages (54·7±15·1)	83·9 [94]	2 serology; 0 culture	750 000 (published)	112	2·8	1·8 [2] Lp	0·1	0
137	Wolter 2016[138]	Prospective hospital study	Africa	Klerksdorp, North West Province; Pietermaritzburg, KwaZulu-Natal Province/South Africa	Jun 2012-Sep 2014	3	All ages	49·1 [885]	21 PCR	Klerksdorp (398,676) Pietermaritzburg (475,238) (2011 census)	1803	93·9	1·2 [21] Lp	1·1	NDR
138	Bao 2012[139]	Prospective hospital study	Western Pacific	Beijing/China	Jan 2007-Jan 2008	6	≥ 10yrs (1 20+ yrs; 1 30+ yrs; 2 70+yrs)	63·9 [257]	4 serology	15,729,600 ≥ 15yrs (2007)	402 (NB outpatients only)	1·2	1·0 [4] Lp (NB outpatients only)	0·03	NDR
139	Charles 2008[140]	Prospective hospital study	Western Pacific	Brisbane, Melbourne, Perth/Australia	Jun 2004-Sep 2006	7	≥18yrs (65·1±19·9; range 18–100)	60·7 [537]	19 UAT; 11 serology	973,931 (Brisbane, 2005); 1,477,815 (Perth, 2005); 3,634,233 (Melbourne, 2005)	885	6·9	3·4 [30] 25 *Lp*; 3 *L. longbeachae;* 1 *L. micdadei;* 1 undifferentiated	0·3	NDR
140	Chen 2018[141]	Retrospective study	Western Pacific	Multicentre/China	Jan 2014-Dec 2014	7	≥14yrs	53·5 [3117]	227 (IgG) serology; 270 PCR; 47 UAT	≤15yrs 934,935,000 (2013)	5828	0·6	9·3 [544] Lp	0·1	NDR
141	Chen 2015[142]	Prospective hospital study	Western Pacific	Nanjing/China	Aug 2011-Aug 2013	4	≤14yrs	59·4 [715]	11 serology	≤14yrs ~1,240,775 (2010 census)	1204	48·5	0·9 [11] Lp	0·5	NDR
142	Horie 2012[143]	Prospective hospital study	Western Pacific	Tokyo/Japan	Oct 2007-Nov 2008	7	All ages (mean 78 (66·5–82·5)	54·9 [56]	1 UAT	12,369,000 (2003)	102	8·2	1·0 [1] Lp	0·01	NDR
143	How 2011[144]	Prospective study	Western Pacific	Taipei/Taiwan	Sep 2007-Mar 2008	6·5	≥18yrs (50·8±21·3)	68·6 [24]	NR* culture; serology; UAT	~2,615,742 (2006; ≥15yrs)	35	1·3	14·3 [5] NB specific *Legionella* spp. not recorded	0·2	NDR
144	Huang 2006[145]	Prospective hospital study	Western Pacific	Shanghai/China	Oct 2001-Sep 2003	5	≥2yrs (1 Lp 45-49yrs; 1 Lp ≥ 60yrs)	56·0 [218]	NR*serology; UAT	16,407,734 (2000)	389 ([141] outpatients; [248] inpatients)	1·2	0·5 [2] Lp	0·005	NDR
145	Huong 2014[146]	Prospective study	Western Pacific	Ha Noi/Vietnam	Jul 2010-Mar 2012	8	≤15yrs	¤NR	NR* PCR; serology	~1,445,761 (≤15yrs) (2009 census)	722	17·8	1·7 [12] Lp	0·3	NDR
146	Ishida 2014[147]	Prospective study	Western Pacific	Kurashiki/Japan	Jul 1994-Jul 2012	8	≥15yrs	78·3 [361]	NR* culture, UAT	~393,155 (1995 census; ≥15yrs)	461	6·5	4·1 [19] NB specific *Legionella* spp. not recorded	0·3	NDR
147	Ishiguro 2013[148]	Retrospective study	Western Pacific	Saitama/Japan	Jan 2002-Nov 2011	7	All ages (63·9 ± 18·3)	66·2 [683]	16 UAT; NR* serology; culture	1,133,300 (2000 census)	1032	10·1	5·1 [53] NB specific *Legionella* spp. not recorded	0·5	NDR

No.	Study Reference	Study Characteristics								CAP Results			*Legionella* Results		
	First Author, Year, Ref	Study Design	WHO Region	City or Region/Country	Study period (for patient recruitment)	Quality Assessment (NOS)	Age group (median or mean (±SD)	Male CAP patients (%) [no.]	*Legionella* Patients Identified by Diagnostic Method No.	Estimated (~) Study Population (Year)	No. of patients with CAP (that meet the criteria)	CAP incidence (annual)	Aetiology of CAP, (%) [no.] of patients<br>*Legionella* spp.<br>Lp = *L. pneumophila*	*Legionella* incidence (annual) of CAP	Case fatality risk *Legionella* spp. No. of patients who died/total no. of patients (%)
148	Jennings 2008[149]	Prospective study	Western Pacific	Christchurch/New Zealand	Jul 1999-Jul 2000	6	≥18yrs (median 70; range 18-99)	52·0 [158]	NR* serology; culture; PCR	309,027 (1996 census)	304	98·4	3·6 [11] Lp	3·6	NDR
149	Kamata 2015[150]	Prospective hospital study	Western Pacific	Tsukuba/Japan	Jul 2010-Jun 2013	8	≥ 18yrs	67·0 [148]	NR* culture, UAT	(≥ 18yrs) ~183,142 (2010 census)	221	40·2	1·4 [3] Lp	0·5	NDR
150	Kobashi 2007[151]	Prospective hospital study	Western Pacific	Kurashiki/Japan	Oct 2001-Sep 2003	5	≥15yrs (62·1 ± 11·4; range 15-97)	70·5 [110]	2 UAT	500,000 (published)	156	20·8	1·3 [2] Lp	0·3	NDR
151	Laing 2001[152]	Prospective hospital study	Western Pacific	Christchurch and Hamilton/New Zealand	Jul 1999-Jul 2000	6	≥18yrs (median 70; range 18–99)	51·9 [246]	NR* culture, serology, UAT	ChCh 218,000; Hamilton 421,000 (published)	474	74·2	4 [19]<br>NB specific *Legionella* spp. not recorded	3·0	NDR
152	Lauderdale 2005[153]	Prospective hospital study	Western Pacific	Multicentre/Taiwan	Dec 2001-Apr 2002	6	≥16yrs<br>1 Lp (3·3%) 17-44yrs<br>1 Lp (4·5%) 45-59yrs	63·7 [107]	2 UAT	0-15yrs = 4,501,233; total = 21,999,851 (2000)	168	1·0	1·2 [2] Lp	0·01	0
153	Lee 1996[154]	Retrospective study	Western Pacific	Singapore	Jun 1991-Feb 1993	5	All ages (61±17; range 15-86)	54·2 [32]	2 serology	~3,047,000 (1990)	59	1·3	3·4 [2] Lp	0·04	NDR
154	Liu 2006[155] (Chinese)	Prospective hospital study	Western Pacific	Multicentre/China (Beijing, Shanghai, Shenyang, Xi'an, Chengdu, Guangzhou, and Hangzhou)	Dec 2003-Nov 2004	3	≥18yrs (52·4±20·0)	63·0 [419]	NR* culture; serology; UAT	1,280,429,000 (2000); NB 0-14yrs = 327,734,000 (2000)	665	0·1	2·6 [17] Lp	0·002	NDR
155	Liu 2004[156]	Prospective hospital study	Western Pacific	Beijing/China	Nov 2001-Jun 2002	3	≥15yrs	¤NR	3 UAT	11,940,000 (2000)	103	0·9	1·9 [2] Lp	0·03	NDR
156	Lui 2009[157]	Prospective hospital study	Western Pacific	Hong Kong/China	Jan 2004-Jun 2005	7	≥18yrs (70·8±18·0)	59·3 [707]	NR* culture; serology; UAT	6,897,000 (2004)	1193	11·5	0·1 [1] Lp	0·007	NDR
157	Miyashita 2005[158]	Prospective ambulatory and hospitalized patient study	Western Pacific	Kawasaki and Kurashiki/Japan	Apr 1998- Dec 2003	6	≥16yrs (mean 58·3; range 16-97)	61·9 [313]	NR* culture; serology; UAT	1,249,851 (2005 – Kawasaki); 430,291 (2005 –Kurashiki)	506	5·7	1·2 [6] Lp (NB in-patients only; [0] out-patients)	0·1	NDR
158	Neill 1996[159]	Prospective hospital study	Western Pacific	Christchurch/New Zealand	Jul 1992-Jul 1993	7	NAR¤ (mean 58; range 18-97)	55·0 [140]	NR*	350,000 (published)	255	72·9	10·6 [27]<br>13 *Lp*; 6 *L. micdadei*; 2 *L longbeachae*; 2 *L dumoffii*;1 *L jordanis*; 3 dual identifications	7·7	2/27 (7·4)
159	Richards 2005[160]	Prospective hospital (randomised control) study	Western Pacific	Christchurch/New Zealand	Jul 2002-Oct 2003	4	NAR¤ (mean 50·1yrs (home), 49·8 (hospital))	53·1 [26]	2 serology	316,227 (2001)	49 [25] inpatients; [24] outpatients	12·9	4·1 [2] Lp	2·3	0
160	Saito 2006[161]	Prospective hospital study	Western Pacific	Multicentre/Japan	Dec 1999-Mar 2000	6	NAR¤ (mean 60·2; range 17-99)	57·8 [134]	NR* culture; PCR;	126,650,000 (1999)	232 [200] inpatients;	0·2	3·9 [9]	0·01	NDR

Supplementary Materials for Graham et al. Global perspective of *Legionella* infection in Community Acquired Pneumonia: A Systematic Review and Meta-analysis of Observational Studies

No.	Study Reference	Study Characteristics								CAP Results			*Legionella* Results		
	**First Author, Year, Ref**	**Study Design**	**WHO Region**	**City or Region/Country**	**Study period (for patient recruitment)**	**Quality Assessment (NOS)**	**Age group (median or mean (±SD)**	**Male CAP patients (%) [no.]**	***Legionella* Patients Identified by Diagnostic Method No.**	**Estimated (~) Study Population (Year)**	**No. of patients with CAP (that meet the criteria)**	**CAP incidence (annual)**	**Aetiology of CAP, (%) [no.] of patients *Legionella* spp. Lp = *L. pneumophila***	***Legionella* incidence (annual) of CAP**	**Case fatality risk *Legionella* spp. No. of patients who died/total no. of patients (%)**
									serology; UAT		[32] outpatients		NB specific *Legionella* spp. not recorded		
161	Shindo 2013[162]	Prospective study	Western Pacific	(Central Japan Lung Study Group), Nagoya/Japan	Mar 2010-Dec 2010	8	≥20yrs (median 75)	65·4 [580]	NR	~1,992,227 (2010 census; ≥15yrs)	887	44·5	0·8 [7] Lp	0·4	NDR
162	Sohn 2006[163]	Prospective hospital study	Western Pacific	Multicentre (Seoul and Kyungi provinces)/South Korea	Oct 2001- Dec 2002	5	≥16yrs (54·6±17·8)	56·3 [71]	3 serology	10,280,523 (Seoul 2002); 10,000,047 (Kyungi, 2002)	126	0·6	2·4 [3] Lp	0·01	NDR
163	Song 2008[164]	Prospective hospital study	Western Pacific	Multinational/8 countries South Korea, China, Taiwan, Hong Kong, India, Vietnam, The Philippines, Singapore	Jan 2002- Dec 2004	8	≥15yrs (57·3±13·7; range 16–94)	63·2 [604]	7 UAT	≥15yrs (2000) Hong Kong (5,536,900); Singapore (2,562,411); Manila (1,104,009); Taipei City, Taiwan (2,126,707); Beijing, China (11,940,000); Shanghai, China (14,700,000); Vellore, India (2001) (2,576,336); Ho Chi Minh City Vietnam (1999) (3,976,906); South Korea (2000) Seoul (8,054,707), Daegu (1,967,099), Daejeon (1,058,207), Gwangju (1,041,000); Total (56,644,282)	955	0·6	0·7 [7] Lp	0·003	NDR
164	Su 2005[165]	Prospective hospital study	Western Pacific	Taiwan	Jan 2001-Dec 2003	5	All ages	70·9 [168]	13 culture; 211 serology; 41 UAT	22,370,460 (2001)	5097	7·6	4·6 [237] Lp and *L. dumoffii*	0·4	11/237 (0·05)
165	Takaki 2014[166]	Prospective hospital study	Western Pacific	Kochi/Japan	May 2008-Apr 2010	5	≥15yrs (median 77; IQR 63-84)	67·9 [89]	2 PCR; 2 UAT	340,208 (published)	131	19·3	3·1 [4] Lp	0·6	NDR
166	Takayanagi 2006[167] (Japanese)	Retrospective hospital study	Western Pacific	Kumagaya (Saitama) /Japan	Jan 2002-Dec 2005	4	≥15yrs; *Legionella* cases 15-44yrs =2; 45-64yrs = 11; 65-74yrs = 4; 75-93yrs = 9	72·5 [473]	15 UAT; 11 either culture or serology	5,900,445 (≥15yrs) (2000)	652	2·8	4·0 [26] NB specific *Legionella* spp. not recorded	0·1	4/26 (15·4)
167	Tao 2012[168]	Prospective (multi-centred) hospital study	Western Pacific	Multicentre (36 centers in 22 cities of 16 provinces: Beijing, Shanghai, Tianjin, Guangdong (Guangzhou, Shenzhen), Liaoning (Shenyang), Shandong (Jinan, Qingdao), Jiangsu (Nanjing, Suzhou, Wuxi),	Jun 2004-Aug 2005	5	≥ 18yrs (51·9±19·2; range 18–97)	57·2 [339]	13 serology	~679,349,589 (2000) ≥15yrs	593	0·1	2·2 [13] Lp	0·002	NDR

No.	Study Reference			Study Characteristics						CAP Results			*Legionella* Results		
	First Author, Year, Ref	Study Design	WHO Region	City or Region/Country	Study period (for patient recruitment)	Quality Assessment (NOS)	Age group (median or mean (±SD)	Male CAP patients (%) [no.]	*Legionella* Patients Identified by Diagnostic Method No.	Estimated (~) Study Population (Year)	No. of patients with CAP (that meet the criteria)	CAP incidence (annual)	Aetiology of CAP, (%) [no.] of patients *Legionella* spp. Lp = *L. pneumophila*	*Legionella* incidence (annual) of CAP	Case fatality risk *Legionella* spp. No. of patients who died/total no. of patients (%)
				Zhejiang (Hangzhou, Ningbo, Jiaxing), Anhui (Hefei), Jiangxi (Nanchang), Shanxi (Xi'an), Henan (Zhengzhou), Hubei (Wuhan), Hunan (Changsha), Sichuan (Chengdu), and Yunnan (Kunming)/ China											
168	Ugajin 2014[169]	Prospective (observational) study	Western Pacific	Ichinomiya City/Japan	Aug 2010-Oct 2012	4	≥18yrs (mean 82; range 74–88)	59·6 [127]	1 UAT	~305,746 (2010 census; ≥15yrs)	213	34·8	0·5 [1] Lp	0·2	NDR
169	Watari 1999[170]	Prospective study	Western Pacific	Chigasaki/Japan	May 1996-Oct 1997	5	All ages	59.5 [44]	NR*serology; UAT; PCR	~212,874 (1995 census)	74	34.8	6.8 [5] NB specific *Legionella* spp. not recorded	2.3	NDR
170	Wilson 2005[171]	Retrospective study	Western Pacific	Newcastle/Australia	Jan 2001-Jul 2003	6	≥18yrs (59·5±16·6; range 21-88)	56·3 [54]	NR* culture; serology; UAT DFA; PCR	112,038 (2001 census; ≥15yrs)	96	57·1	1·0 [1] *L. longbeachae*	0·6	0
171	Xiong 2010[172] (Chinese)	Prospective study	Western Pacific	Beijing/China	May 2005-May 2008	3	All ages (65·4 ±15·2; range 19-93)	69·3 [106]	5 serology	~13,569,194 (2000)	153	0·4	3·3 [5] Lp	0·1	NDR
172	Yeh 2007[173]	Prospective hospital study	Western Pacific	(northern) Taiwan	Jul 1997-Oct 1998	3	NAR¤ (mean 55·7; range 19-95)	¤NR	4 serology; 10 UAT	21,743,000 (1997)	323	1·5	4·0 [13] Lp NB possible cases (16) excluded	0·1	7/13 (53·8)
173	Yen 2005[174]	Prospective hospital study	Western Pacific	Taiwan	Feb 2001-Jan 2002	5	≥18yrs	57·0 [57]	2 serology; 1 UAT	3,000,000 (published)	100	3·3	3·0 [3] Lp	0·1	NDR
174	Yoshimoto 2005[175]	Case-control study	Western Pacific	Toyama city/Japan	Apr1995-Mar 2002	6·5	≥18yrs (mean 72·9)	75·0 [54]	NR* culture; serology	~364,969 (1995 census; ≥15yrs)	72	3·3	2·8 [2] NB specific *Legionella* spp. not recorded	0·1	2/2 (100)
175	Zhang 2017[176] (Chinese)	Prospective hospital study	Western Pacific	Beijing/China	Jan 2000-Dec 2015	4	¤NAR	68·8 [128]	16 culture; 0 serology	~21,520,000 (2015)	186	0·1	8·6 [16] Lp	0·006	NDR
176	Anbumani 2010[177]	Prospective study	South-East Asian	Tirupati/India	Jul 2007-Dec 2008	3	All ages	¤NR	12 culture	~26,000	470	1205·1	2·6 [12] Lp	30·8	NDR
177	Angrup 2016[178]	Retrospective observational study	South-East Asian	New Delhi/India	May 2005-Aug 2008	4	All ages	68·7 [92]	14 PCR; 32 serology; 0 culture	13,451,000 (2005)	134	0·4	23·9 [32] Lp	0·1	NDR
178	Chaudhry 2000[179]	Prospective hospital study	South-East Asian	New Delhi/India	Apr 1997- Dec 1998	3	NAR¤ (37·96+24·0; range 8 mths - 78)	75·0 [45]	9 serology; 0 culture	9,420,644 (1991)	60	0·3	15 [9] Lp	0·1	NDR
179	Javed 2010[180]	Case-control study	South-East Asian	New Delhi/India	May 2005-Jan 2008	3·5	NAR¤ (37·96+24·8)	66·4 [75]	2 serology; 2 UAT	13,451,000 (2005)	113	0·3	1·8 [2] Lp	0·004	NDR
180	Liam 2006[181]	Prospective hospital study	South-East Asian	Kuala Lumpur/Malaysia	NR* (2·3 yr study)	6	≥12yrs	56·1 [194]	NR* culture; serology; UAT	1,039,447 (≥15yrs) (2000)	346	33·3	4·0 [14] Lp NB presumptive cases excluded	1·3	NDR
181	Mustafa 2011[182]	Prospective hospital study	South-East Asian	Pahang/Malaysia	Oct 2009-Mar 2010	4	≥15yrs	¤NR	1 PCR	738,000 (≥18yrs) (2002)	46	6·2	2·2 [1] Lp	0·1	NDR

No.	Study Reference				Study Characteristics					CAP Results				*Legionella* Results	
	First Author, Year, Ref	Study Design	WHO Region	City or Region/Country	Study period (for patient recruitment)	Quality Assessment (NOS)	Age group (median or mean (±SD)	Male CAP patients (%) [no.]	*Legionella* Patients Identified by Diagnostic Method No.	Estimated (~) Study Population (Year)	No. of patients with CAP (that meet the criteria)	CAP incidence (annual)	Aetiology of CAP, (%) [no.] of patients<br>*Legionella* spp.<br>Lp = *L. pneumophila*	*Legionella* incidence (annual) of CAP	Case fatality risk *Legionella* spp. No. of patients who died/total no. of patients (%)
							(54·7 ±15·3; range 17–80)								
182	Nadar 1997[183]	Retrospective hospital study	South-East Asian	Vellore, India	Jan 1993-Jul 1994	4	NAR¤	66·4 [83]	9 serology	175,061 (1991)	125	47·6	7·2 [9] Lp	3·4	NDR
183	Ngeow 2005[184]	Prospective hospital study	South-East Asian	Multicentre (Beijing, Shanghai, Hong Kong, Seoul, Taipei, Bangkok, Manila, Kuala Lumpur, Petaling Jaya, Singapore, Jakarta, Surabaya)	Oct 2001-Dec 2002	5	≥ 2yrs CAP (327 (2-5yrs); 56 (16-20yrs); 121 (6-15yrs); 330 (21-50yrs); 540 (≥51yrs)	56·9 [782]	57 serology; 61 PCR and serology; 25 UAT	Totals for 2000 Beijing (11,510,000);Shanghai (14,350,000); Hong Kong (6,665,000); Seoul (9,895,000); Taipei (22,000,000); Bangkok (6,355,000); Manila (9,400,000); Kuala Lumpur (1,306,000); Petaling Jaya (438,084); Singapore (4,028,000); Jakarta (8,384,853); Surabaya (2,599,796)	1374 (paediatric outpatients = 58; inpatients = 390; adult outpatients = 105; inpatients = 821)	60·7	6·6 [91] LpSg1 6·6 [6] outpatients; 93·4 [85] inpatients NB paediatric patients were not screened for Legionellosis	0·08	NDR
184	Olsen 2010[185]	Prospective hospital study	South-East Asian	Sa Kaeo and Nakhon Phanom/Thailand	Sep 2003-Dec 2005	3·5	≥18yrs (18-49yrs; 7 cases *L. longbeachae)*; ≥50yrs 13 cases *L. longbeachae*)	58·6 [1013]	NR* serology; UAT; PCR	Sa Kaeo (pop 514,065 in 2003); Nakhon Phanom (pop 730,659 in 2005) (published)	1730	60·4	1·2 [20] 20 *L. longbeachae*; 0 Lp	0·7	NDR
185	Phares 2007[186]	Prospective hospital study	South-East Asian	Sa Kaeo/Thailand	Sep 2003-Aug 2004	6	≥18yrs (median 60yrs)	61·3 [463]	20 serology	438,557 (published)	755	172·2	2·6 [20] 20 *L. longbeachae*; 0 Lp	4·6	NDR
186	Prapphal 2006[187]	Prospective (multi-centred) hospital study	South-East Asian	Bangkok/Thailand	Dec 2001-Nov 2002	5	≥ 16yrs (15 = 16-30yrs, 16 = 31-60yrs, 16 = ≥61yrs) Mean age (yrs) ± SD 36·8 ± 22·3	53·8 [157]	1 (4-fold rising & PCR1 / EIA2+ high antibody), 1 serology	5,293,835 ≥ 15yrs (2000)	292	5·5	0·7 [2] Lp Inpatients 100 [2]; outpatients [0]	0·04	NDR
187	Wattanathum 2003[188]	Prospective hospital study	South-East Asian	Bangkok/Thailand	Sep 1998-Apr 2001	6	≥15yrs (outpatients 35·5 ±16·7 (16–86); inpatients 58·9±20·4 (18–98))	61·6 [151]	NR* culture; serology; UAT	59,450,818 (total) (1997) 0-14yrs: 14,788,766)	292 (paediatric outpatients = 43; inpatients = 202; adult outpatients = 15; inpatients = 32)	0·2	3·3 [8] Lp inpatients; 8·2 [8] Lp outpatients	0·004 0·004	NDR
188	Arancibia 2014[189]	Prospective hospital study	The Americas	Multicentre/Chile	Jan 2005-Jun 2006	6	≥18yrs (mean 58·2; range 19-89)	64·4 [67]	9 UAT	16,295,000 (total) (NB 4,057,455 = 0-14yrs) (2005)	104	0·6	8·7 [9] Lp	0·1	NDR

No.	Study Reference	Study Characteristics								CAP Results			*Legionella* Results		
	First Author, Year, Ref	Study Design	WHO Region	City or Region/Country	Study period (for patient recruitment)	Quality Assessment (NOS)	Age group (median or mean (±SD)	Male CAP patients (%) [no.]	*Legionella* Patients Identified by Diagnostic Method No.	Estimated (~) Study Population (Year)	No. of patients with CAP (that meet the criteria)	CAP incidence (annual)	Aetiology of CAP, (%) [no.] of patients<br>*Legionella* spp.<br>Lp = *L. pneumophila*	*Legionella* incidence (annual) of CAP	Case fatality risk *Legionella* spp. No. of patients who died/total no. of patients (%)
189	Bellew 2018[190]	Cross-sectional hospital study	The Americas	Chicago, Illinois, Nashville, Tennessee/United States	Jan 2010-Jun 2012	8	≥18yrs (median 57yrs (46-71)	49·4 [958]	32 UAT	Total= 2,544,681 (2010 census ≥18yrs); Chicago (2,073,968); Nashville (470,713)	1941	76·3	1·6 [32] Lp	1·3	NDR
190	Caberlotto 2003[191] (Spanish)	Prospective hospital study	The Americas	Buenos Aires/Argentina	Mar 1998-May 2001	4	≥65yrs (range 16-90yrs)	49·3 [37]	9 serology	3,040,000 (total) (1998); 510720 ≥65yrs	75	4·9	12 [9] NB specific *Legionella* spp. not recorded	0·3	0
191	Chedid 2005[192]	Prospective (cohort) hospital study	The Americas	Porto Alegre/Brazil	2000-2001	3	≥18yrs – ≤80yrs	66·1 [39]	NR* serology; UAT	1,004,482 (≥15yrs ≤ 79yrs) (2000)[4]	59	3·0	11·9 [7] Lp	0·4	0
192	Corrêa 2001[193] (Portuguese)	Retrospective hospital study	The Americas	Belo Horizonte/Brazil	May 1998-May 1999	5	All ages (64·7 ± 16·8; (range 20–91)	64·3 [27]	2 serology	2,048,851 (1991)	42	2·0	4·8 [2] Lp	0·1	NDR
193	Diaz 2007[194]	Prospective (cohort) hospital study	The Americas	Multicentre/Chile	Feb 2004-Apr 2005	6	≥16yrs (65·8±18·5)	51·7 [91]	4 UAT	16,295,000 (total) (NB 4,057,455 = 0-14yrs) (2005)	176	0·8	2·3 [4] Lp	0·02	NDR
194	El-Solh 2001[195]	Prospective (cohort) hospital study	The Americas	Buffalo/United States	Jun 1996-Sep 1999	7	≥75yrs (82·3±5·5)	¤NR	5 serology	≥75yrs (21,240) (1990); total 328,123 (1990)	104	188·3	4·8 [5] Lp	9·0	3/5 (60)
195	Gelfer 2015[196]	Prospective hospital study	The Americas	Portland, Oregon/United States	Jan-Mar 2014	6·5	≥ 18yrs	¤NR (only partially)	1 PCR, 1 UAT, 0 culture	(≥ 18yrs) 417,667 (2000 census)	142	34·0	1·4 [2] Lp	0·5	NDR
196	Harris 2017[197]	Prospective hospital study	The Americas	Memphis, Nashville, Salt Lake City, Chicago/United States	Jan 2010-Jun 2012	6	All ages	¤NR	26 UAT, NR* (PCR, culture)	Total (2010) (4,137,341) Memphis (652,597), Nashville (601,222), Salt Lake City (186,522), Chicago (2,697,000)	4676	45·2	0·6 [30] Lp	0·3	NDR
197	Henry 2017[198]	Retrospective multicentre cohort study	The Americas	Central Texas/United States	Jan 2001-Dec 2013	4	All ages (65·3±17·5)	48·8 [2835]	17 UAT	1,759,039 (2010 decennial census in 6 county central Texas region)	5807	25·4	0·3 [17] Lp	0·1	5/17 (29·4)
198	Hollenbeck 2011[199]	Retrospective hospital study	The Americas	Rhode Island/United States	Jan 2005-Dec 2009	4	≥18yrs	¤NR	41 UAT; culture	800,047 (≥18yrs) (2000 census)	3982	124·4	0·9 [41] Lp	1·3	6/37 (16·2)
199	Jain 2015[200]	Prospective hospital study	The Americas	Chicago;Nashville/United States	Jan 2010-Jun 2012	7·5	≥18yrs	¤NR	NR* PCR; UAT	Total= 2,544,681 (2010 census ≥18yrs); Chicago (2,073,968); Nashville (470,713)	2488	97·8	1·5 [37] Lp	1·5	NDR
200	Johnstone 2008[201]	Prospective hospital study	The Americas	Alberta/Canada	Jan 2004-Jan 2006	5	≥18yrs	51·0 [98]	NR* serology; PCR	1,101,225 (≥18yrs) (2001)	193	8·8	0·5 [1] Lp	0·1	NDR

[4] http://www.ibge.gov.br/home/

No.	Study Reference			Study Characteristics						CAP Results			*Legionella* Results		
	**First Author, Year, Ref**	**Study Design**	**WHO Region**	**City or Region/Country**	**Study period (for patient recruitment)**	**Quality Assessment (NOS)**	**Age group (median or mean (±SD)**	**Male CAP patients (%) [no.]**	***Legionella* Patients Identified by Diagnostic Method No.**	**Estimated (~) Study Population (Year)**	**No. of patients with CAP (that meet the criteria)**	**CAP incidence (annual)**	**Aetiology of CAP, (%) [no.] of patients *Legionella* spp. Lp = *L. pneumophila***	***Legionella* incidence (annual) of CAP**	**Case fatality risk *Legionella* spp. No. of patients who died/total no. of patients (%)**
201	Lopardo 2002[202] (Spanish)	Prospective hospital study	The Americas	Buenos Aires/Argentina	Sep 1992-May 1995	3	≥18yrs	55·0 [51]	3 serology	2,508,000 (1998; ≥15yrs)	92	1·4	3·3 [3] Lp	0·04	NDR
202	Luchsinger 2013[203]	Prospective hospital study	The Americas	Santiago/Chile	Feb 2005- Dec 2007	6	≥18yrs	53·7 [191]	3 culture; 10 UAT; all cases confirmed by PCR	4,043,669 (≥15yrs) (2000)[5]	356 [330] inpatients; [26] outpatients	4·4	3·7 [13] Lp	0·2	NDR
203	Luna 2004[204] (Spanish)	Retrospective (cohort) hospital study	The Americas	Buenos Aires/Argentina	1997-2001	3	(66±16 – *Legionella* & CAP); (67±21 CAP only)	¤NR	NR* culture, serology, UAT	13,827,203 (2001)	697	1·0	1·3 [9] Lp	0·02	2/9 (22·2)
204	Marrie 2003[205]	Prospective (observational) study	The Americas	Multicentre/Canada	Jan 1996-Oct 1997	5	≥16yrs	¤NR	NR* UAT, serology, culture	~ 22,350,000 (1991 census; ≥13yrs)	850	2·5	3·3 [28] Lp	0·1	2/28 (7·1)
205	Marrie 1996[206]	Prospective (cohort) hospital study	The Americas	Nova Scotia/Canada	Dec 1991-Mar 1994	4	≥18yrs	36·2 [54]	NR* serology	673,942 (1991 census; 226,000 0-17yrs	149	17·0	0·7 [1] Lp	0·1	NDR
206	Marston 1997[207]	Prospective hospital study	The Americas	Ohio/United States	Jan-Dec 1991	6·5	≥18yrs	51·4 [1427]	NR* culture, serology	Franklin county (~706, 000); Summit county (~380, 000) (published)	2776	255·6	1·7 [47] NB possible cases excluded; specific *Legionella* spp. not recorded	4·4 NB possible cases excluded	NDR
207	McNally 2000[208]	Retrospective serological survey	The Americas	Ohio/United States	Mar 1992-Mar 1993	3	NAR¤ (range 21-80yrs, median 55yrs)	¤NR	14 serology	10,847,115 (1990)	99	0·9	14·1 [14] 8 *L. bozemanae*; 4 *L. anisa*; 2 dual identifications	0·1	NDR
208	Micek 2007[209]	Retrospective (cohort) hospital surveillance	The Americas	St· Louis City/United States	Jan 2003-Dec 2005	7	NAR¤ (58·9±18·1; (range 17-102)	58·2 [121]	8 UAT	348,189 (2000)	208	29·9	3·8 [8] Lp	0·8	NDR
209	Mundy 1995[210]	Cross-sectional and prospective study	The Americas	Maryland/United States	Nov 1990-Nov 1991	6	≥17yrs (mean 45·3; range 17-94)	63·4 [244]	NR* culture; serology; DFA; UAT	3,788,802 (≥15yrs; 1990)	385	10·2	3·4 [13] Lp	0·3	NDR
210	Palombini 1997[211] (Portuguese)	Prospective study	The Americas	Porto Alegre/Brazil	Jan 1993-May 1994	3	≥18yrs	¤NR	2 serology	914,782 (1991 census; ≤15yrs)	15	1·2	13·3 [2] Lp	0·2	NDR
211	Park 2001[212]	Prospective hospital study	The Americas	Seattle/United States	Jun 1994-May 1996	4·5	≥18yrs	77·8 [406]	NR* culture; serology	2,970,300 (1990)	522	8·9	3·4 [18] NB possible cases excluded; specific *Legionella* spp. not recorded	0·3	NDR
212	Peñafiel 2016[213] (Spanish)	Prospective study	The Americas	Santiago/Chile	Jun 2014-Dec 2015	4	≥18yrs (71±18; range 18-102)	50·0 [120]	NR* culture; UAT; serology	4,043,669 (≥15yrs) (2000)[6]	240	5·9	0·4 [1] Lp	0·02	NDR

[5] Economic Commission for Latin America and the Caribbean (ECLAC)
[6] Economic Commission for Latin America and the Caribbean (ECLAC)4

No.	Study Reference	Study Characteristics								CAP Results			*Legionella* Results		
	**First Author, Year, Ref**	**Study Design**	**WHO Region**	**City or Region/Country**	**Study period (for patient recruitment)**	**Quality Assessment (NOS)**	**Age group (median or mean (±SD)**	**Male CAP patients (%) [no.]**	***Legionella* Patients Identified by Diagnostic Method No.**	**Estimated (~) Study Population (Year)**	**No. of patients with CAP (that meet the criteria)**	**CAP incidence (annual)**	**Aetiology of CAP, (%) [no.] of patients<br>*Legionella* spp.<br>Lp = *L. pneumophila***	***Legionella* incidence (annual) of CAP**	**Case fatality risk *Legionella* spp. No. of patients who died/total no. of patients (%)**
213	Plouffe 1996[214]	Randomised hospital study	The Americas	Franklin county, Ohio/United States	Jun 1993-May 1994	6	≥18yrs	50·7 [151]	NR* culture; serology; UAT	~706,000 (refer to Marston 1997)	298	42·2	2·7 [8] Lp	1·1	NDR
214	Rocha 2000[215]	Retrospective hospital study	The Americas	São Paulo/Brazil	Mar 1995-Jan 1997	4	≥ 13yrs (37 ± 17; range 14-85yrs)	66·7 [46]	4 serology	~ 7,274,934 ≥ 13yrs (1996)	69	0·6	5·8 [4] NB specific *Legionella* spp. not recorded	0·1	NDR
215	Tan 2000[216]	Prospective hospital study	The Americas	Summit County, Ohio/ United States	Nov 1990-Nov 1992	3·5	≥ 18yrs	¤NR (only for *Legionella* patients)	18 UAT; 32 serology; 3 culture	≥ 16yrs 402,226 (1990)	2750	341·9	1·6 [43] NB specific *Legionella* spp. not recorded	5·4	2/43 (0·05)
216	Touray 2014[217]	Retrospective hospital study	The Americas	Milford, Massachusetts/Unites States	Jul 2012-Aug 2013	5	All ages	¤NR (only for *Legionella* patients)	6 UAT	All ages 27,999 (2010 census)	266	950·0	2·3 [6] Lp	21·4	NDR
217	Vanina 2009[218] (Spanish)	Prospective study	The Americas	Buenos Aires/ Argentina	Oct 1997-May 2003	3·5	≥17yrs (71·1±16·1)	¤NR	NR* serology; culture; UAT	~2,508,000 (1998; ≥15yrs)	687	6·1	0·4 [3] NB specific *Legionella* spp. not recorded	0·03	NDR
218	Vergis 2000[219]	Prospective randomised, comparative hospital study	The Americas	Little Rock, Arkansas/ Pittsburgh, Pennsylvania/Akron, Ohio/ San Jose, California/United States	1994-1996	7·5	≥18yrs	¤NR	NR* culture; serology; UAT	132,042 (Little Rock, 1990); 296,500 (Pittsburgh, 1990); 168,468 (Akron, 1990); 573,320 (San Jose, 1990)	145	4·1	13·8 [20] Lp	0·6	NDR
219	Carugati 2018[220]	Prospective (cohort)	All	International; multicentre	Mar-Jun 2015	8	≥ 18yrs (median 68yrs)	58·7 [1888]	1113 UAT	~ 5,355,269,019 (54 countries) (2015)	3702	0.1	30·1 [1113] Lp NB secondary analysis 2019 [31 immunocompromi sed]	0.02	NDR
	Total							[54412]			125,764		[5,723]		

** Confirmation sort from the principal author as study period not explicit in the paper
CAPNETZ = the German competence network for community acquired pneumonia
*NR = Number of patients identified by each diagnostic test was not reported
LRTI = Lower respiratory tract infection URTI = Upper respiratory tract infection
¤NR = No data on sex reported
NA = No association
NDR = No data reported on mortality of patients with confirmed *Legionella*
PCR = Polymerase chain reaction
UAT = Urinary antigen test

Table S3. Patient and study characteristics of included studies where *Legionella* was not detected as the cause of CAP

No.	Study Reference	Patient and Study Characteristics								CAP Results	
	First Author, Year, Ref	Study Design	WHO Region	City or Region/Country	Study period (for patient recruitment)	Age group (median or mean (±SD), range)	Male CAP patients (%) [no.]	Diagnostic method used to detect *Legionella*	Estimated (~) Study Population (Year)	No. of patients with CAP (that meet the criteria)	CAP incidence (annual)
1	Holm 2007[221]	Prospective	European	Odense/Denmark	Sep-Nov 2002; Jan-Apr 2003	All ages (median 50yrs)	13·1 [47]	PCR	185,000 (published)	48	25·9
2	Falguera 2009[222]	Prospective	European	Barcelona & Lleida/Spain	Feb 1995-Dec 2005; Jan 1999-Dec 2005	≥ 18yrs (mean 64yrs; range 18–100yrs)	69 [2257]	culture, serology, UAT	Barcelona (≥19yrs 3,889,692 (2001); Lleida 113,040 (2001))	3272	81·7
3	Lagerström 2003[223]	Prospective	European	Örebro/Sweden	Nov 1995-Oct 1998	≥ 11yrs (mean 51yrs; range 10–84yrs)	36·7 [65]	culture, serology	120,000 (published)	180	150
4	Naderi 2015[224]	Prospective (observational)	Eastern Mediterranean	Mashhad/Iran	Feb2013-Jan 2014	≥ 16yrs (mean 50.4 ± 22.6yrs; range 17-94)	63·3 [76]	UAT	2,307,177 (2012)	120	5·2
5	Lee 2002[225]	Prospective	Western Pacific	Chunchon/South Korea	Sep 1999-Aug 2000	66.3 ± 14.5yrs (range: 17-92yrs)	NR	serology	251,991 (2000)	81	32·1
6	Dissanayake 2016[226]	Prospective	South-East Asian	Peradeniya (Kandy)/Sri Lanka	Jul 2014-Jun 2015	NR	NR	serology, UAT	~ 30,000 (2009)	80	266·7
7	Kühne 2014[227]	Prospective	The Americas	Porto Alegre/Brazil	Oct 2010-Jul 2013	≥ 18yrs (mean 61.7 ± 19.8yrs)	56·8 [54]	UAT, PCR	1,145,082 (≥ 16yrs 2010)	95	8·3
8	Musher 2013[228]	Prospective	The Americas	Houston/United States	Jul 2011-Jun 2012	All ages (69.8 ± 11.9)	95·7 [268]	UAT	~ 2,125,000 (2011)	259	12·2

Table S4. Patient and study characteristics of excluded studies from the meta-analysis that reported data on overlapping participant populations

No.	Study Reference			Patient and Study Characteristics					CAP Results			*Legionella* results	
	First Author, Year, Ref	Study Design	WHO Region	City or Region/Country	Study period (for patient recruitment)	Age group (median or mean (±SD), range)	Male CAP patients (%) [no.]	Diagnostic method used to detect *Legionella*	Estimated (~) Study Population (Year)	No. of patients with CAP (that meet the criteria)	CAP incidence (annual)	Aetiology of CAP (%) [no.] of patients	*Legionella* incidence (annual) of CAP
1	Alvarez 2001[229]	Prospective hospital study	European	Seville/Spain	May 1996-May 2000	≥60 yrs	68·0 [51]	6 serology	279, 226 (2000) (≥60 yrs)	75	6·7	2·1 [6] Lp	0·5
2	Arancibia 2002[230]	Prospective hospital study	European	Hospital Clinic of Barcelona/Spain	Jan 1997-Dec 1998	NAR¤ (72 ± 13)	67·8 [379]	29 serology or UAT	1,508,805 (1996)	559	37·0	5·1 [29] NB specific *Legionella* spp. not recorded	1·9
3	Carratala 2007[231]	Prospective observational study	European	Hospital Universitari de Bellvitge, Barcelona/Spain	Jan 2001-Dec 2004	≥18yrs	70·8 [515]	56 UAT	≥19yrs 3,889,692 (2001)	727	18·7	7·7 [56] Lp (HCAP=3; CAP=53)	1·4
4	Cillóniz 2012[232]	Prospective observational study	European	Hospital Clinic of Barcelona/Spain	Jan 2000-Jul 2010	≥16yrs (47·2 ±17·6)	53·0 [301]	13 UAT, 0 serology, 0 culture	540,000 (published)	568	11·1	2·3 [13] Lp	0·3
5	Cillóniz 2011[233]	Prospective hospital study	European	Hospital Clinic of Barcelona/Spain	Nov 1996-Jul 2008	NAR¤ (65·5± 18·1; range 18-102)	63·0 [2218]	NR* culture, serology, UAT	~1,508,805 (1996)	3523	31·1	3·3 [118] Lp	1·0
6	Cillóniz 2011[234]	Prospective observational hospital study	European	Hospital Clinic of Barcelona/Spain	Jan 2003-Dec 2010	NAR¤ (63·4± 16·5)	64·1 [362 ICU CAP]	NR*culture, UAT, PCR	1,503,884 (2001)	2,200	146·3	3·0 [11ICU CAP] Lp	0·04
7	Curran 2008[235]	Prospective observational hospital study	European	Hospital Clinic of Barcelona/Spain	Jan 2000 - Dec 2005	≥18yrs (39·7±7·8)	73·7 [119]	11 UAT	≥19yrs 3,889,692 (2001)	161	0·7	6·8 [11] Lp	0·1
8	de Roux 2004[236]	Retrospective (cohort) study	European	Hospital Clinic of Barcelona/Spain	Oct 1996-Feb 2001	≥18yrs (68±18)	65·9 [893]	1 serology	≥18yrs, 3,590,000 (1991)	1356	8·4	0·1 [1] Lp	0·01
9	Diederen 2009[237]	Prospective hospital study	European	Alkmaar/The Netherlands	Dec 1998- Nov 2000	≥18yrs	¤NR	11 PCR	92,977 (2000)	242	130·2	4·5 [11] Lp	5·9
10	Endeman 2011[238]	Cohort hospital study	European	Nieuwegein/The Netherlands	Oct 2004-Aug 2006	NAR¤ 63.7±17	62 [124]	9 UAT	~61,087 (2008)	201	329·0	4·5 [9] Lp	14·7
11	Espana 2012[239]	Prospective observational study	European	Basque country/Spain	May 2006-Jun 2007	¤NAR (53·4 ±18·8)	57·3 [197]	*NR serology, culture, UAT	300,000 (published)	344	114·7	1·5 [5] Lp	1·7
12	Ewig 2002[240]	Prospective hospital study	European	Hospital Clinic of Barcelona/Spain	2-year period (actual yrs not stated)	NAR¤	60·9 [50/82]	11 serology and UAT	1,503,884 (2001)	204	13·6	5·4 [11] Lp	0·7
13	Fernández-Sabé 2003[241]	Prospective hospital study	European	Hospital Clinic of Barcelona/Spain	Feb 1995-Jul 2001	NAR¤ (mean <80yrs 60 (16–79); mean ≥ 80yrs 85 (80–97))	69·9 [1030]	NR* culture, serology, UAT	1,100,000 (published)	1474	23·9	9·1 [100] Lp	1·6
14	Ferrer 2018[242]	Prospective (observational) hospital study	European	Hospital Clinic of Barcelona/Spain	Jan 2000-Dec 2011	All ages	66·9 [337]	25 UAT	1,611,013 (2011 census)	664	41·2	3·8 [25] Lp	1·6
15	Garcia-Vidal 2009[243]	Prospective hospital study	European	Hospital Clinic of Barcelona/Spain	Jan 1995-Dec 2005	NAR¤ (70·96±13·8 (recurrent CAP)	68·6 [1067]	NR* serology, UAT	1 100 000 (published)	1556	12·9	0·3 [4] Lp (recurrent CAP)	0·04
16	Gómez-Junyent 2014[244]	Prospective (cohort) study	European	Hospital Clinic of Barcelona/Spain	Feb 1995-Oct 2011	≥18yrs	68·0 [2802]	NR* culture; UAT	≥18 yrs 3,590,000 (1991)	4121	6·8	5·5 [225] Lp	0·4

No.	Study Reference				Patient and Study Characteristics				CAP Results			*Legionella* results	
	First Author, Year, Ref	Study Design	WHO Region	City or Region/Country	Study period (for patient recruitment)	Age group (median or mean (±SD), range)	Male CAP patients (%) [no.]	Diagnostic method used to detect *Legionella*	Estimated (~) Study Population (Year)	No. of patients with CAP (that meet the criteria)	CAP incidence (annual)	Aetiology of CAP (%) [no.] of patients	*Legionella* incidence (annual) of CAP
17	Kothe 2008[245]	Prospective (observational) study	European	Multicentre/Germany (CAPNETZ)	Mar 2003-Oct 2005	All ages (<65 yrs, 47·2±12·7; ≥65 yrs 77·1±7·5)	56·1 [1486]	UAT, culture, PCR	~82,689,000 (2005)	2647	3·2	17·0 [451] NB specific *Legionella* spp. not recorded	0·5
18	Lérida 2000[246]	Prospective hospital study	European	Hospital Universitari de Bellvitge, Barcelona/Spain	Feb 1995-May 1997	NAR¤ (mean 59·2; range 22-86)	¤NR	2 serology	1,100,000 (published)	533	48·5	4·1 [22] Lp	2·0
19	Liapikou 2012[247]	Prospective hospital study	European	Barcelona/Spain	2004-2008	All ages (70±17)	59·9 [826]	NR* serology, culture, UAT	1, 579, 000 (2004)	1379	17·5	3·2 [44] Lp	0·6
20	Marcos 2006[248]	Prospective hospital study	European	Barcelona/Spain	Jan 2003-Mar 2004	≥14yrs (median 70)	58·0 [115]	NR* culture, serology, UAT, PCR	≥15yrs 1,212,131 (1991)	198	16·3	3·0 [6] Lp	0·5
21	Meijvis 2011[249]	Prospective study	European	Nieuwegein and Ede/The Netherlands	Nov 2007-Sep 2010	≥18yrs	56·3 [171]	NR* culture; UAT; PCR	~62,600 (Nieuwegein);105,495 (Ede) (2004)	304	60·1	3·9 [12] NB specific *Legionella* spp. not recorded	2·4
22	Müller 2007[250]	Prospective hospital (randomized) study	European	Basel/Switzerland	Dec 2002-Feb 2005	≥18yrs	84·2 [314]	1 culture; 4 UAT	~ >19yrs 132,285 (2001)	373	134·3	1·3[5] Lp	1·8
23	Mykietiuk 2005[251]	Prospective study	European	Hospital Universitari de Bellvitge, Barcelona/Spain	Feb 1995-Dec 2003	All ages	¤NR	120 UAT; 80 serology; 43 culture	1,100,000 (published)	1934	19·5	7·2 [139] Lp	1·4
24	Perello 2010[252]	Retrospective hospital study	European	Barcelona/Spain	Mar 2005-Feb 2008	All ages (43 ±10)	65·3 [77]	2 UAT	500,000 (published)	118	7·9	1·7 [2] NB specific *Legionella* spp. not recorded	0·1
25	Polverino 2015[253]	Prospective (observational) study	European	Hospital Clinic of Barcelona/Spain	2000-2011	All ages	60·6 [2118]	NR	1,503,884 (2001)	3495	19·4	3·2 [112] Lp	0·6
26	Raeven 2016[254]	Prospective study	European	Alkmaar; Nieuwegein/The Netherlands	Dec 1998-Nov 2000; Oct 2004-Aug 2006	≥18yrs (median 67)	57·2 [561]	NR	~93,840 Alkmaar; ~62,600 Nieuwegein (2004)	980	156·6	4·3 [42] NB specific *Legionella* spp. not recorded	6·7
27	Rosón 2004[255]	Prospective hospital study	European	Hospital Universitari de Bellvitge, Barcelona/Spain	Feb 1995-Dec 2000	All ages (65·8yr ± 16·9 (early response) 59·5yr ± 16·8 early failure)	69·8 [932]	61 UAT; 16 culture; 15 serology	1 100 000 (published)	1335	20·9	6·9 [92] Lp	1·5
28	Ruiz 1999[256]	Prospective hospital study	European	Hospital Clinic of Barcelona/Spain	Oct 1996-Dec 1997	≥60yrs	65·8 [260]	15 serology; 2 UAT	≥60yrs = 890,257 (1991)[7]	395	36·9	4·3 [17] Lp	1·6
29	Simonetti 2014[257]	Prospective (observational) study	European	Hospital Universitari de Bellvitge , Barcelona/Spain	Jan 2003-Dec 2012	All ages	68·5 [1493]	NR* serology; culture; UAT	~1,496,000 (2000)	2179	15·3	4·7 [102] Lp	0·7
30	Sorde´2011[258]	Prospective study	European	Hospital Clinic of Barcelona/Spain	Feb 2007- Jan 2008	≥16yrs	66·9 [317]	14 UAT; NR* serology; culture; PCR	500,000 (published)	474	94·8	3·2 [15] Lp	3
31	Taylor 2013[259]	Prospective observational hospital study	European	Edinburgh/Scotland	2005-2009	≥18yrs	49·9 [673]	NR	407,804 (2003)	1348	66·1	1·3 [18] Lp	0·4
32	van Mens 2011[260]	Prospective hospital study	European	Nieuwegein/The Netherlands	Oct 2004-Aug 2006; Nov 2007-Jan 2009	≥18yrs (mean 63)	59·3 [207]	NR UAT, culture, PCR, serology	47,120 ≥19 yrs (2003)	349	148·1	4·3 [15] NB specific *Legionella* spp. not recorded	6·4
33	Vazquez 2003[261]	Prospective (cohort) study	European	Hospital Clinic of Barcelona/Spain	Oct 1996-Oct 2000	≥14yrs	¤NR	NR* culture, serology, UAT	~1,329,257 (1996; ≥15yrs))	1222	30·0	2·5 [30] Lp	0·6
34	Viasus 2013[262]	Prospective hospital study	European	Hospital Clinic of Barcelona/Spain	Feb 1995-Dec 2010	NAR¤ median 70yrs (IQR 50-79)	68·4 [2691]	194 UAT; 94 serology; 49 culture	1,503,884 (2001)	3934	45·1	5·5 [215] (1 *L. longbeachae*; 214 Lp)	2·5

[7] http://www.ine.es/jaxi/tabla.do

No.	Study Reference		Patient and Study Characteristics						CAP Results			*Legionella* results	
	First Author, Year, Ref	Study Design	WHO Region	City or Region/Country	Study period (for patient recruitment)	Age group (median or mean (±SD), range)	Male CAP patients (%) [no.]	Diagnostic method used to detect *Legionella*	Estimated (~) Study Population (Year)	No. of patients with CAP (that meet the criteria)	CAP incidence (annual)	Aetiology of CAP (%) [no.] of patients	*Legionella* incidence (annual) of CAP
35	Viasus 2011[263]	Prospective (cohort) hospital study	European	Hospital Clinic of Barcelona/Spain	Feb 1995-Dec 2008	NAR¤ age (mean 61·8±13yrs for patients with liver cirrhosis; 66·8±16·9yrs)	¤NR	NR* UAT, culture, serology	1,503,884 (2001)	3420	227·4	6·1 [207] Lp	13·8
36	Viasus 2011[264]	Prospective (cohort) hospital study	European	Hospital Universitari de Bellvitge , Barcelona/Spain	Feb 1995-Apr 2010	NAR¤ age, median yrs 77 (67–84) CKD; 70 (56–79) non-CKD	68·5 [2602]	NR UAT, serology	1,332,928 ≥15yrs (1996)	3800	6·2	5·6 [213] Lp	3·5
37	Lieberman 2003[265]	Prospective hospital study	Eastern Mediterranean	Beer-Sheva/Israel	Nov 1998- Mar 1999	NAR¤ (75·0±6·6; range 65–92)	50·3 [67]	9 serology	753,500 (1995)	133	17·7	6·8 [9] NB specific *Legionella* spp. not recorded	1·2
38	Ishida 2004[266]	Prospective hospital study	Western Pacific	Kurashiki/Japan	Feb 2001-Jan 2004	≥15yrs (65·3±18·3; range 16–99)	65·3 [228]	NR*serology; UAT	433,477 (2003)	349	26·8	1·4 [5] NB specific *Legionella* spp. not recorded	0·4
39	Ishida 2000[267]	Prospective hospital study	Western Pacific	Kurashiki/Japan	Jul 1994-Jun 1999	¤NAR 64·4 +-17·5yrs	75·6 [408]	2 serology; 2 UAT	453,618 (1995)	540	23·8	0·7 [4] 3 Lp; 1 *L. micdadei*	0·2
40	Ishida 1998[268]	Prospective hospital study	Western Pacific	Kurashiki/Japan	Jul 1994-Jun 1997	≥15yrs (65·2 ± 16·3; range; 18-93)	72·0 [229]	0 culture; 2 serology	453,618 (1995)	318	23·4	0·6 [2] 1 Lp; 1 *L. micdadei*	0·1
41	Takayanagi 2006[269]	Prospective hospital study	Western Pacific	Kumagaya (Saitama) /Japan	1999-2004	NAR¤ (range 15-94)	70·2 [417]	NR* culture; UAT	5,900,445 (≥15yrs) (2000)	594	1·7	1·9 [11] Lp	0·03
42	Zhou 2009[270]	Retrospective hospital study	Western Pacific	Beijing/China	2000-2005	NAR¤ (43·0+19·8)	61·0 [144]	3 culture	13,569,194 (2000)	236	0·3	1·3 [3] Lp	0·003
43	Luna 2000[271]	Prospective hospital study	The Americas	Buenos Aires/Argentina	1997-1998	≥17yrs	46·5 [161]	NR* culture; serology; UAT	2,508,000 (1998; ≥15yrs)	346	5·7	1·2 [4] Lp	0·1

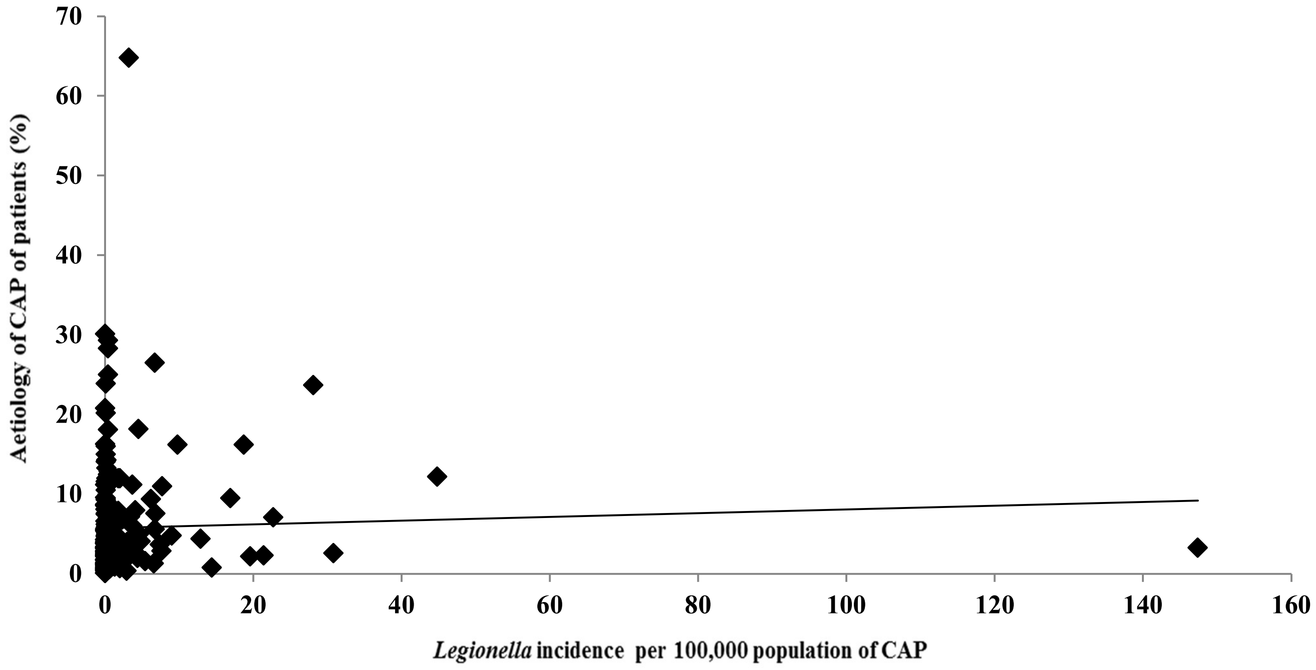

Figure S3. *Legionella* incidence of 219 CAP studies which recruited patients from 1 January 1990.

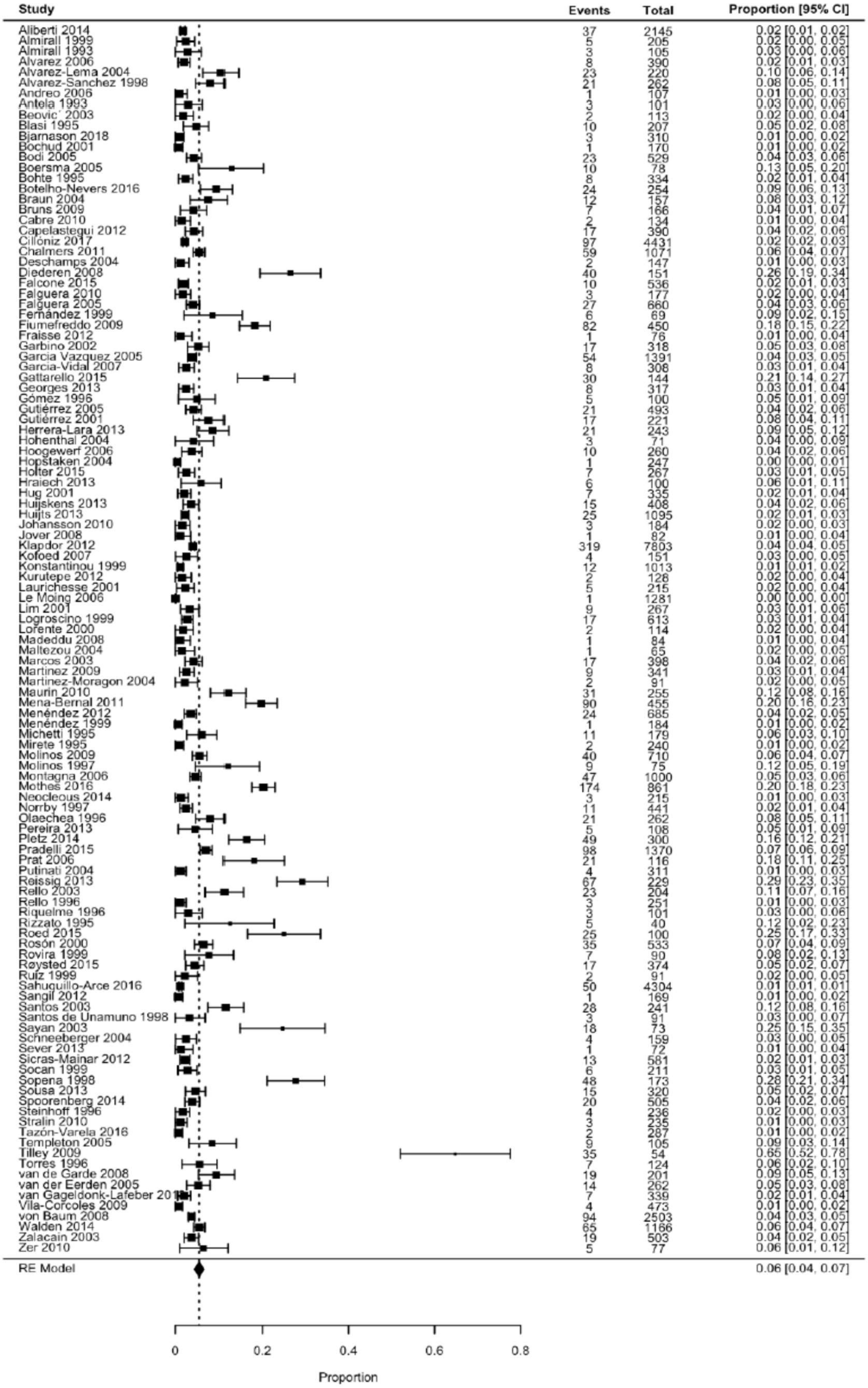

Figure S4. Forest plot of European studies showing proportions of *Legionella* as the causative agent to CAP.

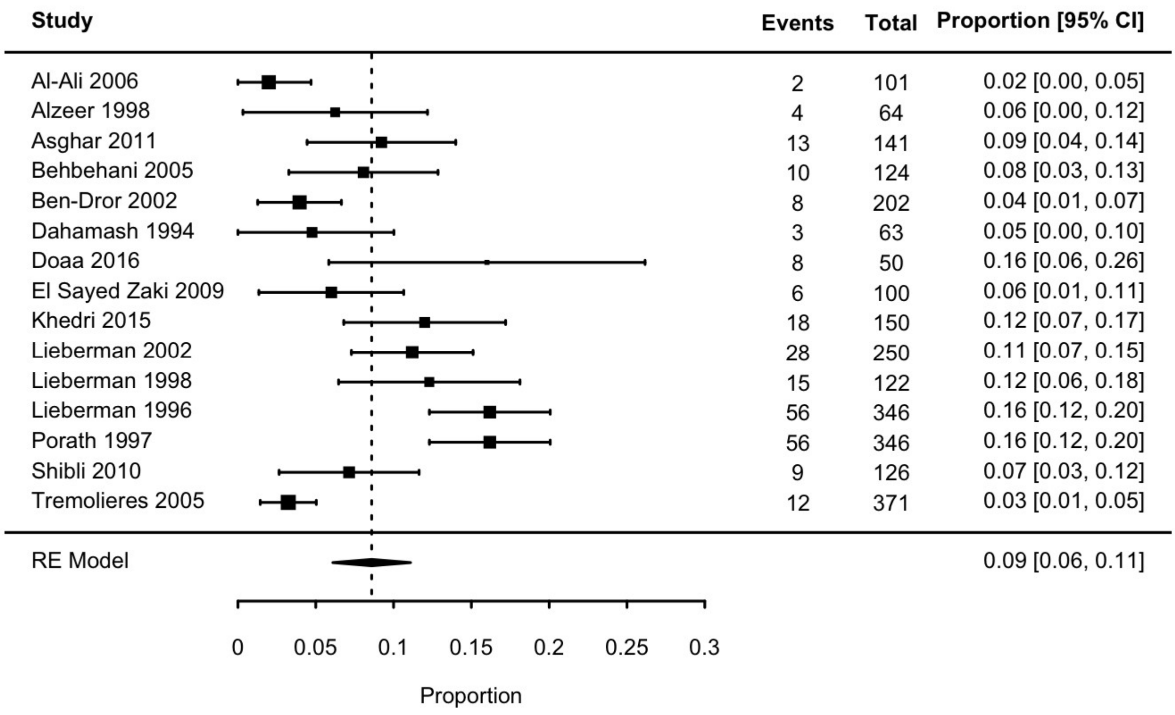

Figure S5. Forest plot of Eastern Mediterrean studies showing proportions of *Legionella* as the causative agent to CAP.

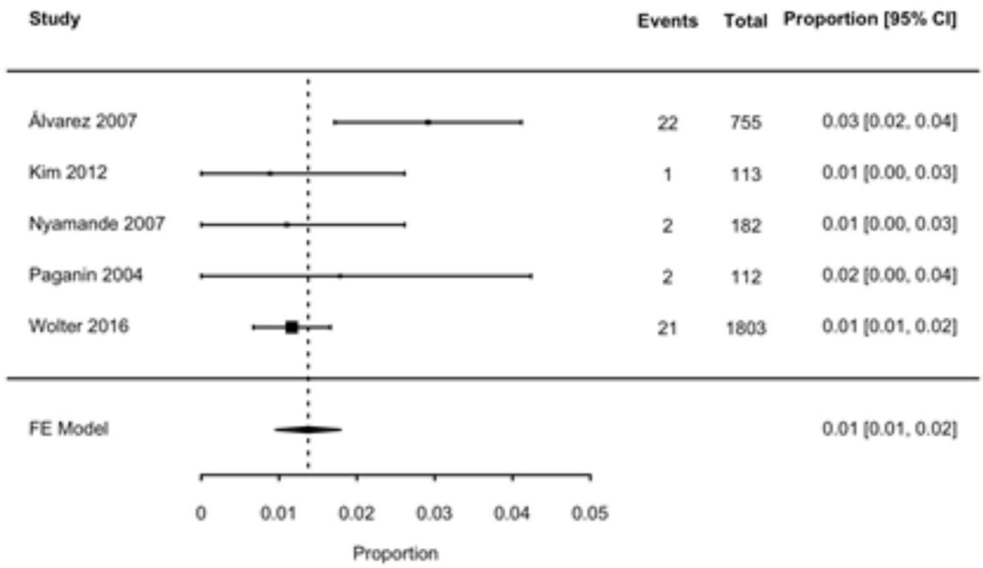

Figure S6. Forest plot of African studies showing proportions of *Legionella* as the causative agent to CAP.

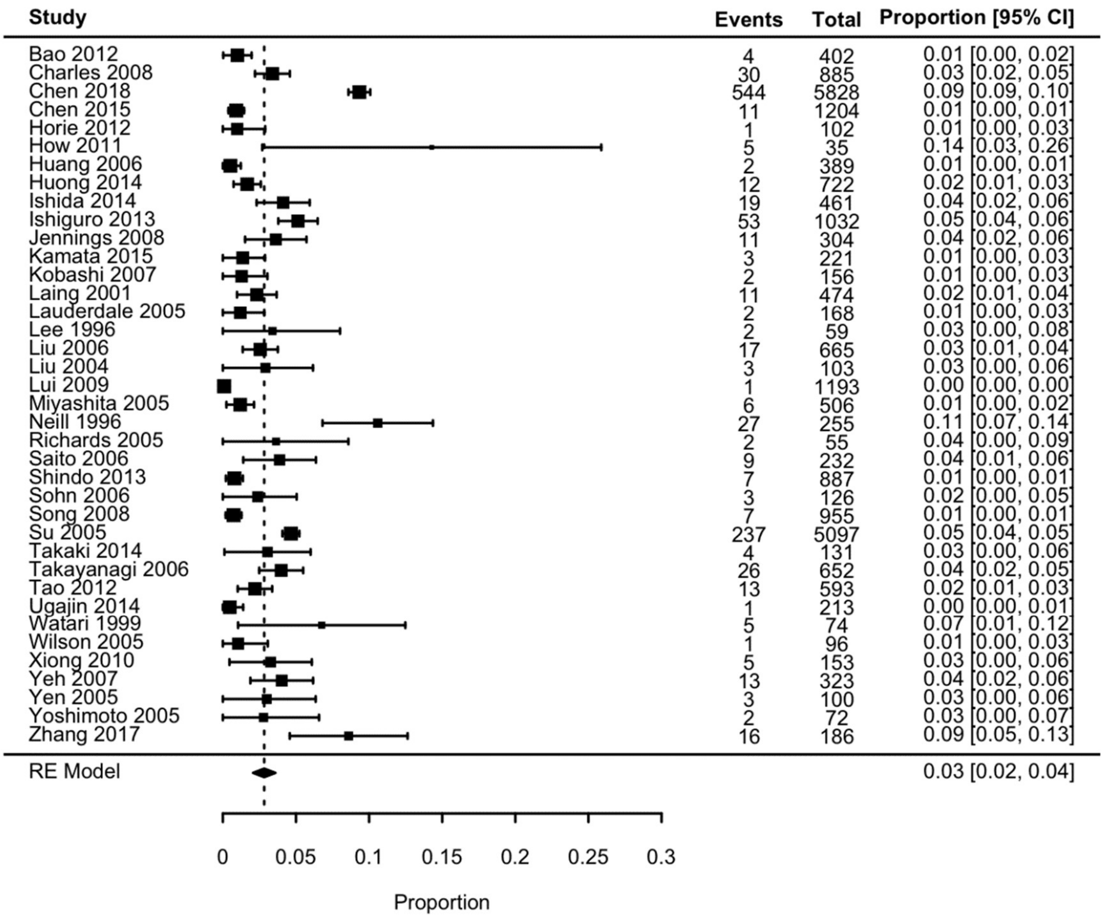

Figure S7. Forest plot of Western Pacific studies showing proportions of *Legionella* as the causative agent to CAP

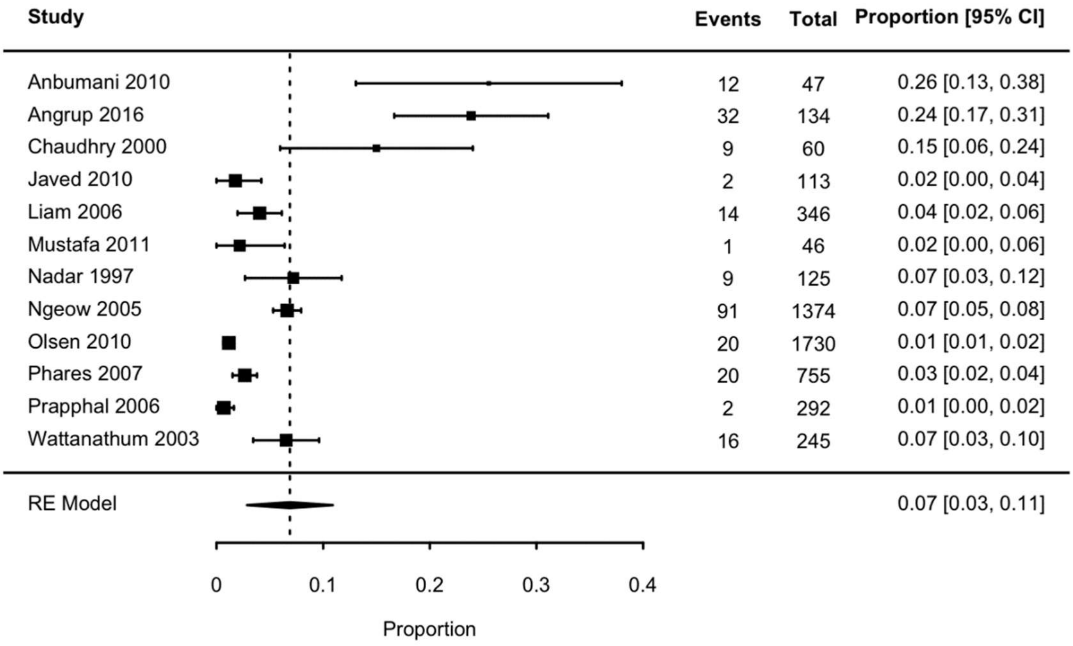

Figure S8. Forest plot of South-East Asian studies showing proportions of *Legionella* as the causative agent to CAP.

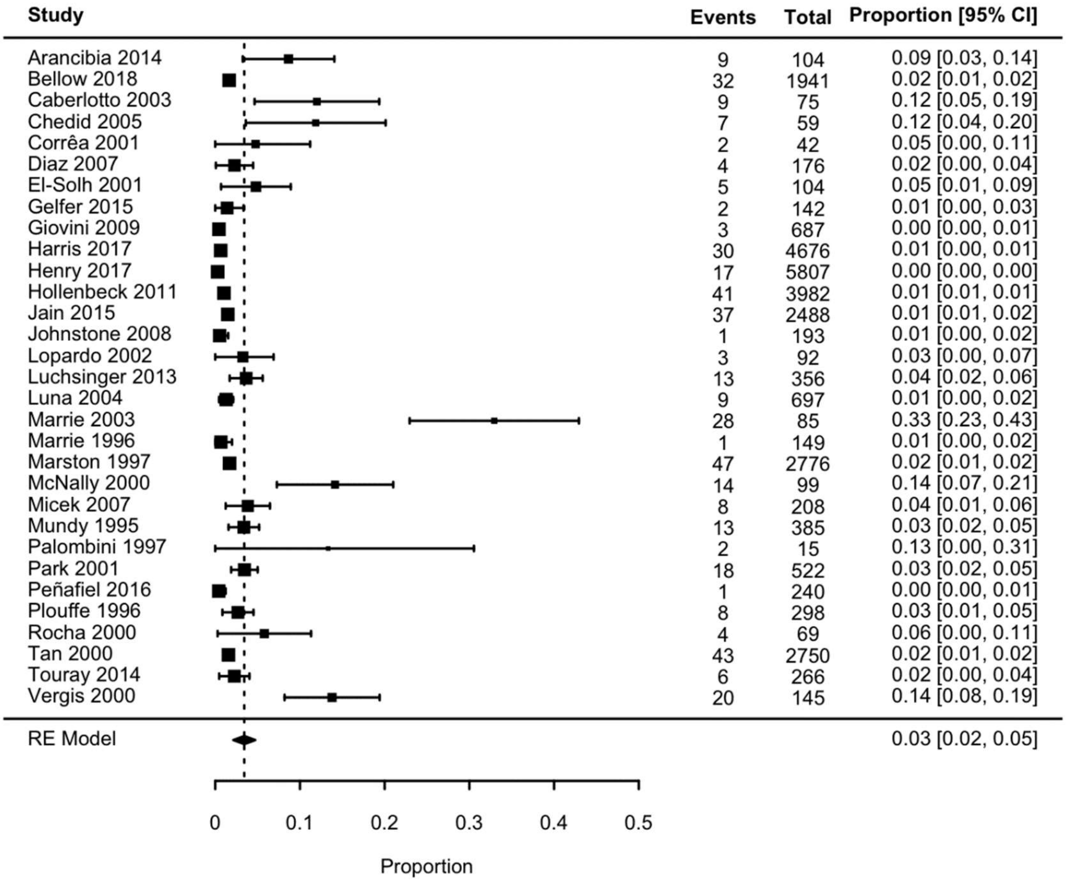

Figure S9. Forest plot of The Americas studies showing proportions of *Legionella* as the causative agent to CAP.

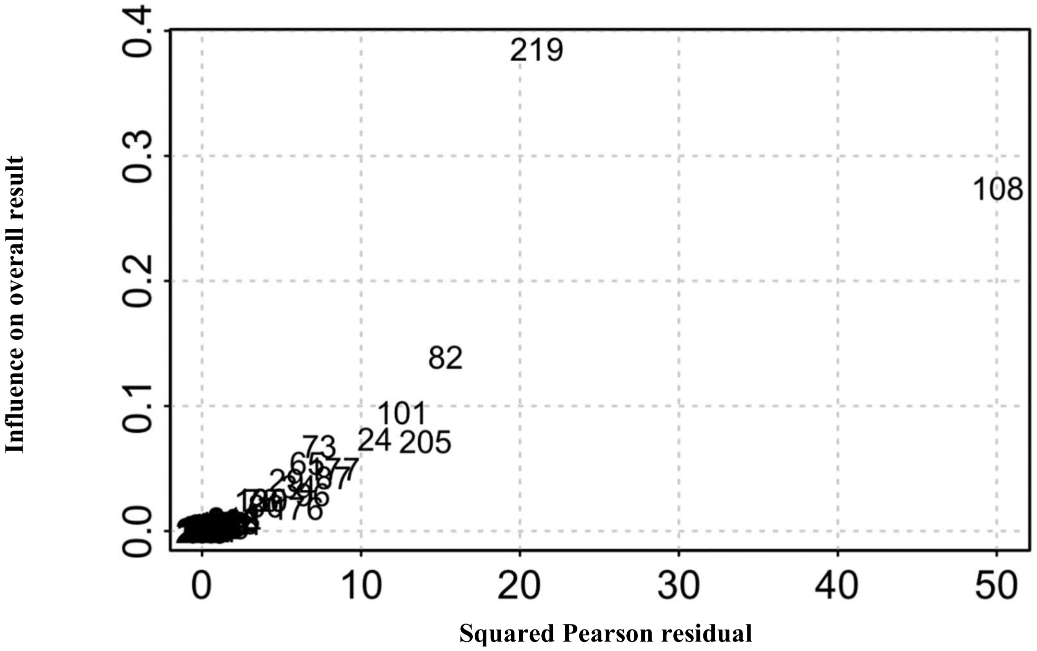

Figure S10. Baujat plot showing the contribution of each study to the overall Q-statistics for heterogeneity on the horizontal axis verses the influence of each study on the vertical axis. The influence of each study is defined as the standardized squared difference between the overall estimate based on a fixed-effects model. The numbers refer to the studies listed in Table S1.

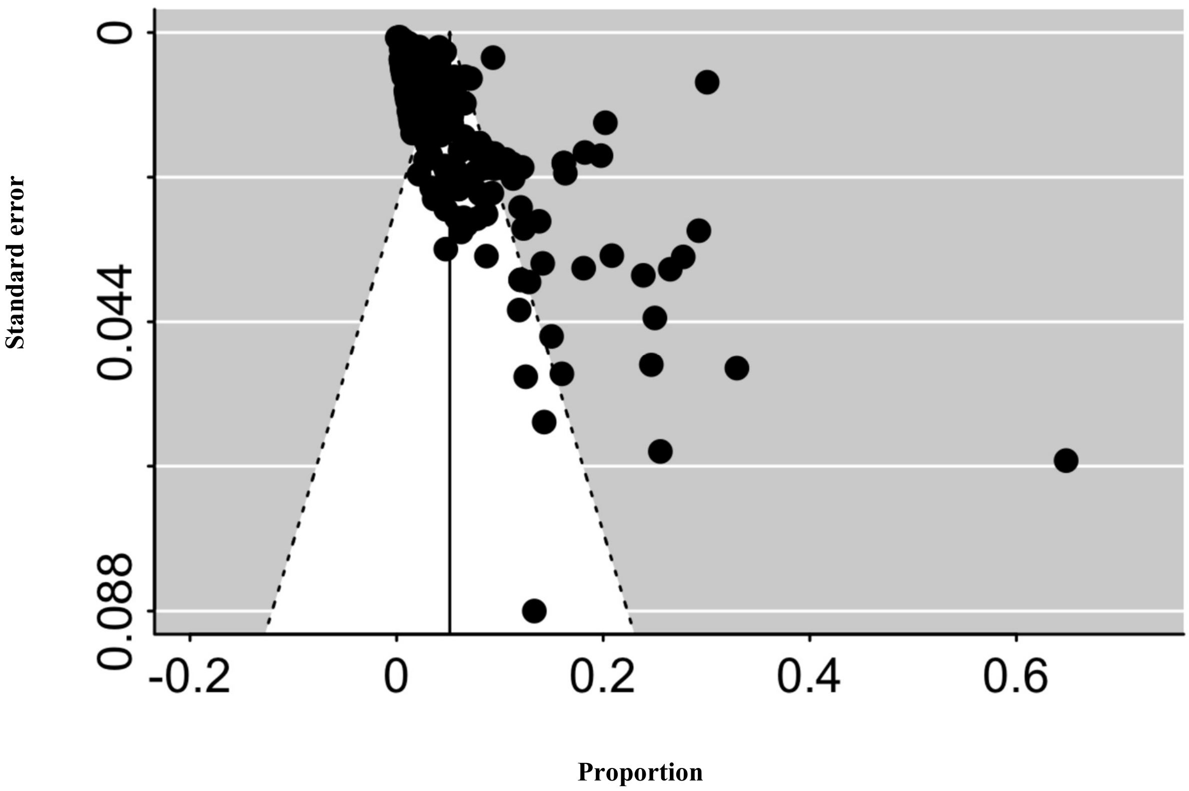

Figure S11. Funnel plot to evaluate publication bias (all studies)

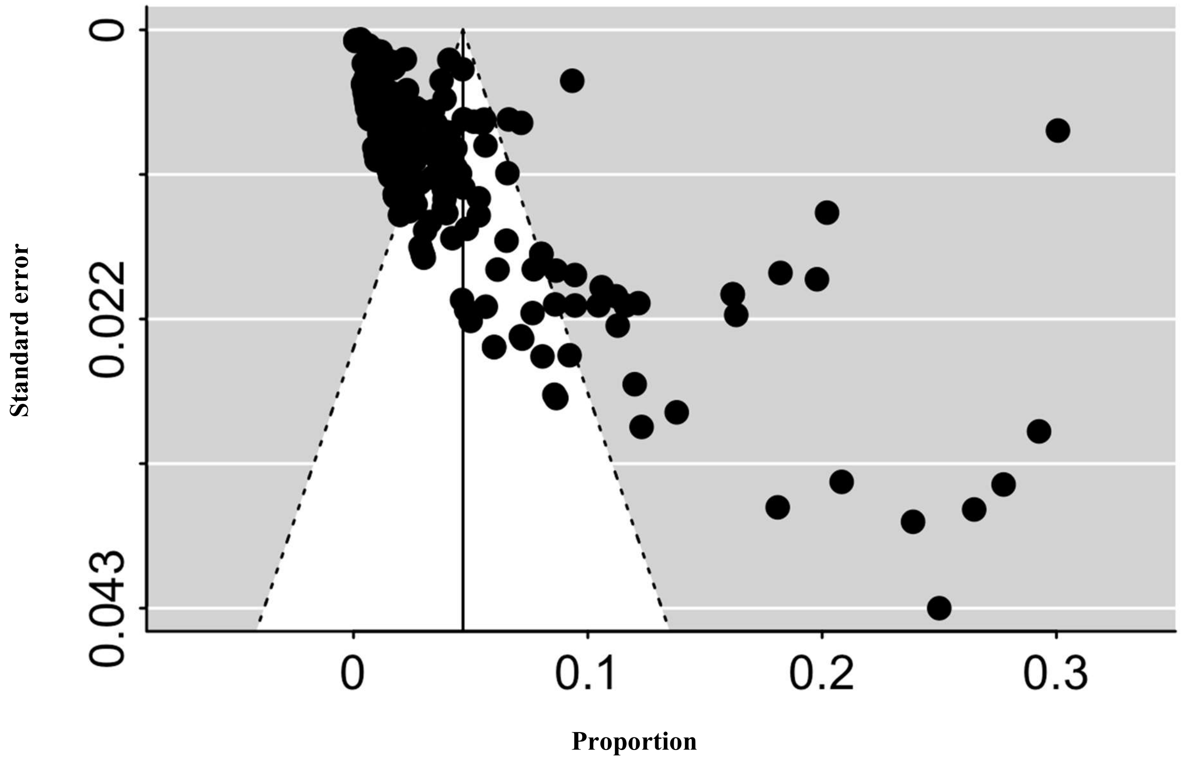

Figure S12. Funnel plot (Trim and Fill)

REFERENCES FOR THE SUPPLEMENTARY MATERIAL

1. Aliberti, S.; Brambilla, AM.; Chalmers, JD.; Cilloniz, C.; Ramirez, J.; Bignamini, A.; Prina, E.; Polverino, E.; Tarsia, P.; Pesci, A.; Torres. A. Phenotyping community-acquired pneumonia according to the presence of acute respiratory failure and severe sepsis. *Respir. Res*. **2014**, 15, 1–10.
2. Almirall, J.; Bolibar, I.; Balanzo, X.; Gonzalez, CA. Risk factors for community-acquired pneumonia in adults: a population-based case-control study. *Eur. Respir. J.* **1999**, 13, 349–355.
3. Almirall, J.; Morato, I.; Riera, F.; Verdaguer, A.; Priu, R.; Coll, P.; Vidal, J.; Murgui, L.; Valls, F.; Catalan, F. Incidence of community-acquired pneumonia and Chlamydia pneumoniae infection: a prospective multicentre study. *Eur. Respir. J.* **1993**, 6, 14–18.
4. Álvarez, RF.; Toste, IS.; Cuadrado, GR.; Gonzálvez, AM.; Blanco, JAG.; Martín, IG. Treatment and course of community-acquired pneumonia caused by atypical pathogens. *Arch. Bronconeumol.* **2006**, 42, 430–433.
5. Alvarez-Lerma, F.; Palomar, M.; Olaechea, P.; León, C.; Sanchez, M.; Bermejo, B.; Bermejo, B. Levofloxacin in the treatment of pneumonia in intensive care unit patients. *J. Chemother.* **2004**, 16, 549–556.
6. Álvarez-Sánchez, B.; Álvarez-Lerma, F.; Jordà, R.; Serra, J.; López-Cambra, MJ.; Sandar, MD. Prognostic factors and etiology of patients admitted to an intensive care unit with severe community acquired pneumonia. A Spanish multicenter study. *Factores pronósticos y etiología de los pacientes con neumonía comunitaria grave ingresados en UCI Estudio multicéntrico Español.* **1998**, 111, 650–654.
7. Andreo, F.; Domínguez, J.; Ruiz, J.; Blanco, S.; Arellano, E.; Prat, C.; Morera, J.; Ausina, V. Impact of rapid urine antigen tests to determine the etiology of community-acquired pneumonia in adults. *Respir. Med.* **2006**, 100, 884–891.
8. Antela, A.; Guerrero, A.; Meseguer, M.; Gonzalez-Sainz, J,. Escudero, R.; Perez-Elias, MJ.; Quereda, C.; Hermida, JM.; Cobo, J.; Montilla, P.; Fortun, J.; Buzon, E. [Community-acquired pneumonia: prospective study of 101 adult, immunocompetent patients for 1 year]. *Enferm. Infecc. Microbiol. Clin.* **1993**, 11, 525–530.
9. Beovic, B.; Bonac, B.; Kese, D.; Avšič-Županc, T.; Kreft, S.; Lesničar, G.; Gorišek-Reberšek, J.; Rezar, L.; Letonja, S. Aetiology and clinical presentation of mild community-acquired bacterial pneumonia. *Eur. J. Clinical. Microbiol. Infect. Dis.* **2003**, 22, 584–591.
10. Bjarnason, A.; Westin, J.; Lindh, M.; Andersson, LM.; Kristinsson, KG.; Löve, A.; Baldursson, O.; Gottfredsson, M. Incidence, Etiology, and Outcomes of Community-Acquired Pneumonia: A Population-Based Study. *Open Forum Infect. Dis.* **2018**, 5, ofy010.
11. Blasi, F.; Cosentini, R.; Raccanelli, R.; Rinaldi, A.; Denti, F.; Loprete, F.; Allegra, L. Emerging pathogens of community-acquired pneumonia: a two-year prospective study. *J. Chemother.* **1995**, 7, 115–116.
12. Bochud, PY.; Moser, F.; Erard, P.; Verdon, F.; Studer, JP.; Villard, G.; Cosendai, A.; Cotting, M.; Heim, F.; Tissor J.; Strub, Y. Community-acquired pneumonia. A prospective patient study. *Medicine*. **2001**, 80, 75–87.
13. Bodi, M.; Rodriguez, A.; Sole-Violan, J.; Gilavert, MC.; Garnacho, J.; Blanquer, J.; Jimenez, J.; De La Torre, MV.; Sirvent, JM.; Almirall, J.; Doblas, A. Antibiotic prescription for community-acquired pneumonia in the intensive care unit: impact of adherence to Infectious Diseases Society of America guidelines on survival. *Clin. Infect. Dis.* **2005**, 41, 1709–1716.
14. Boersma, WG.; Sportel, JH.; Lowenberg, A.; Koeter, GH. High Prevalence of Obstructive Airways Disease in Hospitalized Patients with Community-Acquired Pneumonia: Comparison of Four Etiologies. *Clin. Pulm. Med.* **2005**, 12, 291–296.
15. Bohte, R.; van Furth, R.; van den Broek, PJ. Aetiology of community-acquired pneumonia: a prospective study among adults requiring admission to hospital. *Thorax.* **1995**, 50, 543–547.
16. Botelho-Nevers, E.; Grattard, F.; Viallon, A.; Allegra, S.; Jarraud, S.; Verhoeven, P.; Marcuccilli, A.; Lucht, F.; Pozzetto, B.; Berthelot, P. Prospective evaluation of RT-PCR on sputum versus culture, urinary antigens and serology for Legionnaire's disease diagnosis. *J. Infect.* **2016**, 73, 123–128.
17. Braun, J.; De Graaff, C.; De Goey, J.; Zwinderman, A.; Petit, P. Buiten het ziekenhuis opgelopen pneumonie: verwekkers en beloop bij patiënten opgenomen in een algemeen ziekenhuis. *Ned tijdschr Geneeskd.* **2004**, 148, 836–839.
18. Bruns, AH.; Oosterheert, JJ.; Hustinx, WN.; Gaillard, CA.; Hak, E.; Hoepelman, AI. Time for first antibiotic dose is not predictive for the early clinical failure of moderate–severe community-acquired pneumonia. *Eur. J. Clinical. Microbiol. Infect. Dis.* **2009**, 28, 913–919.
19. Cabre, M.; Serra-Prat, M.; Palomera, E.; Almirall, J.; Pallares, R.; Clavé, P. Prevalence and prognostic implications of dysphagia in elderly patients with pneumonia. *Age and Ageing*. **2010**, 39, 39–45.

20. Capelastegui, A.; España, PP.; Bilbao, A.; Gamazo, J.; Medel, F.; Salgado, J.; Gorostiaga, I.; de Goicoechea, MJ.; Gorordo, I.; Esteban, C.; Altube, L. Etiology of community-acquired pneumonia in a population-based study: link between etiology and patients characteristics, process-of-care, clinical evolution and outcomes. *BMC Infect. Dis.* **2012**, 12, 1–9.
21. Cilloniz, C.; Ewig, S.; Gabarrus, A.; Ferrer, M. Seasonality of pathogens causing community-acquired pneumonia. *Respirology.* **2017**, 22, 778–785.
22. Chalmers, JD.; Taylor JK.; Singanayagam, A.; Fleming, GB.; Akram, AR.; Mandal, P.; Choudhury, G.; Hill, AT. Epidemiology, antibiotic therapy, and clinical outcomes in health care–associated pneumonia: a UK cohort study. *Clin. Infect. Dis.* **2011**, 53, 107–113.
23. Deschamps, C.; Lacombe, K.; Lalande, V.; Meyohas, M.; Girard, P.; Meynard, J. The use of microbiological tools for the diagnosis of nosocomial pulmonary infections. *Presse. Medicale.* **2004**, 33, 1505–1510.
24. Diederen, BM.; Kluytmans, JA.; Vandenbroucke-Grauls, CM.; Peeters, MF. Utility of real-time PCR for diagnosis of Legionnaires' disease in routine clinical practice. *J. Clin. Microbiol.* **2008**, 46, 671–677.
25. Falcone, M.; Russo, A.; Giannella, M.; Cangemi, R.; Scarpellini, MG.; Bertazzoni, G.; Alarcón, JM.; Taliani, G.; Palange, P.; Farcomeni, A.; Vestri, A. Individualizing risk of multidrug-resistant pathogens in community-onset pneumonia. *PLoS One.* **2015**, 10, e0119528.
26. Falguera, M.; Ruiz-González, A.; Schoenenberger, JA.; Touzon, C.; Gázquez, I.; Galindo, C.; Porcel, JM. Prospective, randomised study to compare empirical treatment versus targeted treatment on the basis of the urine antigen results in hospitalised patients with community-acquired pneumonia. *Thorax.* **2010**, 65, 101–106.
27. Falguera, M.; Pifarre, R.; Martin, A.; Sheikh, A.; Moreno, A. Etiology and outcome of community-acquired pneumonia in patients with diabetes mellitus. *Chest.* **2005**, 128, 3233–3239.
28. Fernández, AF.; Batlle, M.; Ribera, J.; Matas, L.; Rosell, A.; Manzano, JL.; Flores, A.; Feliu, F. Pneumonias in patients with malignant hemopathies. Their etiology, response to treatment and prognostic factors in 69 patients (88 episodes). *Med. Clin.* **1999**, 112, 321–325.
29. Fiumefreddo, R.; Zaborsky, R.; Haeuptle, J.; Christ-Crain, M.; Trampuz, A.; Steffen, I.; Frei, R.; Müller, B.; Schuetz, P. Clinical predictors for Legionella in patients presenting with community-acquired pneumonia to the emergency department. *BMC Pulm. Med.* **2009**, 9: 1–9.
30. Fraisse, T.; Fayad, G.; Jardy, C.; Sotto, A. Evaluation of empirical antibiotherapy for acute community-acquired pneumonia prescribed in emergency departments. *Méd. Mal. Infect.* **2012**, 42, 5–9.
31. Garbino, J.; Sommer, R.; Gerber, A.; Regamey, C.; Vernazza, P.; Genne, D.; Dür, P.; Rothen, M.; Unger, JP.; Lew, D. Prospective epidemiologic survey of patients with community-acquired pneumonia requiring hospitalization in Switzerland. *Int. J. Infect. Dis*. **2002**, 6, 288–293.
32. Garcia Vazquez, E.; Mensa, J.; Martinez, JA.; Marcos, MA.; Puig, J.; Ortega, M.; Torres, A. Lower mortality among patients with community-acquired pneumonia treated with a macrolide plus a beta-lactam agent versus a beta-lactam agent alone. *Eur. J. Clin. Microbiol. Infect. Dis.* **2005**, 24, 190–195.
33. Garcia-Vidal, C.; Calbo, E.; Pascual, V.; Ferrer, C.; Quintana, S.; Garau, J. Effects of systemic steroids in patients with severe community-acquired pneumonia. *Eur. Respir. J.* **2007**, 30, 951–956.
34. Gattarello, S.; Lagunes, L.; Vidaur, L.; Solé-Violán, J.; Zaragoza, R.; Vallés, J.; Torres, A.; Sierra, R.; Sebastian, R.; Rello, J. Improvement of antibiotic therapy and ICU survival in severe non-pneumococcal community-acquired pneumonia: a matched case–control study. *Crit. Care.* **2015**, 19, 1–2.
35. Georges, H.; Journaux, C.; Devos, P.; Alfandari, S.; Delannoy, PY.; Meybeck, A.; Chiche, A.; Boussekey, N.; Leroy, O. Improvement in process of care and outcome in patients requiring intensive care unit admission for community acquired pneumonia. *BMC Infect. Dis.* **2013**, 13, 1–10.
36. Gomez, J.; Banos, V.; Gómez, JR.; Soto, MC.; Munoz, L.; Nunez, ML.; Canteras, M.; Valdes, M. Prospective study of epidemiology and prognostic factors in community-acquired pneumonia. *Eur. J. Clin. Microbiol. Infect. Dis.* **1996**, 15, 556–560.
37. Gutierrez, F.; Masia, M.; Rodriguez, JC.; Mirete, C.; Soldán, B.; Padilla, S.; Hernández, I.; De Ory, F.; Royo, G.; Hidalgo, AM. Epidemiology of community-acquired pneumonia in adult patients at the dawn of the 21st century: a prospective study on the Mediterranean coast of Spain. *Clin. Microbiol. Infect.* **2005**, 11, 788–800.
38. Gutierrez, FJA.; del Castillo Otero, D.; Garcia Fernandez, A.; Romero, BR.; del Rey Pérez, JJ.; Campos, GS.; Gómez, JC. [Prospective study of 221 community acquired pneumonias followed up in an outpatient clinic. Etiology and clinical-radiological progression]. *Med. Clin.* **2001**, 116, 161–166.

39. Herrera-Lara, S.; Fernández-Fabrellas, E.; Cervera-Juan Á.; Blanquer-Olivas, R. Do seasonal changes and climate influence the etiology of community acquired pneumonia? *Arch. Bronconeumol*. **2013**, 49, 140–145.
40. Hohenthal, U.; Vainionpää, R.; Meurman, O.; Vahtera, A.; Katiskalahti, T.; Nikoskelainen, J.; Kotilainen, P. et al. Aetiological diagnosis of community acquired pneumonia: utility of rapid microbiological methods with respect to disease severity. *Scand. J. Infect. Dis*. **2008**, 4, 131–138.
41. Hoogewerf, M.; Oosterheert, JJ.; Hak, E.; Hoepelman, IM.; Bonten, MJ. Prognostic factors for early clinical failure in patients with severe community-acquired pneumonia. *Clin. Microbiol. Infect.* **2006**, 12, 1097–1104.
42. Hopstaken, R.; Witbraad, T.; Van Engelshoven, J.; Dinant, G. Inter-observer variation in the interpretation of chest radiographs for pneumonia in community-acquired lower respiratory tract infections. *Clin. Radiol.* **2004**, 59, 743–752.
43. Holter, JC.; Müller, F.; Bjørang, O.; Samdal, HH.; Marthinsen, JB.; Jenum, PA.; Ueland, T.; Frøland, SS;. Aukrust, P.; Husebye, E.; Heggelund, L. Etiology of community-acquired pneumonia and diagnostic yields of microbiological methods: a 3-year prospective study in Norway. *BMC Infect. Dis.* **2015**, 15, 1–11.
44. Hraiech, S.; Alingrin, J.; Dizier, S.; Brunet, J.; Forel, JM.; La Scola, B.; Roch, A.; Papazian, L.; Pauly, V. Time to intubation is associated with outcome in patients with community-acquired pneumonia. *PloS One.* **2013**, 8: e74937.
45. Hug, BL.; Rossi, M. A year's review of bacterial pneumonia at the central hospital of Lucerne, Switzerland. *Swiss. Med. Wkly*. **2001**, 131, 687–692.
46. Huijskens, EG.; Koopmans, M.; Palmen, FM.; van Erkel, AJ.; Mulder, PG.; Rossen, JW. The value of signs and symptoms in differentiating between bacterial, viral and mixed aetiology in patients with community-acquired pneumonia. J. *Med. Microbiol.* **2014**, 63, 441–452.
47. Huijts, SM.; Pride, MW.; Vos, JM.; Jansen, KU.; Webber, C.; Gruber, W.; Boersma, WG.; Snijders, D.; Kluytmans, JA.; van der Lee, I.; Kuipers, BA. Diagnostic accuracy of a serotype-specific antigen test in community-acquired pneumonia. *Eur. Respir. J.* **2013**, 42, 1283–1290.
48. Johansson, N.; Kalin, M.; Tiveljung-Lindell, A.; Giske, CG.; Hedlund, J. Etiology of community-acquired pneumonia: in-creased microbiological yield with new diagnostic methods. *Clin. Infect. Dis.* **2010**, 50, 202–209.
49. Jover, F.; Cuadrado, JM.; Andreu, L.; Martínez, S.; Cañizares, R.; de la Tabla, VO.; Martin, C.; Roig, P.; Merino, J. A comparative study of bacteremic and non-bacteremic pneumococcal pneumonia. *Eur. J. Intern. Med.* **2008**, 19, 15–21.
50. Klapdor, B.; Ewig, S.; Pletz, MW.; Rohde, G.; Schütte, H.; Schaberg, T.; Welte, T. Community-acquired pneumonia in younger patients is an entity on its own. *Eur. Respir. J.* **2012**, 39,1156–1161.
51. Kofoed, K.; Andersen, O.; Kronborg, G.; Tvede, M.; Petersen, J.; Eugen-Olsen, J.; Larsen, K. Use of plasma C-reactive protein, procalcitonin, neutrophils, macrophage migration inhibitory factor, soluble urokinase-type plasminogen activator receptor, and soluble triggering receptor expressed on myeloid cells-1 in combination to diagnose infections: a prospective study. *Crit. Care.* **2007**, 11, 1–10.
52. Konstantinou, G.; Bokolas, G.; Alexiou-Daniel, S.; Antoniadis, A. Infectious agents causing viral and atypical pneumonia in Northern Greece during the years 1992-1996. *Arch Hell Med* **1999**, 16, 595–601.
53. Kurutepe, S.; Ecemiş, T.; Ozgen, A.; Bicmen, C.; Celik, P.; Sürücüoğlu, S. Investigation of bacterial etiology with conventional and multiplex PCR methods in adult patients with community-acquired pneumonia. *Mikrobiyol. Bul.* **2012**, 46, 523–531.
54. Laurichesse, H.; Sotto, A.; Bonnet, E.; Abraham, B.; Neau, D.; Badiaga, S.; Gaillat, J.; Fabbro-Peray, P.; Infectio-Sud Study Group. Pre- and in-hospital management of community-acquired pneumonia in southern France, 1998-99. *Eur. J. Clin. Microbiol. Infect. Dis.* **2001**, 20, 770–778.
55. Le Moing, V.; Rabaud, C.; Journot, V.; Duval, X.; Cuzin, L.; Cassuto, JP.; Al Kaied, F.; Dellamonica, P.; Chêne, G.; Raffi, F.; APROCO Study Group. Incidence and risk factors of bacterial pneumonia requiring hospitalization in HIV-infected patients started on a protease inhibitor-containing regimen. *HIV Med.* **2006**, 7, 261–267.
56. Lim, WS.; Macfarlane, JT.; Boswell, TC.; Harrison, TG.; Rose, D.; Leinonen, M.; Saikku, P. Study of community acquired pneumonia aetiology (SCAPA) in adults admitted to hospital: implications for management guidelines. *Thorax.* **2001**, 56, 296–301.
57. Logroscino, CD.; Penza, O.; Locicero, S.; Losito, G.; Nardini, S.; Bertoli, L.; Cioffi, R.; Del Prato, B. Community-acquired pneumonia in adults: a multicentric observational AIPO study. *Monaldi. Arch. Chest. Dis.* **1999**, 54, 11–17.
58. Lorente, M.; Falguera, M.; Nogues, A.; González, AR.; Merino, M.; Caballero, MR. Diagnosis of pneumococcal pneumonia by polymerase chain reaction (PCR) in whole blood: a prospective clinical study. *Thorax* 2000, 55: 133–137.

59. Madeddu, G.; Porqueddu, EM.; Cambosu, F.; Saba, F.; Fois, AG.; Pirina, P.; Mura, MS. Bacterial community acquired pneumonia in HIV-infected inpatients in the highly active antiretroviral therapy era. *Infection.* **2008**, 36: 231–236.
60. Maltezou, HC.; La-Scola, B.; Astra, H.; Constantopoulou, I.; Vlahou, V.; Kafetzis, DA.; Constantopoulos, AG.; Raoult, D. Mycoplasma pneumoniae and Legionella pneumophila in community-acquired lower respiratory tract infections among hospitalized children: diagnosis by real time PCR. *Scand. J. Infect. Dis.* **2004**, 36, 639–642.
61. Marcos, M.; de Anta, MJ.; De La Bellacasa, J.; Gonzalez, J.; Martinez, E.; Garcia, E.; Mensa, J.; De Roux, A.; Torres, A. Rapid urinary antigen test for diagnosis of pneumococcal community-acquired pneumonia in adults. *Eur. Respir. J.* **2003**, 21, 209–214.
62. Martinez, D.; Alvarez Rodriguez, V.; Martinez Ortiz de Zarate, M.; Rivas, M.; Gimenez, MJ.; Aguilar, L.; Ruiz Polaina, MJ.; Barberan, J.; Prieto, J. Management in the emergency room of patients requiring hospital treatment of community-acquired pneumonia. *Rev. Esp. Quimioter.* **2009**, 22, 4–9.
63. Martinez-Moragon, E.; Ferrer, LG.; Sanchos, BS.; Fabrellas, EF.; Belda, AG.; Pardo, RJ. Community-acquired pneumonia among the elderly: differences between patients living at home and in nursing homes. *Arch. Bronconeumol.* **2004**, 40: 547–552.
64. Maurin, M.; Hammer, L.; Gestin, B.; Timsit, JF.; Rogeaux, O.; Delavena, F.; Tous, J.; Epaulard, O.; Brion, JP.; Croizé, J. Quantitative real-time PCR tests for diagnostic and prognostic purposes in cases of legionellosis. *Clin. Microbiol. Infect.* **2010**, 16, 379–384.
65. Mena-Bernal, JH.; Mena-Bernal, CH.; Sarmiento, EG.; Gómez, AA.; Santos, SM.; Gaviria, AZ. Adecuación de los ingresos y de la asistencia facilitada a los pacientes con neumonía adquirida en la comunidad. *Rev. Clín. Esp.* **2011**, 211, 179–186.
66. Menéndez, R.; Sahuquillo-Arce, JM.; Reyes, S.; Martínez, R.; Polverino, E.; Cillóniz, C.; Córdoba, JG.; Montull, B.; Torres, A. Cytokine activation patterns and biomarkers are influenced by microorganisms in community-acquired pneumonia. *Chest.* **2012**, 141, 1537–1545.
67. Menendez, R.; Cordoba, J.; de la Cuadra, P.; Cremades, MJ.; Lopez-Hontagas, JL.; Salavert, M.; Gobernado, M. Value of the polymerase chain reaction assay in noninvasive respiratory samples for diagnosis of community-acquired pneumonia. *Am. J. Respir. Crit. Care. Med.* **1999**, 159, 1868–1873.
68. Michetti, G.; Pugliese, C.; Bamberga,M.; Belometti, O.; Villa, R.; Maggi, L.; Ranzanici, S.; Perani, V.; Farina, C.; Moioli, F. Community-acquired pneumonia: is there difference in etiology between hospitalized and out-patients? *Minerva. Med.* **1995**, 86, 341–351.
69. Mirete, FC.; Gutiérrez, RF.; Rodríguez, DJ.; Royo, GG.; Shum, FC.; Martín, HA. Etiology of community-acquired pneumonia in ambulatory patients. Usefulness of a diagnostic investigation protocol using detection of Streptococcus pneumoniae and Legionella pneumophila antigens in urine samples. *Med. Clin.* **2001**, 117, 657–659.
70. Molinos, L.; Clemente, M.; Miranda, B.; Spilotros, G.; Barbuti, G.; Barbuti, S. Community-acquired pneumonia in patients with and without chronic obstructive pulmonary disease. *J. Infect.* **2009**, 58, 417–424.
71. Molinos, L.; Fernandez, R.; Dominguez, MJ.; Riesgo, C.; Escudero, C.; Martinez, J. Adenosine deaminase activity in the aetiological diagnosis of community-acquired pneumonia. *Scand. J. Infect. Dis.* **1997**, 29, 287–290.
72. Montagna, MT.; Napoli, C.; Tato, D.; Spilotros, G.; Barbuti, G.; Barbuti, S. Clinical-environmental surveillance of legionellosis: an experience in Southern Italy. *Eur. J. Epidemiol.* **2006**, 21, 325–331.
73. Mothes, A.; Léotard, S.; Nicolle, I.; Smets, A.; Chirio, D.; Rotomondo, C.; Tiger, F.; Del Giudice, P.; Perrin, C.; Néri, D.; Foucault, C. Community-acquired pneumonia and positive urinary antigen tests: Factors associated with targeted antibiotic therapy. *Med Mal Infect* 2016; **46**: 365–371.
74. Neocleous, C.; Gerogianni, I.; Gourgoulianis, K.; Petinaki, E. Prevalence of atypical bacterial pathogens in hospitalised adult patients with community-acquired pneumonia in Central Greece. *Indian. J. Med. Microbiol.* **2014**, 32, 204–205.
75. Norrby, SR. Atypical pneumonia in the Nordic countries: aetiology and clinical results of a trial comparing fleroxacin and doxycycline. Nordic Atypical Pneumonia Study Group. *J. Antimicrob. Chemother.* **1997**, 39, 499–508.
76. Olaechea, P.; Quintana, J.; Gallardo, M.; Insausti, J.; Maravi, E.; Alvarez, B. A predictive model for the treatment approach to community-acquired pneumonia in patients needing ICU admission. *Intensive. Care. Med.* **1996**, 22, 1294–1300.
77. Pereira, JM.; Teixeira-Pinto, A.; Basílio, C.; Sousa-Dias, C.; Mergulhão, P.; Paiva, JA. Can we predict pneumococcal bacteremia in patients with severe community-acquired pneumonia? *J. Crit. Care.* **2013**, 28, 970–974.

78. Pletz, MW.; Terkamp, C.; Schumacher, U.; Rohde, G.; Schütte, H.; Welte, T.; Bals, R. Vitamin D deficiency in community-acquired pneumonia: low levels of 1, 25 (OH) 2 D are associated with disease severity. *Respir. Res.* **2014**, 15, 1–8.
79. Pradelli, J.; Risso, KD.; de Salvador, FG.; Cua, E.; Ruimy, R.; Roger, P-M. Community-acquired pneumonia: impact of empirical antibiotic therapy without respiratory fluoroquinolones nor third-generation cephalosporins. *Eur J Clin. Microbiol. Infect. Dis.* **2015**, 34, 511–518.
80. Prat, C.; Domínguez, J.; Andreo, F.; Blanco, S.; Pallarés, A.; Cuchillo, F.; Ramil, C.; Ruiz-Manzano, J.; Ausina, V. Procalcitonin and neopterin correlation with aetiology and severity of pneumonia. *J. Infec.t* **2006**, 52, 169–177.
81. Putinati, S.; Ballerin, L.; Piattella, M.; Zabini, F.; Potena, A. Community-acquired pneumonia in the elderly: etiology, clinical aspects and prognostic factors. *Minerva Pneumol* 2004; **43**: 99–106.
82. Reissig, A.; Mempel, C.; Schumacher, U.; Copetti, R.; Gross, F.; Aliberti, S. Microbiological diagnosis and antibiotic therapy in patients with community-acquired pneumonia and acute COPD exacerbation in daily clinical practice: comparison to current guidelines. *Lung*. **2013**, 191, 239–246.
83. Rello, J.; Bodi, M.; Mariscal, D.; Navarro, M.; Diaz, E.; Gallego, M.; Valles, J. Microbiological testing and outcome of patients with severe community-acquired pneumonia. *Chest.* **2003**, 123, 174–180.
84. Rello, J.; Rodriguez, R.; Jubert, P.; Alvarez, B. Severe community-acquired pneumonia in the elderly: epidemiology and prognosis. *Clin. Infect. Dis.* **1996**, 23, 723–728.
85. Riquelme, R.; Torres, A.; El-Ebiary, M.; de la Bellacasa, JP.; Estruch, R.; Mensa, J.; Fernández-Solá, J.; Hernández, C.; Rodriguez-Roisin, R. Community-acquired pneumonia in the elderly: a multivariate analysis of risk and prognostic factors. *Am. J. Respir. Crit. Care Med.* **1996**, 154, 1450–1455.
86. Rizzato, G.; Montemurro, L.; Fraioli, P.; Montanari, G.; Fanti, D.; Pozzoli, R.; Magliano, E. Efficacy of a three day course of azithromycin in moderately severe community-acquired pneumonia. *Eur. Respir. J.* **1995**, 8, 398–402.
87. Roed, T.; Schønheyder, HC.; Nielsen, H. Predictors of positive or negative legionella urinary antigen test in community-acquired pneumonia. *Infect. Dis.* **2015**, 47, 484–490.
88. Roson, B.; Carratala, J.; Verdaguer, R.; Dorca, J.; Manresa, F.; Gudiol, F. Prospective study of the usefulness of sputum Gram stain in the initial approach to community-acquired pneumonia requiring hospitalization. *Clin. Infect. Dis.* **2000**, 31, 869–874.
89. Rosón, B.; Carratalà, J.; Dorca, J.; Casanova, A.; Manresa, F.; Gudiol, F. Etiology, reasons for hospitalization, risk classes, and outcomes of community-acquired pneumonia in patients hospitalized on the basis of conventional admission criteria. *Clin. Infect. Dis.* **2001**, 33, 158–165.
90. Rovira, E.; Martinez-Moragon, E.; Belda, A.; Gonzalvo, F.; Ripollés, F.; Pascual, J. Treatment of community-acquired pneumonia in outpatients: randomized study of clarithromycin alone versus clarithromycin and cefuroxime. *Respiration.* **1999**, 66, 413–418.
91. Røysted, W.; Simonsen, Ø.; Jenkins, A.; Sarjomaa, M.; Svendsen, MV.; Ragnhildstveit, E.; Tveten, Y.; Kanestrøm, A.; Waage, H.; Ringstad, J. Aetiology and risk factors of community-acquired pneumonia in hospitalized patients in Norway. *Clin. Respir. J.* **2016**, 10, 756–764.
92. Ruiz, M.; Ewig, S.; Torres, A.; Arancibia, F.; Marco, F.; Mensa, J.; Sanchez, M.; Martinez, JA. Severe community-acquired pneumonia: risk factors and follow-up epidemiology. *Am. J. Respir. Crit. Care Med.* **1999**, 160, 923–929.
93. Sahuquillo-Arce, JM.; Menéndez, R.; Méndez, R.; Amara-Elori, I.; Zalacain, R.; Capelastegui, A.; Aspa, J.; Borderías, L.; Martín-Villasclaras, JJ.; Bello, S.; Alfageme, I. Age-related risk factors for bacterial aetiology in community-acquired pneumonia. *Respirology* **2016**, 21, 1472–1479.
94. Sangil, A.; Calbo, E.; Robles, A.; Benet, S.; Viladot, ME.; Pascual, V.; Cuchí, E.; Pérez, J.; Barreiro, B.; Sánchez, B.; Torres, J. Aetiology of community-acquired pneumonia among adults in an H1N1 pandemic year: the role of respiratory viruses. *Eur. J. Clin. Microbiol. Infect. Dis.* **2012**, 31, 2765–2772.
95. Santos, J.; Aguilar, L.; Garcia-Mendez, E.; Siquier, B.; Custardoy, J.; García-Rey, C.; Pallarés, R. Clinical characteristics and response to newer quinolones in Legionella pneumonia: a report of 28 cases. *J. Chemother.* **2003**, 15, 461–465.
96. Santos de Unamuno C, Llorente San Martin MA. [Site of care provision, etiology and treatment of community-acquired pneumonia in Palma de Mallorca]. *Med. Clin.* **1998**, 110, 290–294.
97. Sayan, M.; Kilinc, O.; Yüce, A.;,Uncan, E.; Genc, S. [Seropositivity against atypical pneumonia agents demonstrated in patients with community-acquired pneumonia]. *Mikrobiyol. Bul.* **2003**, 37, 247–253.

98. Schneeberger, PM.; Dorigo-Zetsma, J.; van der Zee, A.; van Bon, M.; van Opstal, J. Diagnosis of atypical pathogens in patients hospitalized with community-acquired respiratory infection. *Scand. J. Infect. Dis.* **2004**, 36, 269–273.
99. Sever, F.; Kömüs, N.; Esen, N.; Gündüz, AT.; Öktem, MA.; Çımrın, AH. Türkiye'de Toplum Kökenli Pnömoni Etyoloji ve Epidemiyolojisi. *Turk. Thorac. J.* **2013**, 14, 5–10.
100. Sicras-Mainar, A.; Ibáñez-Nolla, J.; Cifuentes, I.; Guijarro, P.; Navarro-Artieda, R.; Aguilar, L. Retrospective epidemiological study for the characterization of community-acquired pneumonia and pneumococcal pneumonia in adults in a well-defined area of Badalona (Barcelona, Spain). *BMC Infect. Dis.* **2012**, 12, 1–9.
101. Socan, M.; Marinic-Fiser, N.; Kraigher, A.; Kotnik, A.; Logar, M. Microbial aetiology of community-acquired pneumonia in hospitalised patients. *Eur. J. Clin. Microbiol. Infect. Dis.* **1999**, 18, 777–782.
102. Sopena, N.; Sabria-Leal, M.; Pedro-Botet, ML.; Padilla, E.; Dominguez, J.; Morera, J.; Tudela, P. Comparative study of the clinical presentation of Legionella pneumonia and other community-acquired pneumonias. *Chest.* **1998**, 113, 1195–1200.
103. Sousa, D.; Justo, I.; Dominguez, A.; Manzur, A.; Izquierdo, C.; Ruiz, L.; Nebot, M.; Bayas, JM.; Celorrio, JM.; Varona, W.; Llinares, P. Community-acquired pneumonia in immunocompromised older patients: incidence, causative organisms and outcome. *Clin. Microbiol. Infect.* **2013**, 19, 187–192.
104. Spoorenberg, SM.; Bos, WJ.; Heijligenberg, R.; Voorn, PG.; Grutters, JC.; Rijkers, GT.; van de Garde, EM. Microbial aetiology, outcomes, and costs of hospitalisation for community-acquired pneumonia; an observational analysis. *BMC Infect. Dis.* **2014**, 14, 1–9.
105. Steinhoff, D.; Lode, H.; Ruckdeschel, G.; Heidrich, B.; Rolfs, A.; Fehrenbach, FJ.; Mauch, H.; Höffken, G.; Wagner, J. Chlamydia pneumoniae as a cause of community-acquired pneumonia in hospitalized patients in Berlin. *Clin. Infect. Dis.* **1996**, 22, 958–964.
106. Stralin, K.; Olcen, P.; Tornqvist, E.; Holmberg, H. Definite, probable, and possible bacterial aetiologies of community-acquired pneumonia at different CRB-65 scores. *Scand. J. Infect. Dis.* **2010**, 42, 426–434.
107. Tazón-Varela, M.; Alonso-Valle, H.; Muñoz-Cacho, P.; Gallo-Terán, J.; Piris-García, X.; Pérez-Mier, L. Aumento de microorganismos no habituales en la neumonía adquirida en la comunidad. *Semergen.* **2017**, 43, 437–444.
108. Templeton, KE.; Scheltinga, SA.; van den Eeden, WC.; Graffelman, WA.; van den Broek, PJ.; Claas, EC. Improved diagnosis of the etiology of community-acquired pneumonia with real-time polymerase chain reaction. *Clin. Infect. Dis.* **2005**, 41, 345–351.
109. Tilley, R.; Cole, S.; Olver, W. Microbiological investigation of severe community-acquired pneumonia in ICU. *Brit. J. Intensive. Care.* **2009**; 19, 18–20.
110. Torres, A.; Dorca, J.; Zalacain, R.; Bello, S.; El-Ebiary, M.; Molinos, L.; Arevalo, M.; Blanquer, J.; Celis, R.; Iriberri, M.; Prats, E. Community-acquired pneumonia in chronic obstructive pulmonary disease: a Spanish multicenter study. *Am. J. Respir. Crit. Care. Med.* **1996**, 154, 1456–1461.
111. van de Garde, EM.; Endeman, H.; van Hemert, RN.; Voorn, GP.; Deneer, VH.; Leufkens, HG.; van den Bosch, JM.; Biesma, DH. Prior outpatient antibiotic use as predictor for microbial aetiology of community-acquired pneumonia: hospital-based study. *Eur. J. Clin. Pharmacol.* **2008**, 64: 405–410.
112. van der Eerden, MM.; Vlaspolder, F.; de Graaff, CS.; Groot, T.; Jansen, HM.; Boersma, WG. Value of intensive diagnostic microbiological investigation in low- and high-risk patients with community-acquired pneumonia. *Eur. J. Clin. Microbiol. Infect. Dis.* **2005**, 24, 241–249.
113. van Gageldonk-Lafeber, A.; Wever, P.; van der Lubben, I.; De Jager, CP.; Meijer, A.; De Vries, MC.; Elberse, K.; van der Sande, MA.; van der Hoek, W. The aetiology of community-acquired pneumonia and implications for patient management. *Neth. J. Med.* **2013**, 71, 418–425.
114. Vila-Corcoles, A.; Ochoa-Gondar, O.; Rodriguez-Blanco, T.; Raga-Luria, X.; Gomez-Bertomeu, F. Epidemiology of community-acquired pneumonia in older adults: a population-based study. *Respir. Med.* **2009**, 103, 309–316.
115. von Baum, H.; Ewig, S.; Marre, R.; Suttorp, N.; Gonschior, S.; Welte, T.; Lück, C. Community-acquired Legionella pneu-monia: new insights from the German competence network for community acquired pneumonia. *Clin. Infect. Dis.* **2008**, 46, 1356–1364.
116. Walden, AP.; Clarke, GM.; McKechnie, S.; Hutton, P.; Gordon, AC.; Rello, J.; Chiche, JD.; Stueber, F.; Garrard, CS.; Hinds, CJ. Patients with community acquired pneumonia admitted to European intensive care units: an epidemiological survey of the GenOSept cohort. *Crit. Care.* **2014**, 18, 1–9.

117. Zalacain, R.; Torres, A.; Celis, R.; Blanquer, J.; Aspa, J.; Esteban, L.; Menendez, R.; Blanquer, R.; Borderias, L. Community-acquired pneumonia in the elderly: Spanish multicentre study. *Eur. Respir. J.* **2003**, 21, 294–302.
118. Zer, Y.; Bayram, N.; Balci, I.; Filiz, A. Investigation of the causative agents for community-acquired pneumonia in adult patients. *Turk. J. Med. Sci.* **2010**, 40, 47–52.
119. Al-Ali, MK.; Batchoun, RG.; Al-Nour, TM. Etiology of community-acquired pneumonia in hospitalized patients in Jordan. *Saudi. Med. J.* **2006**; 27, 813–816.
120. Alzeer, A.; Mashlah, A.; Fakim, N.; Al-Sugair, N.; Al-Hedaithy, M.; Al-Majed, S.; Jamjoom, G. Tuberculosis is the commonest cause of pneumonia requiring hospitalization during Hajj (pilgrimage to Makkah). *J. Infect.* **1998**, 36, 303–306.
121. Asghar, AH.; Ashshi, AM.; Azhar, EI.; Bukhari, SZ.; Zafar, TA.; Momenah, AM. Profile of bacterial pneumonia during Hajj. *Indian. J. Med. Res.* **2011**, 133, 510.
122. Behbehani, N.; Mahmood, A.; Mokaddas, EM.; Bittar, Z.; Jayakrishnan, B.; Khadadah, M.; Pacsa, AS.; Dhar, R.; Chugh, TD. Significance of atypical pathogens among community-acquired pneumonia adult patients admitted to hospital in Kuwait. *Med. Princ. Pract.* **2005**, 14, 235–240.
123. Ben-Dror, G.; Mizerizky, Y.; Viar, G.; Zuker, M.; Miron, D. [The epidemiology and clinical features of Legionella pneu-monia (LP) in patients older than 60 years old who were hospitalized with pneumonia in northern Israel]. *Harefuah.* **2002**, 141, 680–682.
124. Dahmash, NS.; Chowdhury, M. Re-evaluation of pneumonia requiring admission to an intensive care unit: a prospective study. *Thorax.* **1994**, 49, 71–76.
125. Doaa, A A-A.; Fatma, AA.; Ramadan MN.; Raghda, H. Prevalence of Legionella among pneumonia patients and envi-ronmental water samples in an Egyptian University Hospital. *Int. Arabic. J. Antimicrob. Agents.* **2016**, 6, 1–11.
126. El Sayed Zaki, M.; Goda, T. Clinico-pathological study of atypical pathogens in community-acquired pneumonia: a prospective study. *J. Infect. Dev. Ctries.* **2009**, 3, 199–205.
127. Khedri, F.; Faradonbeh, FA.; Eliyasi, M.; Barghi, A.; Doosti, A.; Emad, P.; Faradonbeh, AA. Molecular depiction of lepa, lida, ralf, rtxa and ivhb virulence factors of legionella pneumophila isolated from respiratory tract infections. *Int Arch. Med.* **2015**, 8, 1–11.
128. Lieberman, D.; Lieberman, D.; Korsonsky, I.; Ben-Yaakov, M.; Lazarovich, Z.; Friedman, MG.; Dvoskin, B.; Leinonen, M.; Ohana, B.; Boldur, I. A comparative study of the etiology of adult upper and lower respiratory tract infections in the community. *Diagn. Microbiol. Infect. Dis.* **2002**, 42, 21–28.
129. Lieberman, D.; Shvartzman, P.; Ben-Yaakov, M.; Lazarovich, Z.; Hoffman, S.; Mosckovitz, R.; Ohana, B.; Leinonen, M.; Luffy, D.; Boldur, I. Etiology of respiratory tract infection in adults in a general practice setting. *Eur. J. Clin. Microbiol. Infect. Dis.* **1998**, 17, 685–689.
130. Lieberman, D.; Schlaeffer, F.; Boldur, I.; Horowitz, S.; Friedman, MG.; Leiononen, M.; Horovitz, O.; Manor, E.; Porath, A. Multiple pathogens in adult patients admitted with community-acquired pneumonia: a one year prospective study of 346 consecutive patients. *Thorax* 1996; **51**: 179–184.
131. Porath, A.; Schlaeffer, F.; Lieberman, D. The epidemiology of community-acquired pneumonia among hospitalized adults. *J. Infect.* **1997**, 34, 41–48.
132. Shibli, F.; Chazan, B.; Nitzan, O.; Flatau, E.; Edelstein, H.; Blondheim, O.; Raz, R.; Colodner, R. Etiology of community-acquired pneumonia in hospitalized patients in northern Israel. *Isr. Med. Assoc. J.* **2010**, 12, 477–482.
133. Tremolieres, F.; Mayaud, C.; Mouton, Y.; Weber, P.; Dellatolas, F.; Caulin, E. [Efficacy and safety of pristinamycin vs amoxicillin in community acquired pneumonia in adults]. *Pathol. Bio. (Paris).* **2005**, 53, 503–510.
134. Álvarez. RF.; Toste, IS.; Cuadrado, GR.; Lana, AT.; Blanco, JG.; Jiménez, A.; Martín, IG. Community-acquired pneumonia: aetiologic changes in a limited geographic area. An 11-year prospective study. *Eur. J. Clin. Microbiol. Infect. Dis.* **2007**, 26, 495–499.
135. Kim, C.; Nyoka, R.; Ahmed, JA.; Winchell, JM.; Mitchell, SL.; Njenga, MK.; Auko, E.; Burton, W.; Breiman, RF.; Eidex, RB. Epidemiology of respiratory infections caused by atypical bacteria in two Kenyan refugee camps. *J. Immigr. Minor Health* **2012**, 14, 140–145.
136. Nyamande, K,. Lalloo, UG. Poor adherence to South African guidelines for the management of community-acquired pneumonia. *S. Afr. Med. J.* **2007**, 97, 601–603.
137. Paganin, F.; Lilienthal, F.; Bourdin, A.; Lugagne, N.; Tixier, F.; Genin, R.; Yvin, JL. Severe community-acquired pneumonia: assessment of microbial aetiology as mortality factor. *Eur. Respir. J.* **2004**, 24, 779–785.

138. Wolter, N.; Carrim, M.; Cohen, C.; Tempia, S.; Walaza, S.; Sahr, P.; De Gouveia, L.; Treurnicht, F.; Hellferscee, O.; Cohen, AL.; Benitez, A. Legionnaires'Disease in South Africa, 2012–2014. *Emerging. Infect. Dis*. **2016**, 22, 131.
139. Bao, Z.; Yuan, X.; Wang, L.; Sun, Y.; Dong, X. The incidence and etiology of community-acquired pneumonia in fever outpatients. *Exp. Biol. Med*. **2012**, 237, 1256–1261.
140. Charles, PG.; Whitby, M.; Fuller, AJ.; Stirling, R.; Wright, AA.; Korman, TM.; Holmes, PW.; Christiansen, KJ.; Waterer, GW.; Pierce, RJ.; Mayall, BC. The etiology of community-acquired pneumonia in Australia: why penicillin plus doxycycline or a macrolide is the most appropriate therapy. *Clin. Infect. Dis.* **2008**, 46, 1513–1521.
141. Chen, L.; Zhou, F.; Li, H.; Xing, X.; Han, X.; Wang, Y.; Zhang, C.; Suo, L.; Wang, J.; Yu, G.; Wang, G. Disease characteristics and management of hospitalised adolescents and adults with community-acquired pneumonia in China: a retrospective multicentre survey. *BMJ Open.* **2018**, 8, e018709.
142. Chen, K.; Jia, R.; Li, L.; Yang, C., Shi, Y. The aetiology of community associated pneumonia in children in Nanjing, China and aetiological patterns associated with age and season. *BMC Public Health* **2015**, 15, 1–6.
143. Horie, M.; Suzuki, M.; Noguchi, S.; Tanaka, W.; Yoshihara, H.; Kawakami, M.; Kichikawa, Y.; Sakamoto, Y.; Ugajin, M. Diagnostic and prognostic value of procalcitonin in community-acquired pneumonia. *Am. J. Medical. Sci.* **2012**, 343, 30–35.
144. How, C-K.; Hou, S-K.; Shih, H-C.; Yen, DH.; Huang, CI.; Lee, CH.; Tang, GJ. Usefulness of triggering receptor expressed on myeloid cells-1 in differentiating between typical and atypical community-acquired pneumonia. *Am. J. Emerg. Med.* **2011**, 29, 626–631.
145. Huang, H.; Zhang, Y.; Xiu, Q.; Zhou, X.; Huang, SG.; Lu, Q.; Wang, DM.; Wang, F. Community-acquired pneumonia in Shanghai, China: microbial etiology and implications for empirical therapy in a prospective study of 389 patients. *Eur. J. Clin. Microbiol. Infect. Dis.* **2006**, 25: 369–374.
146. Huong, PLT.; Hien, PT.; Lan, NTP.; Binh, TQ.; Tuan, DM.; Anh, DD. First report on prevalence and risk factors of severe atypical pneumonia in Vietnamese children aged 1–15 years. *BMC Public Health* **2014**, 14, 1–8.
147. Ishida, T.; Tachibana, H.; Ito, A.; Tanaka, M.; Tokioka, F.; Furuta, K.; Nishiyama, A.; Ikeda, S.; Niwa, T.; Yoshioka, H.; Arita, M. Clinical characteristics of severe community-acquired pneumonia among younger patients: an analysis of 18 years at a community hospital. *J. Infect. Chemother.* **2014**, 20, 471–476.
148. Ishiguro, T.; Takayanagi, N.; Yamaguchi, S.; Yamakawa, H.; Nakamoto, K.; Takaku, Y.; Miyahara, Y.; Kagiyama, N.; Kurashima, K.; Yanagisawa, T.; Sugita, Y. Etiology and factors contributing to the severity and mortality of community-acquired pneumonia. *Intern. Med.* **2013**, 52, 317–324.
149. Jennings, LC.; Anderson, TP.; Beynon, KA.; Chua, A.; Laing, RT.; Werno, AM.; Young, SA.; Chambers, ST.; Murdoch, DR. Incidence and characteristics of viral community-acquired pneumonia in adults. *Thorax.* **2008**, 63, 42–48.
150. Kamata, K.; Suzuki, H.; Kanemoto, K.; Tokuda, Y.; Shiotani, S.; Hirose, Y.; Suzuki, M.; Ishikawa, H. Clinical evaluation of the need for carbapenems to treat community-acquired and healthcare-associated pneumonia. *J. Infect. Chemother.* **2015**, 21, 596–603.
151. Kobashi, Y.; Yoshida, K.; Miyashita, N.; Niki, Y.; Matsushima, T. Evaluating the use of a Streptococcus pneumoniae urinary antigen detection kit for the management of community-acquired pneumonia in Japan. *Respiration.* **2007**, 74, 387–393.
152. Laing, R.; Slater, W.; Coles, C.; Chambers, S.; Frampton, C.; Jackson, R.; Jennings, L.; Karalus, N.; Mills, G.; Murdoch, D.; Town, I. Community-acquired pneumonia in Christchurch and Waikato 1999-2000: microbiology and epidemiology. *NZ. Med. J.* **2001**, 114, 488–492.
153. Lauderdale, TL.; Chang, FY.; Ben, RJ.; Yin, HC.; Ni, YH.; Tsai, JW.; Cheng, SH.; Wang, JT.; Liu, YC.; Cheng, YW.; Chen, ST. Etiology of community acquired pneumonia among adult patients requiring hospitalization in Taiwan. *Respir. Med.* **2005**, 99, 1079–1086.
154. Lee, K.; Hui, K.; Tan, W.; Lim, T. Severe community-acquired pneumonia in Singapore. *Singapore Med. J.* **1996**, 37, 374–377.
155. Liu, Y.; Chen, M. Zhao, T.; Wang, H.; Wang, R.; Liu, QF.; Cai, BQ.; Cao, B.; Sun, TY.; Hu, YJ.; Xiu, QY. [A multicentre study on the pathogenic agents in 665 adult patients with community-acquired pneumonia in cities of China]. *Chin. J. Tuberc. Respir. Dis.* **2006**, 2, 3–8.
156. Liu, Y.; Zhao, T.; Yao, W.; Zhang, LS.; He, ZY.; Jiao, YM.; Duan, YY.; Nie, ZS.; Wang, R.; Liu, QF. [Prevalence of atypical pathogens in adult patients with community-acquired pneumonia in Beijing]. *Chin. J. Tuberc. Respir. Dis.* **2004**, 27, 27–30.

157. Lui, G.; Ip, M.; Lee, N.; Rainer, TH.; Man, SY.; Cockram, CS.; Antonio, GE.; Ng, MH.; Chan, MH.; Chau, SS.; Mak, P. Role of 'atypical pathogens' among adult hospitalized patients with community-acquired pneumonia. *Respirology.* **2009**, 14, 1098–105.
158. Miyashita, N.; Fukano, H.; Mouri, K.; Fukuda, M.; Yoshida, K.; Kobashi, Y.; Niki, Y.; Oka, M. Community-acquired pneumonia in Japan: a prospective ambulatory and hospitalized patient study. *J. Med. Microbiol.* **2005**, 54, 395–400.
159. Neill, AM.; Martin, IR.; Weir, R.; Anderson, R.; Chereshsky, A.; Epton, MJ.; Jackson, R.; Schousboe, M.; Frampton, C.; Hutton, S.; Chambers, ST. Community acquired pneumonia: aetiology and usefulness of severity criteria on admission. *Thorax.* **1996**, 51, 1010–1016.
160. Richards, DA.; Toop, LJ.; Epton, MJ.; Town, GI.; Dawson, RD.; Hlavac, MC.; McGeoch, GR.; Wynn-Thomas, SM.; Abernethy, PD.; Werno, AM. Home management of mild to moderately severe community-acquired pneumonia: a randomised controlled trial. *Med. J. Aust.* **2005**, 183, 235–238.
161. Saito, A.; Kohno, S.; Matsushima, T.; Watanabe, A.; Oizumi, K.; Yamaguchi, K.; Oda, H. Prospective multicenter study of the causative organisms of community-acquired pneumonia in adults in Japan. *J. Infect. Chemother.* **2006**, 12, 63–69.
162. Shindo, Y.; Ito, R.; Kobayashi, D.; Ando, M.; Ichikawa, M.; Shiraki, A.; Goto, Y.; Fukui, Y.; Iwaki, M.; Okumura, J.; Yamaguchi, I. Risk factors for drug-resistant pathogens in community-acquired and healthcare-associated pneumonia. *Am. J. Respir. Crit. Care Med.* **2013**, 188, 985–995.
163. Sohn, JW.; Park, SC.; Choi, YH.; Woo, HJ.; Cho, YK.; Lee, JS.; Sim, HS.; Kim, MJ. Atypical pathogens as etiologic agents in hospitalized patients with community-acquired pneumonia in Korea: a prospective multi-center study. *J. Korean. Med. Sci.* **2006**, 21, 602–607.
164. Song, JH.; Oh, WS.; Kang, CI.; Chung, DR.; Peck, KR.; Ko, KS.; Yeom, JS.; Kim, CK.; Kim, SW.; Chang, HH.; Kim YS. Epidemiology and clinical outcomes of community-acquired pneumonia in adult patients in Asian countries: a prospective study by the Asian network for surveillance of resistant pathogens. *Int. J. Antimicrob. Agents.* **2008**, 31, 107–114.
165. Su H-P, Tseng L-R, Chou C-Y, Chung T-C, Pan T-M. Legionella pneumophila infection in the Taiwan area. *J. Infect. Chemother.* **2005**, 11, 244–249.
166. Takaki, M.; Nakama, T.; Ishida, M.; Morimoto, H.; Nagasaki, Y.; Shiramizu, R.; Hamashige, N.; Chikamori, M.; Yoshida, L.; Ariyoshi, K.; Suzuki, M. High incidence of community-acquired pneumonia among rapidly aging population in Japan: a prospective hospital-based surveillance. *Jpn. J. Infect. Dis.* **2014**, 67: 269–275.
167. Takayanagi, N.; Hara, K.; Tokunaga, D.; Takaku, Y.; Minagawa, S.; Tsuchiya, Y.; Hijikata, N.; Yamaji, T.; Saito, H.; Ubukata, M.; Kurashima, K. [Etiology and outcome of community-acquired pneumonia in relation to age and severity in hospitalized adult patients]. *J. Jpn. Respir. Soc.* **2006**, 44, 906–915.
168. Tao, L.; Hu, B.; He, L.; Li, WE.; Xie, HM.; Wang, BQ.; Li, HY. Etiology and antimicrobial resistance of community-acquired pneumonia in adult patients in China. *Chin. Med. J.* **2012**, 125, 2967–2972.
169. Ugajin, M.; Yamaki, K.; Hirasawa, N.; Yagi, T. Predictive values of semi-quantitative procalcitonin test and common biomarkers for the clinical outcomes of community-acquired pneumonia. *Respir. Care.* **2014**, 54, 564–573.
170. Watari, M.; Komagata, H.; Ohe, M.; Kunimoto, E.; Tsukamoto, R. Comparative study of clinical features of typical and atypical pneumonias. *J. Jpn. Respir. Soc.* **1999**, 37, 388–395.
171. Wilson, PA.; Ferguson, J. Severe community-acquired pneumonia: an Australian perspective. *Intern. Med. J.* **2005**, **35**: 699–705.
172. Xiong, Y.; Deng, R.; Fu, R.; Li, DZ.; Chen, J.; Chen, YS.; Hu, CP.; Li, JS.; Wang, RQ.; Wei, LP.; Zhong, XN. Atypical pathogens in adult patients admitted with community-acquired pneumonia. *Chin. J. Tuberc. Respir. Dis.* **2010**, 33, 646–650.
173. Yeh, K.; Lin, J.; Wang, N.; Chang, F. Legionnaires' disease in community-acquired pneumonia requiring hospitalization in Taiwan. *J. Med. Sciences-Taipei.* **2007**, 27, 63.
174. Yen, M-Y.; Hu, B-S.; Chen, Y-S.; Lee, SS.; Lin, YS.; Wann, SR.; Tsai, HC.; Lin, HH.; Huang, CK.; Liu, YC. A prospective etiologic study of community-acquired pneumonia in Taiwan. *J. Formos. Med. Assoc.* **2005**, 104, 724–730.
175. Yoshimoto, A.; Nakamura, H.; Fujimura, M.; Nakao, S. Severe community-acquired pneumonia in an intensive care unit: risk factors for mortality. *Intern. Med.* **2005**, 44, 710–716.
176. Zhang, L.; Ning, P.; Rong, F.; Gao, Z. Change of prognosis and pathogens in adult patients with severe community-acquired pneumonia during different periods. *Zhonghua Yi Xue Za Zhi.* **2017**, 97, 332–337.

177. Anbumani, S.; Gururajkumar, A.; Chaudhury, A. Isolation of Legionella pneumophila from clinical & environmental sources in a tertiary care hospital. *Indian. J. Med. Res.* **2010**, 131, 761–764
178. Angrup, A.; Chaudhry, R.; Sharma, S.; Valavane, A.; Passi, K.; Padmaja, K.; Javed, S.; Dey, AB.; Dhawan, B.; Kabra, SK. Application of real-time quantitative polymerase chain reaction assay to detect Legionella pneumophila in patients of community-acquired pneumonia in a tertiary care hospital. *Indian J. Med. Microbiol.* **2016**, 34, 539–543.
179. Chaudhry, R.; Dhawan. B.; Dey, AB. The incidence of Legionella pneumophila: a prospective study in a tertiary care hospital in India. *Trop. Dr.* **2000**, 30, 197–200.
180. Javed S, Chaudhry R, Passi K, Sharma, S.; Padmaja, K.; Dhawan, B.; Dey, AB. Sero diagnosis of Legionella infection in community acquired pneumonia. *Indian J. Med. Res.* **2010**, 131, 92–96.
181. Liam, CK.; Pang, YK.; Poosparajah, S. Pulmonary tuberculosis presenting as community-acquired pneumonia. *Respirology.* **2006**, 11, 786–792.
182. Mustafa, MI.; Al-Marzooq, F.; How, SH.; Kuan, YC.; Ng, TH. The use of multiplex real-time PCR improves the detection of the bacterial etiology of community acquired pneumonia. *Trop. Biomed.* **2011**, 28, 531–544.
183. Nadar, S.; Hoque, S.; Brahmadattam, KN.; Mathai, D. A hospital based study of Community acquired mycoplasma and legionella lower respiratory tract infections in Southern Indian. *Indian. Pract.* 1997; **50**: 481–6.
184. Ngeow, YF.; Suwanjutha, S.; Chantarojanasriri, T.; Wang, F.; Saniel, M.; Alejandria, M.; Hsueh, PR.; Ping-Ing, L.; Park, SC.; Sohn, JW.; Aziah, AM. An Asian study on the prevalence of atypical respiratory pathogens in community-acquired pneumonia. *Int. J. Infect. Dis.* **2005**, 9, 144–153.
185. Olsen, SJ.; Thamthitiwat, S.; Chantra, S.; Chittaganpitch, M.; Fry, AM.; Simmerman, JM.; Baggett, HC.; Peret, TC.; Erdman, D.; Benson, R.; Talkington, D. Incidence of respiratory pathogens in persons hospitalized with pneumonia in two provinces in Thailand. *Epidemiol. Infect.* **2010**, 138, 1811–1522.
186. Phares, CR.; Wangroongsarb, P.; Chantra, S.; Paveenkitiporn, W.; Tondella, ML.; Benson, RF.; Lanier Thacker, W.; Fields, BS.; Moore, MR.; Fischer, J.; Dowell, SF. Epidemiology of severe pneumonia caused by Legionella longbeachae, Mycoplasma pneumoniae, and Chlamydia pneumoniae: 1-year, population-based surveillance for severe pneumonia in Thailand. *Clin. Infect. Dis.* **2007**, 45, e147–e55.
187. Prapphal, N.; Suwanjutha, S.; Durongkaveroj, P.; Lochindarat, MD.; Kunakorn, M.; Deerojanawong, J.; Chantarojanasiri, T.; Supanitayaonon, Y.; Janedittakarn, P. Prevalence and clinical presentations of atypical pathogens infection in community acquired pneumonia in Thailand. *J. Med. Assoc. Thai.* **2006**, 89, 1412–1419.
188. Wattanathum, A.; Chaoprasong, C.; Nunthapisud, P.; Chantaratchada, S.; Limpairojn, N.; Jatakanon, A.; Chanthadisai, N. Community-acquired pneumonia in southeast Asia: the microbial differences between ambulatory and hospitalized patients. *Chest.* **2003**, 123, 1512–1519.
189. Arancibia, F.; Cortes, CP.; Valdes, M.; Cerda, J.; Hernández, A.; Soto, L.; Torres, A. Importance of Legionella pneumophila in the etiology of severe community-acquired pneumonia in Santiago, Chile. *Chest*. **2014**, 145, 290–296.
190. Bellew, S.; Grijalva, CG.; Williams, DJ.; Anderson, EJ.; Wunderink, RG.; Zhu, Y.; Waterer, GW.; Bramley, AM.; Jain, S.; Edwards, KM.; Self, WH. Pneumococcal and Legionella urinary antigen tests in community-acquired pneumonia: Prospective evaluation of indications for testing. *Clin. Infect. Dis.* **2018**, 68, 2026–2033.
191. Caberlotto, OJ.; Cadario, ME.; Garay, JE.; Copacastro, CA.; Cabot, A.; Savy, VL. [Community-acquired pneumonia in patients in 2 hospital populations]. *Medicina*. **2003**, 63, 1–8.
192. Chedid, MB.; Ilha Dde, O.; Chedid, MF.; Dalcin, PR.; Buzzetti, M.; Saraiva, PJ.; Griza, D.; Barreto, SS. Community-acquired pneumonia by Legionella pneumophila serogroups 1-6 in Brazil. *Respir. Med.* **2005**, 99, 966–975.
193. Corrêa, R.; Lopes, R.; Oliveira, L.; Campos, F.; Reis, M.; Rocha, M. Estudo de casos hospitalizados por pneumonia comunitária no período de um ano. *Pneumologia.* **2001**, 27, 243–248.
194. Diaz, A.; Barria, P.; Niederman, M.; Dreyse, J.; Fuentes, G.; Couble, B.; Saldias, F. Etiology of community-acquired pneumonia in hospitalized patients in chile: the increasing prevalence of respiratory viruses among classic pathogens. *Chest.* **2007**, 131, 779–787.
195. El-Solh, AA.; Sikka, P.; Ramadan, F.; Davies, J. Etiology of severe pneumonia in the very elderly. *Am. J. Respir. Crit. Care. Med.* **2001**, 163, 645–651.
196. Gelfer, G.; Leggett, J.; Myers, J.; Wang, L.; Gilbert, DN. The clinical impact of the detection of potential etiologic pathogens of community-acquired pneumonia. *Diagn. Microbiol. Infect. Dis.* **2015**, 83, 400–406.
197. Harris, AM.; Bramley, AM.; Jain, S.; Arnold, SR.; Ampofo, K.; Self, WH.; Williams, DJ.; Anderson, EJ.; Grijalva, CG.; McCullers, JA.; Pavia, AT. Influence of antibiotics on the detection of bacteria by culture-based and culture-

independent diagnostic tests in patients hospitalized with community-acquired pneumonia. *Open. Forum. Infect. Dis.* **2017**, 4, ofx014.
198. Henry, C.; Boethel, C.; Copeland, LA.; Ghamande, S.; Arroliga, AC.; White, HD. Clinical Utility of Testing for Legionella Pneumonia in Central Texas. *Ann. Am. Thorac. Soc.* **2017**, 14, 65–69.
199. Hollenbeck, B.; Dupont, I.; Mermel, LA. How often is a work-up for Legionella pursued in patients with pneumonia? a retrospective study. *BMC. Infect. Dis.* **2011**, 11, 1–4.
200. Jain, S.; Self, WH.; Wunderink, RG.; Fakhran, S.; Balk, R.; Bramley, AM.; Reed, C.; Grijalva, CG.; Anderson, EJ.; Courtney DM.; Chappell, JD. Community-acquired pneumonia requiring hospitalization among US adults. *N. Engl. J. Med*. **2015**, 373, 415–427.
201. Johnstone, J.; Majumdar, SR.; Fox, JD.; Marrie, TJ. Viral Infection in Adults Hospitalized With Community-Acquired Pneumonia Prevalence, Pathogens, and Presentation. *Chest*. **2008**, 134, 1141–1148.
202. Lopardo, G.; Sturba, E.; Martinez, M.; Roel, JE.; Gamba, A.; Biondi, H.; Stamboulian, D. [Detection of acute infection due to legionella pneumophila in patient with community acquired pneumonia in Buenos Aires city]. *Medicina.* **2001**, 62, 145–148.
203. Luchsinger, V.; Ruiz, M.; Zunino, E.; Martínez, MA.; Machado, C.; Piedra, PA.; Fasce, R.; Ulloa, MT.; Fink, MC.; Lara, P.; Gebauer, M. Community-acquired pneumonia in Chile: the clinical relevance in the detection of viruses and atypical bacteria. *Thorax.* **2013**, 68, 1000–1006.
204. Luna, CM.; Folco, B.; Aruj, P.; Rebora, K.; Balsebre, C.; Absi, R.; Vay, C.; de Mier, C.; Famiglietti, A. [Pneumonia due to Legionella pneumophila. Experience gathered in a University Hospital in Buenos Aires]. *Medicina.* **2004**, 64, 97–102.
205. Marrie, TJ.; De Carolis, E.; Yu, VL.; Stout, JE. Legionnaires' Disease-Results of a Multicenter Canadian Study. *Can. J. Infect. Dis. Med. Microbiol.* **2003**, 14, 154–158.
206. Marrie, TJ.; Peeling, RW.; Fine, MJ.; Singer, DE.; Coley, CM.; Kapoor, WN. Ambulatory patients with community-acquired pneumonia: the frequency of atypical agents and clinical course. *Am. J. Med.* **1996**, 101, 508–515.
207. Marston, BJ.; Plouffe, JF.; File, Jr TM.; Hackman, BA. Incidence of community-acquired pneumonia requiring hospitalization: Results of a population-based active surveillance study in Ohio. *Arch. Intern. Med.* **1997**, 157, 1709–1718.
208. McNally, C.; Hackman, B.; Fields, BS.; Plouffe, JF. Potential importance of Legionella species as etiologies in community acquired pneumonia (CAP). *Diagn. Microbiol. Infect. Dis.* **2000**, 38, 79–82.
209. Micek, ST.; Kollef, KE.; Reichley, RM.; Roubinian, N.; Kollef, MH. Health care-associated pneumonia and community-acquired pneumonia: a single-center experience. *Antimicrob. Agents. Chemother.* **2007**, 51, 3568–3573.
210. Mundy, LM.; Auwaerter, PG.; Oldach, D.; Warner, ML.; Burton, A.; Vance, E.; Gaydos, CA.; Joseph, JM.; Gopalan, R.; Moore, RD. Community-acquired pneumonia: impact of immune status. *Am J Respir. Crit. Care. Med.* **1995**, 152, 1309–1315.
211. Palombini, B.; Stoltz, P.; Villanova, C. Treatment of community acquired atypical pneumonia (CAP): results of an open study. *Rev. Bras. Med.* **1997**, 54, 54–58.
212. Park, DR.; Sherbin, VL.; Goodman, MS.; Pacifico, AD.; Rubenfeld, GD.; Polissar, NL.; Harborview CAP Study Group. The etiology of community-acquired pneumonia at an urban public hospital: influence of human immunodeficiency virus infection and initial severity of illness. *J. Infect. Dis.* **2001**, 18, 268–277.
213. Peñafiel, F.; Gutiérrez, M.; López, G.; Aranguiz, JME.; Monasterio, JU.; Soto, AM.; Patino, OD. Importance of respiratory virus in immunocompetent adult patients hospitalized with community-acquired pneumonia. *Rev. Med. Chile.* **2016**, 144, 1513–1522.
214. Plouffe, J.; Herbert, M.; File, Jr T.; Baird, I.; Parsons, JN.; Kahn, JB.; Rielly-Gauvin, KT. Ofloxacin versus standard therapy in treatment of community-acquired pneumonia requiring hospitalization. Pneumonia Study Group. *Antimicrob. Agents. Chemother.* **1996**, 40, 1175–1179.
215. Rocha, RT.; Vital, AC.; Silva, C.; Pereira, C.; Nakatani, J. Community-acquired pneumonia in outpatients: epidemiological, clinical and radiographic features between atypical and non-atypical pneumonia. *J. Pneumol.* **2000**, 26, 5–14.
216. Tan, MJ.; Tan, JS.; Hamor, RH.; File, TM.; Breiman, RF. The radiologic manifestations of Legionnaire's disease. *Chest.* **2000**, 117, 398–403.
217. Touray, S.; Newstein, MC.; Lui, JK.; Harris, M.; Knox, K. Legionella pneumophila cases in a community hospital: A 12-month retrospective review. *SAGE. Open. Med.* **2014**, 2, 2050312114554673.

218. Vanina, G.; Aguirre, R.; Lujan, R.; Guzmán, M.; Absi, R.; Vay, C.; Famiglietti, A.; Luna, CM. Neumonía Grave de la Comunidad (NGC), 145 casos internados en terapia intensiva. *Rev. Am. Med. Respir.* **2009**, 9, 181–189.
219. Vergis, EN.; Indorf, A.; File, TM.; Phillips, J.; Bates, J.; Tan, J.; Sarosi, GA.; Grayston, JT.; Summersgill, J.; Victor, LY. Azithromycin vs cefuroxime plus erythromycin for empirical treatment of community-acquired pneumonia in hospitalized patients: a prospective, randomized, multicenter trial. *Arch Intern Med* **2000**, 160, 1294–1300.
220. Carugati, M.; Aliberti, S.; Reyes, LF.; Sadud, RF.; Irfan, M.; Prat, C.; Soni, NJ.; Faverio, P.; Gori, A.; Blasi, F.; Restrepo, MI. Microbiological testing of adults hospitalised with community-acquired pneumonia: an international study. *ERJ Open Res.* **2018**, 4, 00096–2018.
221. Holm, A.; Nexoe, J.; Bistrup, LA.; Pedersen, SS.; Obel, N.; Nielsen, LP. Aetiology and prediction of pneumonia in lower respiratory tract infection in primary care. *Br. J. Gen. Pract.* **2007**, 57, 547–554.
222. Falguera, M.; Carratala, J.; Ruiz-Gonzalez, A.; Garcia-Vidal, C.; Gazquez, I.; Dorca, J.; Gudiol, F.; Porcel, JM. Risk factors and outcome of community-acquired pneumonia due to Gram-negative bacilli. *Respirology.* **2009**, 14, 105–111.
223. Lagerstrom, F.; Bader, M.; Foldevi, M.; Fredlund, H.; Nordin-Olsson, I.; Holmberg, H. Microbiological etiology in clinically diagnosed community-acquired pneumonia in primary care in Orebro, Sweden. *Clin. Microbiol. Infect.* **2003**, 9, 645–652.
224. Naderi, H.; Sheybani, F.; Sarvghad, M.; Meshkat, Z.; Nooghabi, MJ. Etiological diagnosis of community-acquired pneumonia in adult patients: a prospective hospital-based study in Mashhad, Iran. *Jundishapur. J. Microbiol.* **2015**, 8, e22780.
225. Lee, S-J.; Lee, M-G.; Jeon, M-J.; Jung, K-S.; Lee, H-K.; Kishimoto, T. Atypical pathogens in adult patients admitted with community-acquired pneumonia in Korea. *Jpn. J. Infect. Dis.* **2002**, 55, 157–159.
226. Dissanayake, B.; Jayawardena, D.; Senevirathna, C.; Gamage, T. Community acquired pneumonia due to Legionella pneumophila in a tertiary care hospital. *Sri Lankan J. Infect. Dis.* **2016**, 6, 101–105.
227. Kühne, J.; Linhares, TS.; Andreolla, HF.; Stadnik, C.; Pasqualotto, AC. Absence of legionellosis in patients with community-acquired pneumonia requiring hospitalization. *Braz. J. Infect. Dis.* **2014**, 18, 572–573.
228. Musher, DM.; Roig, IL.; Cazares, G.; Stager, CE.; Logan, N.; Safar, H. Can an etiologic agent be identified in adults who are hospitalized for community-acquired pneumonia: results of a one-year study. *J. Infect.* **2013**, 67, 11-18.
229. Alvarez, FG.; García, AF.; Elías, TH.; Romero, JC.; Romero, BR.; Castillo, JG. Community acquired pneumonia in patients older than 60 years. Incidence of atypical agents and clinical-radiological progression. *Med. Clin.* **2001**, 117, 441–445.
230. Arancibia, F.; Bauer, TT.; Ewig, S.; Mensa, J.; Gonzalez, J.; Niederman, MS. Community-acquired pneumonia due to gram-negative bacteria and Pseudomonas aeruginosa: incidence, risk, and prognosis. *Arch. Intern. Med.* **2002**, 162, 1849–1858.
231. Carratalà, J.; Mykietiuk, A.; Fernández-Sabé, N.; Suárez, C.; Dorca, J.; Verdaguer, R.; Manresa, F.; Gudiol, F. Health care–associated pneumonia requiring hospital admission: epidemiology, antibiotic therapy, and clinical outcomes. *Arch. Intern. Med.* **2007**, 167, 1393–1399.
232. Cillóniz, C.; Ewig, S.; Polverino, E.; Marcos, MA.; Prina, E.; Sellares, J.; Ferrer, M.; Ortega, M.; Gabarrús, A.; Mensa, J.; Torres, A. Community-acquired pneumonia in outpatients: aetiology and outcomes. *Eur. Respir. J.* **2012**, 40, 931–938.
233. Cillóniz, C.; Ewig, S.; Polverino, E.; Marcos, MA.; Esquinas, C.; Gabarrús, A.; Mensa, J.; Torres, A. Microbial aetiology of community-acquired pneumonia and its relation to severity. *Thorax.* **2011**, 66, 340–346.
234. Cillóniz, C.; Ewig, S.; Ferrer, M.; Polverino, E.; Gabarrús, A.; de la Bellacasa, JP.; Mensa, J.; Torres, A. Community-acquired polymicrobial pneumonia in the intensive care unit: aetiology and prognosis. *Crit. Care.* **2011**, 15, 1–10.
235. Curran, A.; Falco, V.; Crespo, M.; Martinez, X.; Ribera, E.; Villar del Saz, S.; Imaz, A.; Coma, E.; Ferrer, A.; Pahissa, A. Bacterial pneumonia in HIV-infected patients: use of the pneumonia severity index and impact of current management on incidence, aetiology and outcome. *HIV. Med.* **2008**, 9, 609–615.
236. De Roux, A.; Marcos, MA.; Garcia, E.; Mensa, J.; Ewig, S.; Lode, H.; Torres, A. Viral community-acquired pneumonia in nonimmunocompromised adults. *Chest.* **2004**, 125, 1343–51.
237. Diederen, BM.; Van Der Eerden, MM.; Vlaspolder, F.; Boersma, WG.; Kluytmans, JA.; Peeters, MF. Detection of respiratory viruses and Legionella spp. by real-time polymerase chain reaction in patients with community acquired pneumonia. *Scand. J. Infect. Dis.* **2009**, 41, 45–50.

238. Endeman, H.; Meijvis, S.; Rijkers, G.; van Velzen–Blad, H.; Van Moorsel, CH.; Grutters, JC.; Biesma, DH. Systemic cytokine response in patients with community-acquired pneumonia. *Eur. Respir. J.* **2011**, 37, 1431–1438.
239. Espana, P.; Capelastegui, A.; Bilbao, A.; Diez, R.; Izquierdo, F.; De Goicoetxea, ML.; Gamazo, J.; Medel, F.; Salgado, J.; Gorostiaga, I.; Quintana, JM. Utility of two biomarkers for directing care among patients with non-severe community-acquired pneumonia. *Eur. J. Clin. Microbiol. Infect. Dis.* **2012**, 31, 3397–3405.
240. Ewig, S.; Torres, A.; Angeles Marcos, M.; Angrill, J.; Rano, A.; De Roux, A.; Mensa, J.; Martinez, JA.; de la Bellacasa, JP.; Bauer, T. Factors associated with unknown aetiology in patients with community-acquired pneumonia. *Eur. Respir. J.* **2002**, 20, 1254–1262.
241. Fernández-Sabé, N.; Carratalà, J.; Rosón, B.; Dorca, J.; Verdaguer, R.; Manresa, F.; Gudiol, F. Community-acquired pneumonia in very elderly patients: causative organisms, clinical characteristics, and outcomes. *Medicine*. **2003**, 82, 159–169.
242. Ferrer, M.; Travierso, C.; Cilloniz, C.; Gabarrus, A.; Ranzani, OT.; Polverino, E.; Liapikou, A.; Blasi, F.; Torres, A. Severe community-acquired pneumonia: Characteristics and prognostic factors in ventilated and non-ventilated patients. *PloS One.* **2018**, 13, e0191721.
243. Garcia-Vidal, C.; Carratala, J.; Fernandez-Sabe, N.; Dorca, J.; Verdaguer, R.; Manresa, F.; Gudiol, F. Aetiology of, and risk factors for, recurrent community-acquired pneumonia. *Clin. Microbiol. Infect.* **2009**, 15, 1033–1038.
244. Gómez-Junyent, J.; Garcia-Vidal, C.; Viasus, D.; Millat-Martínez, P.; Simonetti, A.; Santos, MS.; Ardanuy, C.; Dorca, J.; Carratalà, J. Clinical features, etiology and outcomes of community-acquired pneumonia in patients with chronic obstructive pulmonary disease. *PloS One.* **2014**, 9, e105854.
245. Kothe, H.; Bauer, T.; Marre, R.; Suttorp, N.; Welte, T.; Dalhoff, K.; Competence Network for Community-Acquired Pneumonia study group. Outcome of community-acquired pneumonia: influence of age, residence status and antimicrobial treatment. *Eur. Respir. J.* **2008**, 32, 139–146.
246. Lérida, A.; Marrón, A.; Casanova, A.; Roson, B.; Carratala, J.; Gudiol, F. [Respiratory syncytial virus infection in adult patients hospitalized with community-acquired pneumonia]. *Enferm. Infecc. Microbiol. Clin.* **2000**, 18, 177–181.
247. Liapikou, A.; Polverino, E.; Ewig, S.; Cilloniz, C.; Marcos, MA.; Mensa, J.; Bello, S.; Martin-Loeches, I.; Menéndez, R.; Torres, A. Severity and outcomes of hospitalised community-acquired pneumonia in COPD patients. *Eur. Respir. J.* **2012**, 39, 855–861.
248. Marcos, MA.; Camps, M.; Pumarola, T.; Martinez, JA.; Martinez, E.; Mensa, J.; Garcia, E.; Peñarroja, G.; Dambrava, P.; Casas, I.; de Jiménez Anta, MT. The role of viruses in the aetiology of community-acquired pneumonia in adults. *Antivir. Ther.* **2006**, 11, 351.
249. Meijvis, SC.; Hardeman, H.; Remmelts, HH.; Heijligenberg, R.; Rijkers, GT.; van Velzen-Blad, H.; Voorn, GP.; van de Garde, EM.; Endeman, H.; Grutters, JC.; Bos, WJ. Dexamethasone and length of hospital stay in patients with community-acquired pneumonia: a randomised, double-blind, placebo-controlled trial. *Lancet.* **2011**, 377, 2023–2030.
250. Müller, B;. Harbarth, S.; Stolz, D.; Bingisser, R.; Mueller, C.; Leuppi, J.; Nusbaumer, C.; Tamm, M.; Christ-Crain, M. Diagnostic and prognostic accuracy of clinical and laboratory parameters in community-acquired pneumonia. *BMC Infect. Dis.* **2007**, 7, 1–10.
251. Mykietiuk, A.; Carratalà, J.; Fernández-Sabé, N.; Dorca, J.; Verdaguer, R.; Manresa, F.; Gudiol, F. Clinical outcomes for hospitalized patients with Legionella pneumonia in the antigenuria era: the influence of levofloxacin therapy. *Clin. Infect. Dis.* **2005**, 40, 794–799.
252. Perelló, R.; Miró, Ò.; Marcos, MA.; Almela, M.; Bragulat, E.; Sánchez, M.; Agustí, C.; Miro, JM.; Moreno, A. Predicting bacteremic pneumonia in HIV-1–infected patients consulting the ED. *Am. J. Emerg. Med.* **2010**, 28, 454–459.
253. Polverino, E.; Cilloniz, C.; Menendez, R.; Gabarrus, A.; Rosales-Mayor, E.; Alcaraz, V.; Terraneo, S.; de la Bella Casa, JP.; Mensa, J.; Ferrer, M.; Torres, A. Microbiology and outcomes of community acquired pneumonia in non cystic-fibrosis bronchiectasis patients. *J. Infect.* **2015**, 71, 28–36.
254. Raeven, VM.; Spoorenberg, SM.; Boersma, WG.; van de Garde, EM.; Cannegieter, SC.; Voorn, GP.; Bos, WJ.; van Steenbergen, JE. Atypical aetiology in patients hospitalised with community-acquired pneumonia is associated with age, gender and season; a data-analysis on four Dutch cohorts. *BMC Infect. Di.s* **2016**, 16, 1–9.
255. Roson, B.; Carratala, J.; Fernandez-Sabe, N.; Tubau, F.; Manresa, F.; Gudiol, F. Causes and factors associated with early failure in hospitalized patients with community-acquired pneumonia. *Arch. Intern. Med.* **2004**, 164, 502–508.
256. Ruiz, M.; Ewig, S.; Marcos, MA.; Martinez, JA.; Arancibia, F.; Mensa, J.; Torres, A. Etiology of community-acquired pneumonia: impact of age, comorbidity, and severity. *Am. J. Respir. Crit. Care Med.* 1999; **160**, 397–405.

257. Simonetti, A.; Viasus, D.; Garcia-Vidal, C.; Grillo, S.; Molero, L.; Dorca, J.; Carratala, J. Impact of pre-hospital antibiotic use on community-acquired pneumonia. *Clin. Microbiol. Infect.* **2014**, 20, O531–537.
258. Sordé, R.; Falcó, V.; Lowak, M.; Domingo, E.; Ferrer, A.; Burgos, J.; Puig, M.; Cabral, E.; Len, O.; Pahissa, A. Current and potential usefulness of pneumococcal urinary antigen detection in hospitalized patients with community-acquired pneumonia to guide antimicrobial therapy. *Arch. Intern. Med.* **2011**, 171, 166–172.
259. Taylor, JK.; Fleming, GB.; Singanayagam, A.; Hill, AT.; Chalmers, JD. Risk factors for aspiration in community-acquired pneumonia: analysis of a hospitalized UK cohort. *Am. J. Med.* **2013**, 126, 995–1001.
260. van Mens, SP.; Meijvis, SC.; Endeman, H.; van Velzen-Blad, H.; Biesma, DH.; Grutters, JC.; Vlaminckx, BJ.; Rijkers, GT. Longitudinal analysis of pneumococcal antibodies during community-acquired pneumonia reveals a much higher involvement of Streptococcus pneumoniae than estimated by conventional methods alone. *Clin. Vaccine. Immunol.* **2011**, 18, 796–801.
261. Vazquez, EG.; Martinez, JA.; Mensa, J.; Sanchez, F.; Marcos, MA.; De Roux, A.; Torres, A. C-reactive protein levels in community-acquired pneumonia. *Eur. Respir. J.* **2003**, 21, 702–705.
262. Viasus, D.; Di Yacovo, S.; Garcia-Vidal, C.; Verdaguer, R.; Manresa, F.; Dorca, J.; Gudiol, F.; Carratalà, J. Community-acquired Legionella pneumophila pneumonia: a single-center experience with 214 hospitalized sporadic cases over 15 years. *Medicine.* **2013**, 92, 51–60.
263. Viasus, D.; Garcia-Vidal, C.; Castellote, J.; Adamuz, J.; Verdaguer, R.; Dorca, J.; Manresa, F.; Gudiol, F.; Carratala, J. Community-acquired pneumonia in patients with liver cirrhosis: clinical features, outcomes, and usefulness of severity scores. *Medicine.* **2011**, 90, 110–118.
264. Viasus, D.; Garcia-Vidal, C.; Cruzado, JM.; Adamuz, J.; Verdaguer, R.; Manresa, F.; Dorca, J.; Gudiol, F.; Carratala, J. Epidemiology, clinical features and outcomes of pneumonia in patients with chronic kidney disease. *Nephrol. Dial. Transplant.* **2011**, 26, 2899–2906.
265. Lieberman, D.; Lieberman, D.; Ben-Yaakov, M.; Lazarovich, Z.; Ohana, B.; Friedman. MG.; Dvoskin, B.; Leinonen, M.; Boldur, I. Infectious aetiologies in elderly patients hospitalised with non-pneumonic lower respiratory tract infection. *Age and Ageing.* **2003**, 32, 95–101.
266. Ishida, T.; Hashimoto, T.; Arita, M.; Tojo, Y.; Tachibana, H.; Jinnai, M. A 3-year prospective study of a urinary antigen-detection test for Streptococcus pneumoniae in community-acquired pneumonia: utility and clinical impact on the reported etiology. *J Infect. Chemother.* **2004**, 10, 359–363.
267. Ishida, T. [Etiology of community-acquired pneumonia among adult patients in Japan]. *Jap. J Antibiot.* **2000**, 53, 3–12.
268. Ishida, T.; Hashimoto, T.; Arita, M.; Ito, I.; Osawa, M. Etiology of community-acquired pneumonia in hospitalized patients: a 3-year prospective study in Japan. *Chest.* **1998**, 114, 1588–1593.
269. Takayanagi, N.; Tokunaga, D.; Kubota, M.; Hara, K.; Saito, H.; Ubukata, M.; Kurashima, K.; Yanagisawa, T.; Sugita, Y. [Community-acquired pneumonia with rhabdomyolysis]. *Nihon Kokyuki Gakkai zasshi.* **2005**, 43, 731–735.
270. Zhou, Q-t.; He, B.; Zhu, H. Potential for cost-savings in the care of hospitalized low-risk community-acquired pneumonia patients in China. *Value in Health.* **2009**, 12, 40–46.
271. Luna, CM.; Famiglietti, A.; Absi, R.; Videla, AJ.; Nogueira, FJ.; Fuenzalida, AD.; Geneé, RJ. Community-acquired pneumonia: etiology, epidemiology, and outcome at a teaching hospital in Argentina. *Chest* **2000**, 118, 1344–1354.
